# Burden of Peripheral Artery Disease and Its Attributable Risk Factors in 204 Countries and Territories From 1990 to 2019

**DOI:** 10.3389/fcvm.2022.868370

**Published:** 2022-04-12

**Authors:** Jinfeng Lin, Yangbo Chen, Nan Jiang, Zuoshi Li, Shangbo Xu

**Affiliations:** ^1^Department of Neurosurgery, Jieyang People's Hospital, Jieyang, China; ^2^Department of Cardiology, Jieyang People's Hospital, Jieyang, China

**Keywords:** Global Health, peripheral artery disease, epidemiology, incidence, risk factor

## Abstract

**Background:**

Data on burden of peripheral artery disease (PAD) and its attributable risk factors are valuable for policymaking. We aimed to estimate the burden and risk factors for PAD from 1990 to 2019.

**Methods:**

We extracted the data on prevalence, incidence, death, years lived with disability (YLDs), and years of life lost (YLLs) from the Global Burden of Disease Study 2019 to measure PAD burden. Moreover, the attributable burden to PAD risk factors was also estimated.

**Results:**

Globally, in 2019, 113,443,017 people lived with PAD and 10,504,092 new cases occurred, resulting in 74,063 deaths, 500,893 YLDs, and 1,035,487 YLLs. The absolute numbers of PAD prevalent and incident cases significantly increased between 1990 and 2019, contrasting with the decline trends in age-standardized prevalence and incidence rates. However, no statistically significant changes were detected in the global age-standardized death or YLL rates. The burden of PAD and its temporal trends varied significantly by location, gender, age group, and social-demographic status. Among all potentially modifiable risk factors, age-standardized PAD deaths worldwide were primarily attributable to high fasting plasma glucose, followed by high systolic blood pressure, tobacco, kidney dysfunction, diet high in sodium, and lead exposure.

**Conclusion:**

PAD remained a serious public health problem worldwide. More strategies aimed at implementing cost-effective interventions and addressing modifiable risk factors should be carried out, especially in regions with high or increasing burden.

## Introduction

Peripheral artery disease (PAD) is characterized by atherosclerosis of the peripheral arteries which results in insufficient blood flow to the affected limb and subsequent ischemia. Most patients with PAD are asymptomatic, but many have experienced experience classic intermittent claudication, critical limb ischemia, occasionally acute limb ischemia, and the associated adverse cardiovascular events (1–3). PAD is the third common manifestation of atherosclerosis, following coronary artery disease and stroke (4). Despite its implications for public health and comorbidities, PAD remains underdiagnosed and undertreated.

Up-to-date epidemiological information is essential in public health policy making. As of 2010, ~202 million people worldwide lived with PAD, almost 70% of them living in low-income or middle-income countries (4). With the growth of the population, there were 236.62 million people aged 25 years and older living with PAD worldwide in 2015 (1). Patients with PAD often have concomitant cardiovascular disease (5), and the morbidity and mortality associated with PAD are found to be equal to or higher than those associated with coronary heart disease (6). Additionally, PAD not only imposes a huge economic burden on the health system (7), but also results in a significant indirect cost through loss of productivity.

The Global Burden of Disease study (GBD), an ongoing effort to estimate the global epidemiological trends of 369 diseases and injuries, provides a unique opportunity to understand the landscape of PAD. Sampson et al. reported the global landscape of PAD mortality using the data derived from the GBD study 2010 (8), while national-level estimates were not provided in their study. Another study utilizing GBD study 2017 only reported the PAD burden for the European Union (15+) countries (9). To the best of our knowledge, no updated study has provided detailed estimates for the burden of PAD after that. In the current study, we aimed to provide comprehensive and comparable analysis of PAD burden and attributable risk factors at the global, regional, and national levels from 1990 to 2019 by age, sex, Socio-demographic Index (SDI), based on the most recent GBD study 2019.

## Materials and Methods

### Overview

GBD 2019, conducted by Institute of Health Metrics and Evaluation (IHME), was designed to provide a systematic estimation of health loss due to diseases and injuries across 204 countries and territories, seven super-regions and 21 regions from 1990 to 2019 (10). And assessments for 369 diseases and injuries, 286 causes of death and 87 risk factors were reported in GBD 2019 (10–12). General methods of GBD 2019 and its main changes compared with previous years have been previously presented in GBD 2019 papers (10–12). In the present study, data for the burden of PAD were available on the Global Health Data Exchange (website: http://ghdx.healthdata.org/gbd-results-tool). The informed consent was waived because GBD 2019 used de-identified and aggregated data.

### Definitions

PAD was defined as having an ankle-brachial index <0.9, and intermittent claudication was defined clinically. All cardiovascular diseases coded as 440.2, 440.4 and 443.0–443.9 in the 9th revision of the International Classification of Diseases and Injuries (ICD-9) or I70.2–I70.8 and I73–I73.9 in the ICD-10 were recognized as PAD. Years lived with disability (YLDs) were calculated as the prevalence of each severity level and the corresponding disability weight. Disability weight reflects the severity of the disease on a scale from 0 (full health) to 1 (death). Years of life lost (YLLs) due to PAD were calculated using the number of deaths and a standard life expectancy according to age. Detailed descriptions of the estimation process are presented in the Text S1.

SDI was used to group countries with similar development status. SDI is composed of lag distributed income per capita, mean education in the population over age 15, and total fertility rate under 25 years, with a value range of 0 (worst) to 1 (best) (10–12). We divided 204 countries and territories into five groups according to SDI quintiles, including low, low-middle, middle, high-middle, and high. In addition, these countries were also divided into 21 regions based on epidemiological similarity and geographical proximity.

A comparative risk assessment approach was used to estimate the proportion of PAD attributable to 84 environmental, occupational, metabolic, and behavioral risk factors. Detailed process for quantifying the percentage contributions of different risk factors to disease burden has been published previously (11, 13). Risk factors were selected based on the following three criteria: sufficient evidence of causation with PAD, availability of exposure data, and potential for modification. Six potentially modifiable risk factors were considered to be associated with PAD deaths in our study, including diet high in sodium, high fasting plasma glucose, high systolic blood pressure, kidney dysfunction, lead exposure, and tobacco. Details about definitions of these risk factors and methods used in estimating exposure levels and associated PAD deaths are also presented in the Text S1.

### Statistical Analysis

Prevalence, incidence, death, YLDs, and YLLs were the main parameters evaluating the burden of PAD. Counts and age-standardized rates per 100,000 people, with 95% uncertainty intervals (UIs) were calculated to quantify the burden of PAD across gender, age group, year, location, and SDI. To avoid the interferences of population change and age distribution difference, age-standardized rates were also used and calculated by direct standardization to the global age structure. The population attributable fractions (PAFs) were calculated to quantify the percentage contributions of risk factors to age-standardized PAD deaths. The 95% UIs were determined by the 2.5 and 97.5 centile values of 1,000 draw-level estimates after ordering them from the smallest to the largest. A 95% UIs, excluding 0, was considered to be statistically significant. Besides, we analyzed the correlation between SDI and age-standardized rates by Pearson's correlation test model. All statistics were based on the R program (Version 3.6.1).

## Results

In 2019, PAD affected 113,443,017 (95% UI 99,158,208 to 128,415,296) people worldwide, increasing by 72.5% (95% UI 70.2% to 74.7%) compared to 1990 (Supplementary Table S1). There were 10,504,092(9,162,529 to 11,999,888) incident cases of PAD in 2019, which increased by 71.5% (95% UI 69.6% to 73.3%) between 1990 and 2019 (Supplementary Table S1). Despite the increases in absolute numbers, the age-standardized prevalence and incidence rates of PAD from 1990 to 2019 decreased by −21.7% (−95% UI 22.8% to −20.5%) and −18.9% (95% UI −19.8% to −18.0%), respectively, with the greatest decrease occurring in the high SDI countries (Table 1). In 2019, both the age-standardized prevalence and incidence rates were significantly higher in higher SDI countries (Table 1).

**Table 1:** Age-standardized prevalence, incidence, death, YLD, and YLL rates of peripheral artery disease in 2019, and their percentage changes from 1990 to 2019, by sex, SDI quintile, and GBD region.

	**Prevalence**		**Incidence**		**Deaths**		**YLDs**		**YLLs**	
	**Age-**	**Percentage**	**Age-**	**Percentage**	**Age-**	**Percentage**	**Age-**	**Percentage**	**Age-**	**Percentage**
	**standardized**	**change in age-**	**standardized**	**change in age-**	**standardized**	**change in age-**	**standardized**	**change in age-**	**standardized**	**change in age-**
	**rate per**	**standardized**	**rate per**	**standardized**	**rate per**	**standardized**	**rate per**	**standardized**	**rate per**	**standardized**
	**100,000**	**rates**	**100,000**	**rates**	**100,000**	**rates**	**100,000**	**rates**	**100,000**	**rates**
	**people**		**people**		**people**		**people**		**people**	
Global	1401.8 (1228.5–1589.4)	−21.7% (−22.8%−20.5%)	127.1 (111.3–145.4)	−18.9% (−19.8%−18.0%)	1.0 (0.6–1.7)	−2.5% (−21.9%−9.8%)	6.3 (3.0–11.3)	−26.5% (−27.7%−25.2%)	13.3 (7.7–22.6)	−4.2% (−22.3%−8.8%)
**Sex**										
Male	1008.31 (881.44–1143.68)	−21.70% (−22.90%−20.40%)	96.18 (83.93–109.99)	−18.90% (−19.80%−17.90%)	1.23 (0.56–2.63)	−1.60% (−18.70%−15.30%)	4.60 (2.17–8.20)	−27.80% (−29.10%−26.40%)	17.23 (8.27–35.75)	−4.20% (−21.10%−13.70%)
Female	1735.06 (1519.05–1964.03)	−20.40% (−21.60%−19.20%)	154.91 (135.53–177.03)	−18.10% (−19.00%−17.10%)	0.83 (0.36–1.70)	−4.60% (−36.70%−9.30%)	7.66 (3.61–13.80)	−24.60% (−26.00%−23.00%)	9.85 (4.55–19.90)	−5.90% (−37.00%−10.50%)
**SDI quintiles**										
Low SDI	938.6 (815.0–1074.6)	0.5% (−0.4%−1.5%)	94.1 (81.5–107.4)	−2.9% (−3.8%−1.9%)	0.7 (0.4–1.0)	27.0% (−2.4%−66.4%)	5.3 (2.5–9.5)	−7.3% (−9.5%−4.4%)	11.5 (6.5–15.6)	20.9% (−7.7%−58.0%)
Low-middle SDI	1042.2 (905.4–1190.1)	−1.1% (−2.2%−0.1%)	103.2 (89.5–118.2)	−2.1% (−3.0%−1.1%)	0.4 (0.3–0.5)	55.4% (21.0%−82.4%)	5.5 (2.6–9.9)	−12.2% (−14.5%−9.1%)	5.2 (4.0–6.6)	53.5% (19.3%−81.5%)
Middle SDI	1264.8 (1098.3–1446.0)	1.1% (0.1%−2.0%)	114.7 (99.6–131.5)	−3.4% (−4.3%−2.4%)	0.4 (0.3–0.5)	29.3% (4.3%−49.8%)	6.3 (2.9–11.3)	−13.9% (−16.9%−10.0%)	4.9 (3.8–6.8)	26.5% (3.1%−46.9%)
High-middle SDI	1506.1 (1314.6–1711.8)	−15.4% (−16.4%−14.2%)	133.6 (116.2–153.1)	−15.0% (−16.0%−14.1%)	1.4 (0.7–2.4)	−5.3% (−25.2%−11.4%)	6.7 (3.1–12.0)	−23.9% (−25.4%−22.3%)	19.0 (10.0–34.7)	−8.4% (−30.2%−8.4%)
High SDI	1794.0 (1585.1–2006.1)	−34.0% (−35.8%−32.0%)	157.7 (139.5–178.5)	−32.1% (−33.9%−30.2%)	1.5 (0.7–2.8)	7.3% (−17.9%−22.0%)	6.7 (3.1–12.1)	−38.1% (−40.0%−36.1%)	20.6 (10.2–38.4)	7.5% (−16.7%−21.1%)
**GBD region**										
Central Asia	1218.1 (1060.0–1392.9)	0.8% (−1.1%−2.8%)	118.2 (102.5–135.6)	−0.6% (−2.5%−1.4%)	0.2 (0.1–0.3)	71.8% (28.2%−116.7%)	5.8 (2.8–10.5)	−6.9% (−9.9%−3.2%)	3.0 (1.5–5.4)	61.8% (19.3%−94.9%)
Central Europe	1313.0 (1140.8–1498.9)	−10.5% (−11.6%−9.5%)	124.1 (107.3–142.0)	−10.7% (−11.7%−9.7%)	1.8 (0.9–3.3)	25.5% (−15.0%−73.7%)	5.6 (2.7–10.2)	−20.5% (−22.1%−18.7%)	27.3 (13.2–51.5)	20.5% (−16.2%−58.1%)
Eastern Europe	1690.4 (1467.3–1936.0)	−1.9% (−3.3%−0.5%)	153.7 (133.6–175.5)	−1.9% (−3.3%−0.6%)	3.5 (1.7–6.7)	31.6% (−7.4%−63.5%)	7.5 (3.6–13.7)	−10.4% (−12.7%−7.7%)	56.1 (26.2–109.4)	33.1% (−9.7%−67.0%)
Australasia	1254.3 (1086.1–1424.4)	−37.0% (−39.3%−34.4%)	113.3 (98.3–130.1)	−35.7% (−37.9%−33.1%)	2.5 (1.2–4.7)	14.0% (−40.3%−50.8%)	4.5 (2.1–8.1)	−41.1% (−44.7%−37.3%)	28.4 (13.6–54.6)	9.2% (−45.7%−43.6%)
High-income Asia Pacific	1303.9 (1132.7–1480.4)	−41.6% (−42.3%−40.8%)	115.5 (100.5–131.8)	−39.3% (−40.2%−38.4%)	0.2 (0.1–0.4)	−7.6% (−38.9%−37.3%)	4.8 (2.3–8.8)	−46.5% (−47.6%−45.4%)	2.7 (1.5–4.6)	−14.6% (−41.6%−19.7%)
High-income North America	2214.3 (1986.7–2433.8)	−29.2% (−33.7%−23.7%)	193.5 (173.7–215.6)	−28.8% (−33.1%−23.8%)	2.2 (1.1–4.2)	30.9% (5.6%−50.7%)	7.7 (3.6–13.7)	−30.4% (−35.2%−24.8%)	31.6 (15.3–59.8)	25.3% (2.2%−43.5%)
Southern Latin America	1568.2 (1344.5–1775.4)	−25.7% (−28.1%−23.0%)	145.0 (125.3–166.7)	−26.1% (−28.5%−23.2%)	0.6 (0.3–1.2)	54.9% (14.2%−89.0%)	6.6 (3.1–11.9)	−33.0% (−36.5%−29.0%)	8.8 (4.2–16.7)	50.4% (10.1%−82.9%)
Western Europe	1902.5 (1659.4–2145.4)	−34.2% (−35.1%−33.3%)	165.2 (143.3–189.1)	−32.5% (−33.6%−31.5%)	1.9 (0.9–3.5)	11.0% (−20.5%−32.6%)	7.3 (3.4–13.2)	−40.4% (−41.8%−39.0%)	23.9 (11.4–45.4)	7.0% (−23.7%−27.6%)
Andean Latin America	828.7 (715.4–951.2)	−3.8% (−6.9%−0.8%)	84.5 (73.1–96.9)	−4.7% (−7.7%−2.0%)	0.1 (0.1–0.2)	19.6% (−8.6%−58.1%)	4.1 (1.9–7.4)	−18.0% (−22.3%−13.3%)	1.8 (1.3–2.2)	18.8% (−8.9%−54.6%)
Caribbean	985.7 (848.4–1129.5)	−5.9% (−7.8%−3.8%)	99.3 (85.8–114.2)	−6.3% (−8.0%−4.1%)	1.9 (1.0–3.4)	20.8% (−17.0%−48.0%)	4.7 (2.3–8.5)	−13.5% (−16.9%−10.3%)	28.1 (14.9–49.7)	19.1% (−17.7%−47.1%)
Central Latin America	1038.9 (897.5–1190.4)	−15.4% (−16.6%−14.2%)	104.5 (90.2–120.4)	−14.6% (−15.9%−13.4%)	0.6 (0.3–1.2)	6.5% (−36.6%−46.1%)	5.0 (2.4–9.1)	−24.4% (−26.6%−21.4%)	7.9 (3.8–15.8)	5.5% (−37.9%−45.9%)
Tropical Latin America	941.6 (817.7–1072.8)	−20.7% (−21.9%−19.6%)	95.0 (82.1–108.8)	−19.8% (−21.0%−18.6%)	1.2 (0.6–2.3)	15.5% (−16.7%−52.1%)	4.6 (2.2–8.4)	−28.5% (−31.0%−25.3%)	18.7 (8.6–35.3)	11.2% (−18.9%−46.7%)
North Africa and Middle East	1115.6 (966.1–1276.4)	−1.3% (−2.6%−0.1%)	109.7 (95.0–125.8)	−1.6% (−2.9%−0.3%)	0.4 (0.3–0.6)	28.7% (−2.3%−66.2%)	5.2 (2.5–9.4)	−15.3% (−17.7%−11.7%)	6.0 (4.9–7.8)	18.2% (−12.7%−51.6%)
South Asia	917.3 (797.7–1047.2)	−0.1% (−1.1%−0.8%)	93.1 (80.5–106.5)	−0.6% (−1.6%−0.3%)	0.3 (0.2–0.4)	71.1% (11.6%−114.9%)	4.8 (2.3–s8.4)	−10.8% (−13.2%–−7.4%)	4.2 (3.1–5.3)	67.9% (8.9%−113.3%)
East Asia	1426.7 (1238.8–1627.0)	6.8% (5.5%−8.1%)	125.6 (109.3–143.5)	3.2% (2.0%−4.4%)	0.1 (0.1–0.2)	21.1% (−13.7%−51.8%)	6.9 (3.2–12.5)	−13.4% (−17.8%–−8.4%)	1.9 (1.6–2.4)	15.3% (−18.4%−46.5%)
Oceania	1450.5 (1258.7–1653.5)	9.3% (6.6%−12.9%)	139.2 (120.9–159.1)	8.9% (6.0%−12.1%)	0.2 (0.2–0.3)	47.8% (10.3%−92.8%)	8.5 (4.0–15.5)	4.7% (−0.4%−10.4%)	3.1 (2.4–4.6)	49.0% (13.7%−95.2%)
Southeast Asia	1511.7 (1314.0–1725.4)	4.2% (2.8%−5.7%)	140.6 (122.2–161.0)	2.1% (0.8%−3.3%)	0.2 (0.2–0.2)	52.9% (23.5%−80.5%)	8.1 (3.8–14.7)	−6.4% (−9.4%–−2.6%)	3.0 (2.3–3.7)	52.7% (22.4%−82.0%)
Central Sub–Saharan Africa	1073.5 (924.1–1224.6)	−0.6% (−3.9%−2.8%)	111.4 (96.6–127.6)	0.1% (−3.1%−3.5%)	2.2 (1.1–3.5)	−6.9% (−33.8%−57.9%)	6.3 (3.0–11.4)	−6.0% (−11.3%–−0.3%)	36.8 (18.9–58.6)	−6.6% (−34.8%−57.1%)
Eastern Sub–Saharan Africa	939.1 (813.8–1076.6)	2.4% (1.2%−3.8%)	97.8 (84.4–111.9)	2.0% (0.8%−3.4%)	1.4 (0.7–1.9)	40.7% (8.9%−77.3%)	5.4 (2.5–9.6)	−4.7% (−7.3%–−1.5%)	22.2 (12.0–29.5)	35.9% (2.1%−73.0%)
Southern Sub–Saharan Africa	1307.5 (1135.5–1490.7)	−13.5% (−14.9%–−12.0%)	126.2 (109.5–144.8)	−11.4% (−12.9%–−9.8%)	2.0 (1.6–2.2)	49.8% (27.5%−70.6%)	7.0 (3.3–12.5)	−18.4% (−20.8%–−15.5%)	34.8 (27.5–39.7)	44.3% (21.2%−67.0%)
Western Sub–Saharan Africa	902.7 (783.4–1036.4)	4.3% (3.5%−5.1%)	93.5 (81.0–107.2)	4.3% (3.5%−5.0%)	0.3 (0.2–0.4)	38.3% (−8.9%−94.0%)	5.1 (2.4–9.2)	−2.5% (−4.6%−0.0%)	5.4 (2.9–7.3)	33.3% (−13.6%−89.7%)

*Data in parentheses are 95% uncertainty intervals*.

*GBD, Global Burden of Disease, Injuries, and Risk Factors Study; SDI, socio–demographic index; YLDs, years lived with disability; YLLs, years of life lost*.

PAD contributed to 74,063 (95% UI 41,183 to 128,164) deaths globally in 2019, having an increase of 145.5% (95% UI 96.5% to 176.2%) between 1990 and 2019 (Supplementary Table S1). Additionally, PAD was responsible for 500,893(95% UI 234,625 to 898,104) YLDs and 1,035,487(95% UI 604,347 to 1,778,564) YLLs globally in 2019, with an increase of 65.4% (95% UI 62.0% to 69.4%) and 119.1% (95% UI 78.3% to 150.4%) compared to 1990 (Supplementary Table S1). The global age-standardized YLD rate of PAD decreased by −26.5% (95% UI −27.7% to −25.2%) over the time period studied (Table 1). However, no significant decline in the age-standardized death and YLL rates was detected (Table 1). As for the age-standardized YLD rate, the most significant decrease was detected in high SDI countries, followed by countries in the high-middle SDI (Table 1).

**Figure 1:**
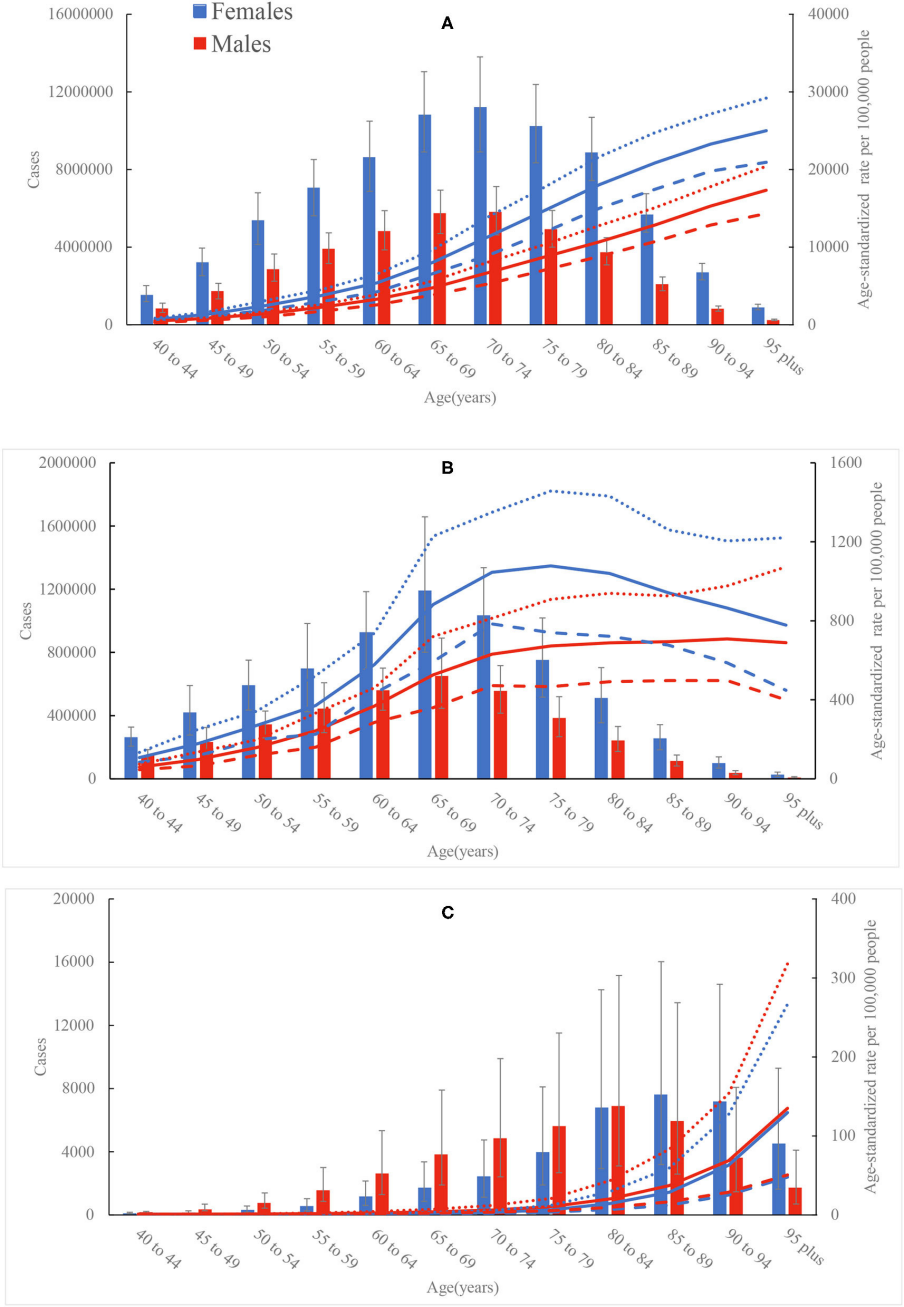
Age-specific numbers and rates of prevalent cases **(A)**, incident cases **(B)**, and deaths **(C)** of peripheral artery disease by sex, 2019. Dotted and dashed lines indicate 95% upper and lower uncertainty intervals, respectively.

Globally, the numbers and age-standardized rates of prevalent cases, incident cases, and YLDs of PAD were all higher in females than in males across all years (Supplementary Table S2, Supplementary Figure S1). Conversely, the age-standardized rates of deaths, and YLLs of PAD were lower in females than in males across all years (Supplementary Table S2, Supplementary Figures S1, S2). By age group, the numbers and age-standardized rates of prevalence, incidence, and YLDs of PAD were higher in females than in males across all ages in 2019 (Figure 1). What's more, the numbers of deaths and YLLs of PAD were higher in females than in males after the ages of 85 to 89 years, while the age-standardized death and YLL rates were higher in males than in females across all ages (Figure 1, Supplementary Figure S3). Additionally, PAD-related age-specific prevalence, death, YLD, and YLL rates tended to increase with age in both sexes (Figure 1, Supplementary Figure S3). The age-specific incidence rate peaked at the ages of 90 to 94 years in males, whereas the peak in females was observed at the ages of 75 to 79 years (Figure 1).

**Figure 2:**
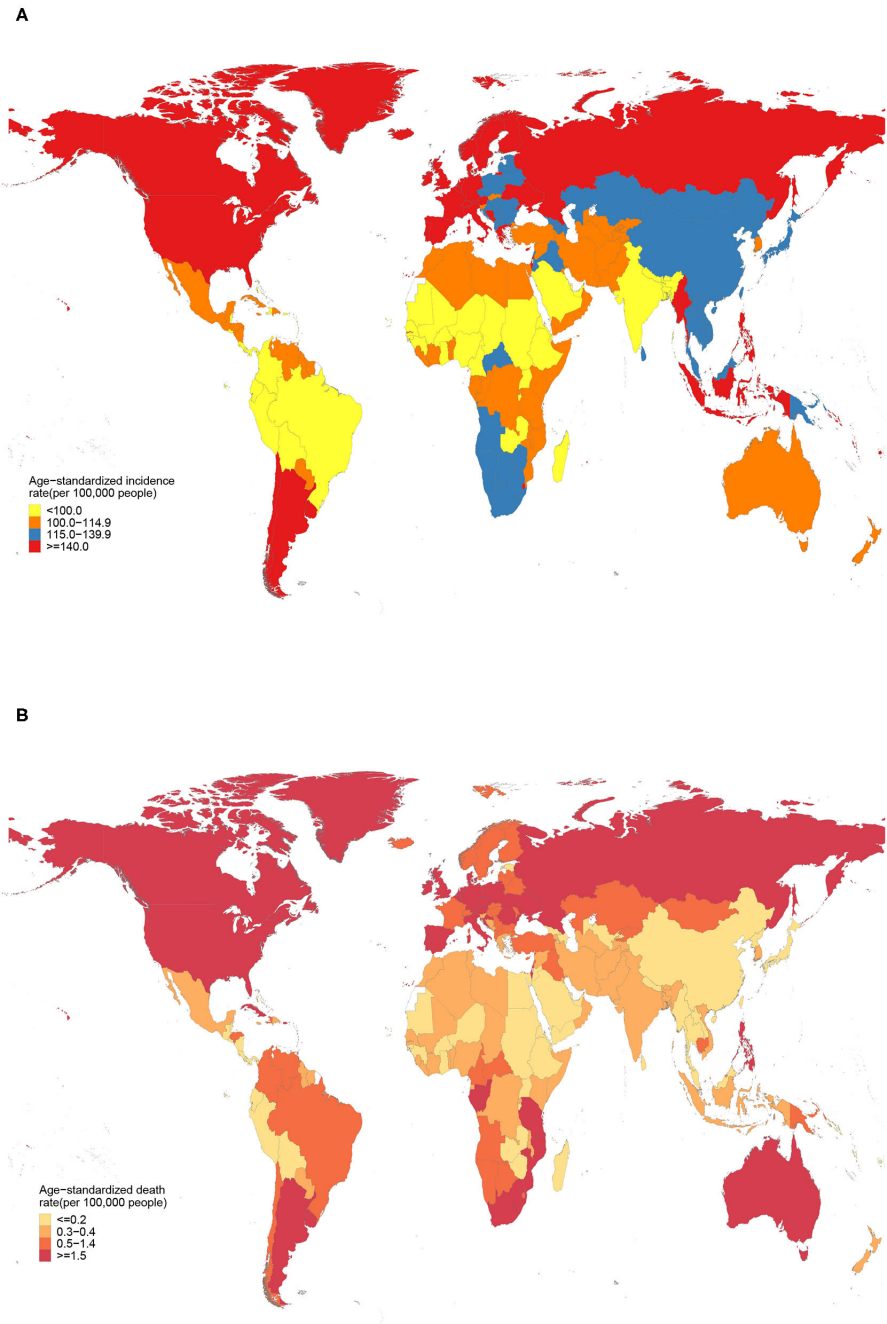
Age-standardized incidence **(A)** and death **(B)** rates of peripheral artery disease across 204 countries and territories in 2019.

Across 21 GBD regions, Andean Latin America had the lowest age-standardized incidence [84.5 (95% UI 73.1 to 96.9) per 100,000 people] and death [0.1 (95% UI 0.1 to 0.2) per 100,000 people] rates of PAD in 2019; whereas High-income North America had the highest age-standardized incidence rate [193.5 (95% UI 173.7 to 215.6) per 100,000 people] and Eastern Europe had the highest age-standardized death rate [3.5 (95% UI 1.7 to 6.7) per 100,000 people] (Table 1). Among all countries, the lowest age-standardized incidence and death rates were detected in Peru [81.3 (95% UI 69.9 to 93.6) per 100,000 people] and Thailand [0.1 (95% UI 0.1 to 0.1) per 100,000 people], respectively; whereas the highest age-standardized incidence and death rates were found in Denmark [224.9 (95% UI 194.6 to 257.5) per 100,000 people] and Barbados [7.1 (95% UI 3.4 to 13.2) per 100,000 people] (Figure 2, Supplementary Table S3). The variations in other PAD burden metrics (prevalence, YLDs, and YLLs) by locations in 2019 were summarized in Supplementary Tables S1, S3.

**Figure 3:**
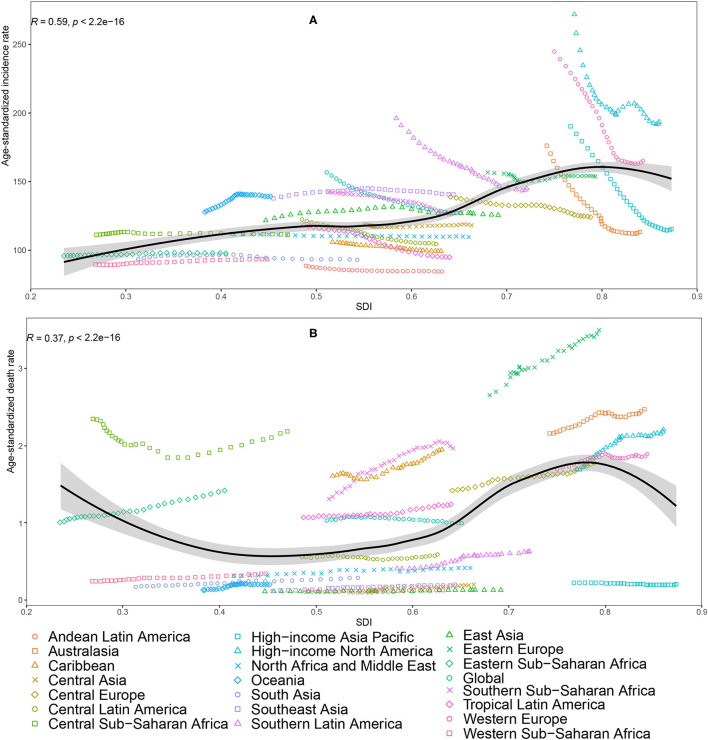
Age-standardized incidence rate **(A)** and age-standardized death rate **(B)** of peripheral artery disease for 21 Global Burden of Disease (GBD) regions by Socio-Demographic Index (SDI), 1990–2019; expected values based on SDI and disease rates in all locations are shown as the black line. Thirty points are plotted for each GBD region and show observed rate from 1990 to 2019 for that region. The R indices and *P*-value were derived from Pearson correlation analysis.

From 1990 to 2019, the age-standardized incidence rate of PAD sharply decreased in regions with relatively high-income, with the greatest decrease seen in High-income Asia Pacific [−39.3% (95% UI −40.2 to −38.4)], followed by Australasia, Western Europe, and High-income North America (Table 1, Figure 3). Canada, Japan, and United Kingdom had the greatest decrease in age-standardized incidence rate (Supplementary Table S3). A decline trend of age-standardized death rate was seen in High-income Asia Pacific [−7.6% (95% UI −38.9% to 37.3%)], which did not reach statistical significance (Table 1, Figure 3). The Republic of Korea was the only country that showed a decrease in age-standardized death rate of PAD [−33.8% (95% UI −53.1% to −13.3%)] (Supplementary Table S3). Notably, the largest increases of age-standardized incidence and death rates were seen in Oceania [8.9% (95% UI 6.0% to 12.1%)] and Central Asia [71.8% (95% UI 28.2% to 116.7%)] (Table 1, Figure 3). During the study period, 36.2% of the increase in global number of incident cases occurred in China, with China having the biggest increase in number of incident cases compared to other countries (Supplementary Table S1).

**Figure 4:**
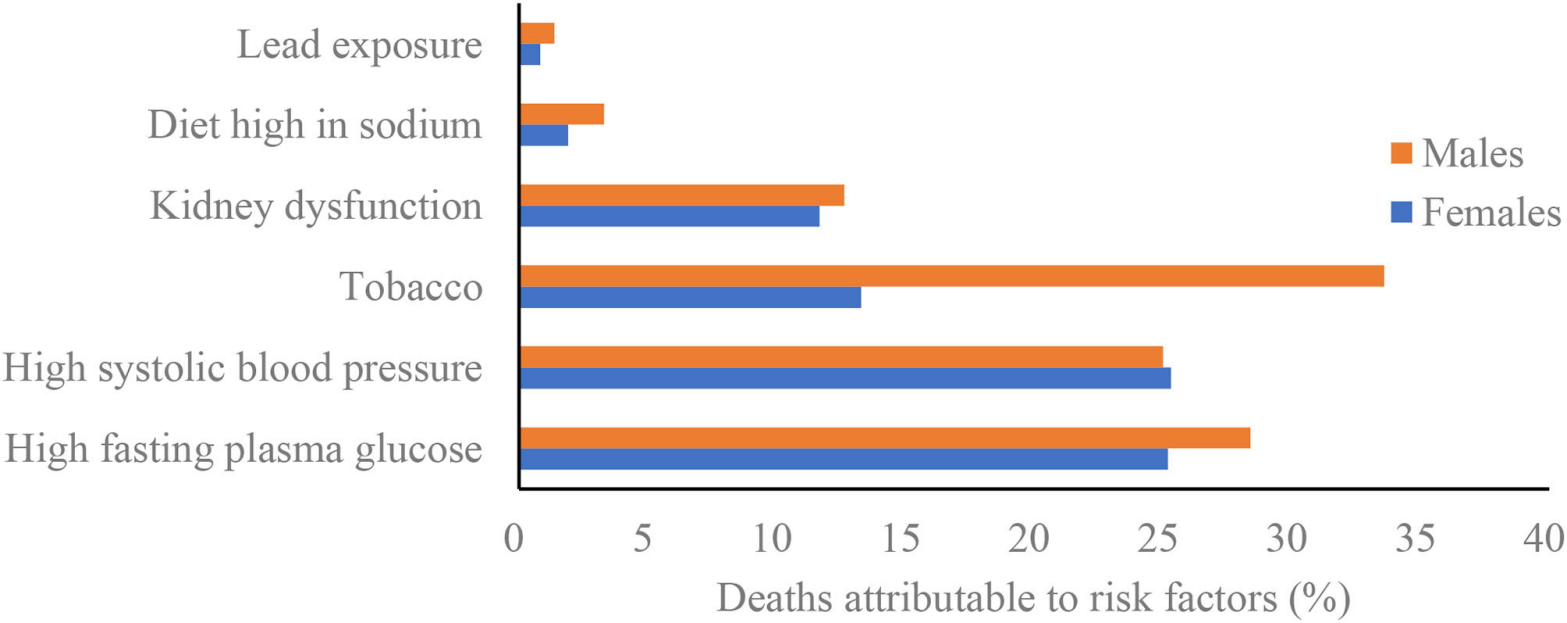
Percentage contributions of major risk factors to peripheral artery disease age-standardized deaths by sex, 2019.

Among all risk factors quantified in GBD study 2019, the global age-standardized death of PAD was primarily attributable to high fasting plasma glucose [26.8% (95% UI 22.8% to 31.0%)], followed by high systolic blood pressure [25.3% (95% UI 18.1% to 34.0%)], tobacco [24.2% (95% UI 17.5% to 31.0%)], kidney dysfunction [12.3% (95% UI 7.3% to 16.9%)], diet high in sodium [2.6% (95% UI 0.5% to 7.1%)], and lead exposure [1.1% (95% UI 0.4% to 1.9%)]. In addition, Figure 4, Supplementary Table S4 present the percentage contributions of the above risk factors to the age-standardized death of PAD in 2019 by sex, SDI, and GBD regions. Particularly, the percentage contribution of tobacco use to age-standardized death of PAD was much larger in males than in females. By SDI quintiles, the percentage contribution of high fasting plasma glucose was largest in countries in the high SDI countries in both sexes, whereas the percentage contribution of lead exposure was larger in countries in the low-middle and low SDI countries in both sexes. Globally, from 1990 to 2019, there were upward trends in the percentage contributions of high fasting plasma glucose, kidney dysfunction and lead exposure in both sexes, whereas downward trends were observed for all other risk factors in both sexes.

## Discussion

The present study has evaluated the global PAD burden from 1990 to 2019, with attention paid to the comparisons across different age groups, sex, locations and attributable risk factors. In 2019, 113,443,017 individuals lived with PAD and 10,504,092 new PAD cases occurred globally, contributing to 74,063 deaths, 500,893 YLDs and 1,035,487 YLLs. In addition, compared with GBD 2010 (8), we also performed attributable risk factors analyses in our study, and revealed that the top three potential modifiable risk factors for PAD deaths in 2019 were high fasting plasma glucose, high systolic blood pressure and tobacco use.

The estimated numbers of prevalent cases of PAD based on GBD estimates were lower than previous study (4). The discrepancies are partly due to differences in data sources and estimation methods. Fowkes et al. inputted the datasets which were extracted from the published studies on prevalence of PAD into a model to estimate the age-specific prevalence data (4). However, most of the included studies were from high-income countries where the prevalence rate of PAD was high. Additionally, a value prior of 0 was set for proportion for ages 0 to 40 in GBD modeling strategy (10). On the contrary, patients aged 25 to 40 years were also recruited in the study by Fowkes et al. (4).

In agreement with previous studies (1, 4, 6, 8, 14, 15), our study found that PAD remained a major global public health challenge. Patients with PAD have been determined to be at very high risk of both major adverse cardiovascular events and major adverse limb events (6, 16–18), but the disease is frequently underdiagnosed and undertreated. The absolute numbers of YLDs increased to 500,893 worldwide in 2019, while the age-standardized YLD rate during study period tended to decline globally, especially in high SDI countries, suggesting that advancements in PAD management could prevent complications and improve the quality of life for patients (19). Significant gains have been made in evidence-based medical therapies for PAD in recent years including the addition of PCSK9 inhibitors and the use of low-dose rivaroxaban (19). Current professional society guidelines recommend an endovascular-first revascularization strategy in most symptomatic PAD patients with critical limb-threatening ischemia. Endovascular treatment has increased each year in the United States (20). Endovascular treatment of PAD has evolved and expanded rapidly over the last 20 years. New technologies have increased the diversity of devices available and have made it possible to approach even the most challenging and high-risk lesions using endovascular techniques (21). Access to endovascular treatment is lacking or absent in some countries, despite it being an essential treatment of PAD. Moreover, more females than males were affected by PAD worldwide, as the age-standardized rates of prevalence, incidence, and YLDs were higher in females than in males after the age of 40 years. Contrary to the previous opinion that PAD is mainly a disease of males (22), contemporary data suggests a similar or higher prevalence of PAD in females (23, 24). Females with PAD often present without symptoms and seek medical attention with more advanced disease and comorbid illness (23, 24), while males are more likely to undergo preventative and intervention strategies for their PAD than females (23). We found that males had a higher age-standardized rates of death and YLL than females, but this disparity was less prominent. Actually, there is limited recruitment of patients with PAD in clinical trials, especially females (25). Therefore, effective preventative and intervention strategies should be implemented in both sexes. Furthermore, the disease burden of PAD increased with age. Except for the age-standardized incidence rate, PAD-related age-specific prevalence, death, YLD, and YLL rates peaked in the elderly. PAD should be a focus of particular concern among the elderly population because this condition is usually indicative of cardiovascular and cerebrovascular diseases (26). Many elderly people do not walk far enough to experience symptoms of PAD, so the proportion of elderly patients with complaints of intermittent claudication is relatively low (27). The biological changes in elderly patients require more cautious treatment decisions (28), but they have been markedly underrepresented in clinical trials, as well as in secondary prevention and rehabilitation programs (28). Due to the acceleration of population growth and aging, more and better research should be conducted to find out the appropriate medical management of elderly with PAD.

The GBD 2019 data showed that the age-standardized prevalence and incidence rates have decreased during the period 1990 to 2019. However, this decline has not necessarily led to a lower burden of PAD on the health systems in high-risk countries. This is because changes in the population age structure and population growth have meant that numbers of prevalence and incidence of PAD have continued to increase in many regions. Contrasting with declined changes in age-standardized prevalence and incidence rates, the age-standardized death rates has not declined from 1990 to 2019. A recent study also found that PAD subjects still have a high mortality risk, and the magnitude is similar to data presented almost 20 years ago (18). Compared with the GBD 2010 analysis of PAD (8), most regions continued to experience a high PAD burden. PAD generally have a poor prognosis, with an increased risk of mortality, cardiovascular, and limb events and decreased quality of life. The 5-year mortality associated with chronic limb-threatening ischemia without amputation is estimated to be 55 to 65%, which exceeds the 5-year mortality of many malignancies (15). Furthermore, caring for PAD imposes a significant economic burden to the healthcare system (7, 15). The average annual expenditure per individual is $11,553 for patients with PAD vs. $4,219 for those without (29). The medical expenditure is particularly high in patients with PAD requiring major amputation (15). This expenditure, combined with lost wages, family care, and lost opportunity costs, increases the burden carried by patients with PAD. The managements of PAD are multifaceted, including lifestyle modifications, medical management, endovascular repair, or surgery. Although recent improvements in therapeutic approaches provide a significant opportunity for practitioners to detect and treat PAD in a timely and effective manner, our results showed that only Republic of Korea achieved a reduction in PAD mortality during the study period. PAD is asymptomatic in the early stage which makes detection and treatment difficult prior to the symptomatic progressed stages. The mortality risk is approximately doubled in symptomatic PAD patients compared with reference subjects and increase by severity of PAD stages (18). Evidences suggest that this is poorly managed in patients with PAD when compared with patients with known coronary disease (14, 30, 31). In fact, PAD treatment guidelines are still not fully implemented (19, 32). Interestingly, disparities in PAD management were also observed within the United States (33), which may be due to different exposures to risk factors and access to effective health care interventions. Both black and African-American patients experience higher rates of PAD as well as lower rates of access to preventative care (33). What's more, African-American and Native-American patients, and those of low socioeconomic status have the highest risk of amputation (33). These findings call for improved treatments to address the clinical, humanist are crucial to assess the best strategies for optimum treatment and prevention of this disease.

The regional age-standardized prevalence and incidence rates of PAD increased with increasing SDI. Higher incidence of PAD in high SDI countries could be due to the population age structure and to lifestyle choices that increase exposure to risk factors. Some of the risk factors for PAD are more prevalent in high SDI countries than in low ones (1). The age-standardized incidence rate of PAD is decreasing in high income regions, such as High-income Asia Pacific, Australasia, Western Europe, and High-income North America. A recent study examined the incidence of PAD between 1990 and 2017 in EU15+ countries, which have similarities in health expenditure and comparability (9). During the interval, there was a declining trend in the age-standardized incidence rate in this group of countries. Previous evidences suggest that the age-adjusted incidence of coronary heart disease has decreased over time in developed countries (34). Given the fact that the pathogenesis of PAD and coronary heart disease exist some similarities, the decline of age-standardized incidence rate of PAD do not implausible. During the study period, the age-standardized incidence rate increased in several regions, such as, Oceania, East Asia and Southeast Asia. It is worth noting that most of new cases occurred in these regions. In particular, approximately 36.2% of the increase in global number of incident cases occurred in China. Health systems in these countries are not sufficiently robust to handle an increased number of PAD. Therefore, primary prevention programs including multi-level approaches at the population and individual levels could be a key part of a coordinated response to the increased PAD burden.

In 2019, except for age and other non-modifiable risk factors, high fasting plasma glucose, high systolic blood pressure, and tobacco use remained the top three contributors to PAD deaths worldwide, which is in line with other studies (1, 4). We found that the PAFs of age-standardized deaths attributable to tobacco use decreased slightly from 1990 to 2019 in both sexes. Reductions in the proportion of PAD attributable to tobacco use are similar to the worldwide reductions in smoking rates. Globally, the age-standardized prevalence of smoking decreased by 27.5% for males and 37.7% for females between 1990 and 2019 (35). On the basis of a previous study, former smokers, in comparison with never smokers, had an odds ratio (OR) of 2.19, and current smokers, in comparison with never smokers, had an OR of 3.83, for risk of PAD (36). Meanwhile, compared with never smokers, current and former smokers with PAD had higher rates of death (37). High exposure to environmental tobacco also increased the risk of PAD among non-smokers (38). We found that the percentage contribution of tobacco use to age-standardized death of PAD was much larger in males than in females, which is consistent with the findings from Kumakura et al. (39). High systolic blood pressure was also identified as potential risk factors attributable to PAD deaths, which showed a decreasing trend of this risk factor globally, especially in the high SDI and high middle SDI countries. However, in some regions—for example, Western Sub-Saharan Africa—the age-standardized rate of deaths attributed to high systolic blood pressure increased between 1990 and 2019. Diet high in sodium has been shown to increase the risk of hypertension. Actually, daily intake of sodium is much higher than the recommended intakes in both hypertension and normal blood pressure (40). Unfortunately, there was no remarkable decline in the proportion of PAD attributable to diet high in sodium since 1990. Therefore, multiple efforts are still needed to create a heart-healthy diet combined with pharmacological treatment of hypertension. There were upward trends in the percentage contributions of high fasting plasma glucose, kidney dysfunction and lead exposure to PAD deaths in both sexes from 1990 to 2019. Perhaps the most concerning are high fasting plasma glucose and kidney dysfunction, since they have become the modern epidemics worldwide. There were large variations between countries in the percentage contributions of high fasting plasma glucose to PAD deaths, with notably high rates in high SDI countries. Kidney dysfunction is also strongly and independently associated with PAD (41). Kidney dysfunction is common, frequently unrecognized and often exists together with other conditions (such as cardiovascular disease and diabetes). Driven by the aging population, both high fasting plasma glucose and kidney dysfunction are emerging as a growing global problem. More effective preventive interventions are needed, and health systems should focus on new approaches that can reverse the trends. We observed that exposure to lead is also a contributor to PAD-related mortality, especially in low SDI countries. Lead exposure may promote atherosclerosis. It has been reported that lead exposure is associated with an increased prevalence of PAD in the general population (42). In light of the current spate of industrialization with increasing chemical utilization and chemical waste generation, regulatory and public health interventions must be developed and implemented to further protection against lead exposure.

GBD studies provide high-quality estimates of global disease burden, but there were still several limitations in our study. Firstly, the estimates for PAD burden were a combination of data, which largely dependent on the quality and quantity of data used during modeling. The variations in epidemiological data may be due to detection biases as well as changes in screening protocols. Both the paucity of rigorous epidemiological data and the level of missing and unusable data for some countries could affect the estimates of PAD burden. Secondly, although ICD codes are widely accepted for death certification and GBD 2019 has made substantial efforts to improve data quality, the misclassification of PAD death cannot be fully avoided because PAD is often attended by coexisting conditions, such as myocardial infarction or stroke. Thirdly, the socio-cultural and ethnic differences are frequently associated with health behaviors and risk factors that affect the global burden of PAD, but these differences were not captured during modeling. Finally, we were not able to account for some risk factors like hyperlipemia, as they were omitted in the GBD database.

In conclusion, PAD remained a serious public health problem worldwide, with considerable heterogeneity in the epidemiologic patterns across sex, age, SDI, region, and country. The countries with high SDI had the highest incidence rate but had the largest decrease in incidence rate between 1990 and 2019. Although we have made great achievements in PAD prevention and treatment, especially in regions with relatively high SDI, PAD related mortality did not decline during study period. More strategies aimed at implementing cost-effective interventions and addressing modifiable risk factors should be carried out, especially in regions with high or increasing burden.

## Data Availability Statement

The datasets presented in this study can be found in online repositories. The names of the repository/repositories and accession number(s) can be found in the article/Supplementary Material.

## Author Contributions

JL and SX contributed to conception and design of the study. JL and YC performed the statistical analysis. JL, NJ, and ZL wrote the first draft of the manuscript. SX reviewed and edited the manuscript. All authors contributed to the article and approved the final manuscript.

## Conflict of Interest

The authors declare that the research was conducted in the absence of any commercial or financial relationships that could be construed as a potential conflict of interest.

## Publisher's Note

All claims expressed in this article are solely those of the authors and do not necessarily represent those of their affiliated organizations, or those of the publisher, the editors and the reviewers. Any product that may be evaluated in this article, or claim that may be made by its manufacturer, is not guaranteed or endorsed by the publisher.
