# Supplementary material for: Burden of Peripheral Artery Disease and Its Attributable Risk Factors in 204 Countries and Territories From 1990 to 2019
Source: Front Cardiovasc Med. 2022 Apr 12;9:868370. doi: 10.3389/fcvm.2022.868370 (PMC9039520; doi:10.3389/fcvm.2022.868370)
Supplement: Supplementary file 1 [file Table_1.pdf]

Table S1. Number of peripheral artery disease prevalent cases, incident cases, deaths, YLDs, and YLLs in 2019, and their percentage changes from 1990 to 2019, by SDI quintile, and GBD region

	Prevalence		Incidence		Deaths		YLDs		YLLs	
	Number cases	Percentage change in number cases	Number cases	Percentage change in number cases	Number cases	Percentage change in number cases	Number cases	Percentage change in number cases	Number cases	Percentage change in number cases
**Global**	113443017 (99158208-128415296)	72.5% (70.2%-74.7%)	10504092 (9162529-11999888)	71.5% (69.6%-73.3%)	74063 (41183-128164)	145.5% (96.5%-176.2%)	500893 (234625-898104)	65.4% (62.0%-69.4%)	1035487 (604347-1778564)	119.1% (78.3%-150.4%)
**SDI quintiles**										
**Low SDI**	4354605 (3744362-5006216)	126.3% (123.5%-129.3%)	470118 (404625-538077)	114.9% (112.0%-118.1%)	2684 (1500-3641)	186.5% (116.2%-283.5%)	21879 (10260-39153)	109.9% (103.3%-118.0%)	51064 (29678-69282)	164.1% (98.2%-250.9%)
**Low-middle SDI**	13410114 (11591984-15328969)	139.1% (134.9%-143.5%)	1398309 (1206028-1602467)	128.6% (124.6%-132.9%)	3721 (2773-4762)	328.4% (228.5%-408.7%)	66031 (30925-118081)	123.0% (114.6%-133.2%)	62647 (48113-78309)	283.6% (194.3%-357.1%)
**Middle SDI**	30576763 (26400708-35052196)	160.8% (157.7%-164.1%)	2915099 (2520526-3342399)	141.7% (138.7%-144.6%)	6643 (5028-9304)	273.5% (204.2%-334.1%)	141511 (65058-254571)	130.3% (120.9%-142.1%)	109239 (84336-147880)	235.6% (174.2%-291.1%)
**High-middle SDI**	30755470 (26773011-34985543)	70.8% (68.8%-72.9%)	2745997 (2383531-3147380)	64.3% (62.5%-66.3%)	26243 (13528-47034)	117.7% (69.3%-160.4%)	135870 (63778-244664)	58.7% (54.0%-64.6%)	384804 (201007-702372)	90.3% (43.2%-126.5%)
**High SDI**	34291798	20.4%	2855740	18.2% (14.7%-21.8%)	34713	139.2%	135353	17.1% (12.4%-22.1%)	426939	111.4% (64.2%-137.7%)

	(30198669-38329603)	(16.7%-24.3%)	(2518228-3241195)		(16832-63581)	(82.9%-172.7%)	(63670-244469)		(208630-793277)	
**GBD region**										
**Central Asia**	809162 (698260-926301)	53.6% (49.3%-57.9%)	85439 (73678-98428)	59.6% (54.7%-64.5%)	101 (52-181)	123.8% (63.4%-175.9%)	3421 (1601-6199)	32.4% (25.9%-39.2%)	1897 (971-3534)	138.1% (70.6%-190.8%)
**Central Europe**	2849874 (2451339-3276016)	35.4% (32.4%-38.8%)	262490 (225127-303309)	27.9% (24.4%-31.6%)	4004 (1934-7372)	116.3% (47.9%-194.7%)	12853 (6176-23498)	28.8% (23.9%-35.0%)	60156 (29168-113573)	85.8% (30.9%-148.6%)
**Eastern Europe**	5908656 (5117977-6767587)	26.7% (24.5%-29.1%)	530342 (458792-609014)	21.5% (19.3%-24.0%)	12331 (5956-23564)	90.2% (32.1%-130.5%)	26864 (12865-49225)	21.3% (16.3%-27.1%)	198594 (92879-386142)	74.7% (16.8%-115.6%)
**Australasia**	625086 (539710-712347)	35.0% (29.6%-40.3%)	54525 (46977-62630)	31.1% (26.2%-36.3%)	1455 (697-2744)	220.3% (68.2%-321.1%)	2355 (1114-4265)	31.0% (22.2%-40.6%)	15820 (7596-30166)	168.7% (33.3%-255.2%)
**High-income Asia Pacific**	6036002 (5210182-6898991)	37.4% (31.6%-43.8%)	489495 (426049-565312)	26.1% (20.0%-33.1%)	1282 (651-2114)	240.4% (132.3%-443.2%)	24680 (11717-44731)	42.4% (32.4%-54.9%)	14139 (7621-24364)	141.9% (68.2%-258.8%)
**High-income North America**	13961440 (12552572-15445785)	25.6% (17.7%-35.6%)	1191366 (1063013-1335557)	26.2% (18.1%-35.6%)	15958 (7777-29844)	154.7% (104.4%-193.0%)	50935 (23761-90890)	25.8% (16.1%-36.3%)	209196 (101860-395722)	130.6% (88.2%-163.7%)
**Southern Latin America**	1316077 (1125201-1494772)	38.7% (33.8%-44.4%)	120461 (104212-138563)	32.5% (27.8%-37.9%)	553 (263-1038)	238.3% (147.7%-309.2%)	5636 (2646-10152)	28.7% (21.6%-37.8%)	7536 (3622-14329)	196.1% (115.8%-259.0%)
**Western Europe**	17338264	3.3% (1.4%-5.3%)	1393111	0.2% (-1.8%-2.4%)	21922	120.3%	71984	-3.2% (-6.9%-0.8%)	251178	87.7% (34.8%-120.7%)

	(14957430-19677419)		(1215264-1597268)		(10276-40349)	(62.1%-163.4%)	(33793-130193)		(119208-479586)	
**Andean Latin America**	454206 (392058-520945)	176.2% (167.2%-184.6%)	46920 (40588-53969)	167.0% (158.3%-174.8%)	71 (52-89)	294.3% (200.1%-416.8%)	2185 (1020-3973)	144.1% (130.6%-158.9%)	953 (716-1194)	259.0% (174.7%-370.6%)
**Caribbean**	511218 (440162-585168)	93.7% (89.4%-98.0%)	51482 (44448-59163)	88.3% (84.1%-93.0%)	1024 (537-1803)	183.9% (95.1%-250.9%)	2446 (1166-4399)	80.3% (72.6%-89.1%)	14588 (7719-25844)	153.1% (74.8%-212.9%)
**Central Latin America**	2392336 (2073987-2739949)	153.5% (149.2%-158.2%)	245047 (211434-281535)	146.7% (142.1%-151.3%)	1298 (632-2562)	276.8% (122.4%-422.0%)	11222 (5257-20137)	135.8% (127.1%-146.9%)	17737 (8566-35692)	234.2% (95.4%-366.7%)
**Tropical Latin America**	2233807 (1944381-2556679)	126.4% (121.8%-131.8%)	229885 (198050-263869)	117.7% (113.1%-122.8%)	2794 (1281-5177)	282.5% (174.9%-422.7%)	10643 (5013-19411)	115.4% (105.1%-128.1%)	44094 (20308-83166)	221.3% (133.4%-331.8%)
**North Africa and Middle East**	4483708 (3873089-5120535)	157.0% (153.1%-161.0%)	470128 (407448-537521)	152.8% (148.4%-157.2%)	1339 (1093-1831)	239.2% (154.5%-335.1%)	18907 (8866-34351)	118.9% (111.8%-128.6%)	22229 (18309-28035)	195.8% (113.5%-283.3%)
**South Asia**	12004464 (10387163-13752790)	165.9% (159.7%-172.3%)	1286587 (1106373-1476216)	153.8% (148.0%-160.1%)	2964 (2160-3795)	439.0% (248.3%-585.1%)	58013 (27257-103644)	157.0% (145.7%-172.1%)	49750 (37108-63767)	374.2% (203.9%-512.2%)
**East Asia**	29589160 (25557455-33841453)	172.9% (168.8%-177.3%)	2711137 (2334791-3113092)	153.0% (148.9%-157.0%)	2279 (1874-2830)	262.7% (157.0%-358.9%)	135332 (61923-243445)	133.7% (120.5%-150.0%)	36737 (30206-44656)	202.6% (112.4%-288.0%)
**Oceania**	92183 (78753-105437)	163.2% (155.4%-171.6%)	9894 (8500-11350)	161.5% (152.8%-171.1%)	9 (7-13)	276.0% (191.3%-394.0%)	451 (208-807)	145.2% (130.5%-160.3%)	188 (139-275)	265.3% (179.7%-393.5%)

**Southeast Asia**	8805691 (7584637-10038684)	158.4% (154.4%-162.1%)	876385 (758564-1002747)	149.8% (146.2%-153.2%)	913 (717-1128)	303.4% (224.6%-378.6%)	42887 (20031-76413)	134.6% (126.6%-143.8%)	16561 (12891-20407)	284.0% (204.8%-363.3%)
**Central Sub-Saharan Africa**	489866 (419830-560169)	135.4% (126.5%-144.0%)	56336 (48531-64854)	133.3% (124.2%-142.9%)	800 (408-1271)	130.4% (57.6%-294.4%)	2456 (1129-4402)	116.9% (101.9%-132.0%)	16614 (8574-26885)	122.3% (52.3%-279.1%)
**Eastern Sub-Saharan Africa**	1349014 (1160033-1547847)	126.3% (123.2%-129.5%)	153080 (132225-175448)	124.1% (120.6%-128.0%)	1604 (852-2134)	213.5% (133.2%-302.3%)	6805 (3186-12174)	106.3% (99.7%-114.3%)	30214 (17081-39880)	192.1% (116.0%-279.9%)
**Southern Sub-Saharan Africa**	676050 (584849-774097)	78.2% (75.2%-81.4%)	69933 (60300-80477)	82.5% (79.3%-86.0%)	913 (722-1030)	201.5% (155.0%-246.5%)	3334 (1568-6000)	65.7% (60.8%-71.4%)	18702 (14785-21249)	191.9% (143.9%-239.5%)
**Western Sub-Saharan Africa**	1516753 (1304684-1737206)	123.5% (120.5%-126.8%)	170050 (146947-195012)	125.0% (120.4%-130.0%)	452 (247-603)	183.6% (81.8%-302.8%)	7484 (3475-13479)	99.7% (94.1%-105.7%)	8604 (4520-11702)	172.2% (74.6%-290.9%)

Data in parentheses are 95% uncertainty intervals.

SDI, socio-demographic index; YLDs, years lived with disability; YLLs, years of life lost.
