# Supplementary material for: Burden of Peripheral Artery Disease and Its Attributable Risk Factors in 204 Countries and Territories From 1990 to 2019
Source: Front Cardiovasc Med. 2022 Apr 12;9:868370. doi: 10.3389/fcvm.2022.868370 (PMC9039520; doi:10.3389/fcvm.2022.868370)
Supplement: Supplementary file 2 [file Table_2.pdf]

Table S2. Number of peripheral artery disease prevalent cases, incident cases, deaths, YLDs, and YLLs in 2019, by sex, SDI quintile, and location

	Prevalence		Incidence		Deaths		YLDs		YLLs	
	**Females**	**Males**	**Females**	**Males**	**Females**	**Males**	**Females**	**Males**	**Females**	**Males**
**Global**	76093176 (66587165-86171494)	37349840 (32514726-42646806)	6779114 (5913971-7748629)	3724977 (3237508-4255580)	36353 (15769-74771)	37710 (17621-79468)	337049 (158788-607776)	163844 (76301-293204)	433066 (199652-874595)	602422 (294021-1237247)
**Low SDI**	2594558 (2233371-2986790)	1760047 (1510827-2027349)	289189 (248609-330269)	180929 (156022-207001)	966 (283-1758)	1718 (1011-2363)	12895 (6045-23021)	8984 (4171-15943)	16479 (4787-30403)	34585 (19652-48360)
**Low-middle SDI**	8625565 (7478559-9858042)	4784549 (4123015-5477045)	886656 (767360-1015041)	511653 (439339-587034)	1670 (999-2383)	2051 (1577-2738)	42535 (19929-76994)	23496 (11055-41687)	25570 (15006-35400)	37077 (29115-48166)
**Middle SDI**	21142080 (18226337-24193557)	9434683 (8099524-10822104)	2016503 (1746597-2313794)	898597 (774488-1030187)	3079 (2000-5036)	3565 (2655-5299)	99812 (45772-180607)	41699 (19452-75202)	44316 (29318-71634)	64924 (49513-93003)
**High-middle SDI**	22230932 (19382721-25300155)	8524538 (7375432-9814253)	1909340 (1656985-2183880)	836656 (719297-961717)	12368 (4756-25883)	13876 (5618-31204)	99736 (47085-179657)	36135 (16728-64491)	147610 (58681-308662)	237194 (95730-530631)
**High SDI**	21466185 (18966882-24046883)	12825614 (11257997-14481213)	1674295 (1484428-1897718)	1181446 (1035584-1343117)	18242 (6590-39883)	16472 (5825-38101)	81917 (38412-148749)	53437 (24838-96948)	198738 (72596-438182)	228201 (80080-530753)
**Central Asia**	527948 (456898-603461)	281213	54240 (46786-62487)	31199	46 (19-94)	55 (21-125)	2253	1168	696	1201

		(241907-321698)		(26691-35862)			(1060-4095)	(534-2126)	(298-1422)	(452-2769)
**Armenia**	38088 (32534-43766)	19447 (16906-22299)	3565 (3072-4152)	2007 (1716-2311)	7 (2-14)	5 (2-12)	172 (82-314)	90 (42-160)	86 (30-180)	98 (32-241)
**Azerbaijan**	67544 (58217-78422)	38764 (33247-44534)	7007 (6006-8193)	4273 (3628-4973)	3 (1-4)	2 (2-3)	282 (134-512)	162 (75-297)	38 (23-54)	40 (30-54)
**Georgia**	58163 (49990-67168)	26271 (22569-30192)	5158 (4424-5982)	2678 (2279-3097)	6 (2-11)	6 (2-16)	287 (136-516)	128 (59-230)	67 (20-134)	115 (35-284)
**Kazakhstan**	136466 (117873-157275)	63875 (54710-73193)	13372 (11515-15525)	6922 (5941-8043)	11 (4-23)	12 (4-28)	590 (278-1064)	262 (118-476)	159 (60-337)	267 (90-638)
**Kyrgyzstan**	29963 (25756-34405)	15942 (13724-18280)	3155 (2713-3664)	1795 (1539-2079)	7 (3-17)	12 (4-28)	131 (62-243)	67 (32-121)	101 (37-231)	245 (81-584)
**Mongolia**	15403 (13256-17732)	8595 (7375-9865)	1658 (1431-1908)	972 (841-1127)	1 (0-1)	1 (0-1)	65 (30-120)	35 (16-63)	10 (7-20)	10 (8-13)
**Tajikistan**	29498 (25086-34148)	19653 (16648-22744)	3338 (2823-3882)	2283 (1928-2671)	1 (1-2)	1 (1-2)	121 (56-220)	83 (37-151)	18 (11-24)	25 (19-32)
**Turkmenistan**	27250 (23416-31135)	14984 (12876-17213)	2738 (2339-3161)	1630 (1405-1884)	3 (1-7)	4 (1-10)	120 (57-217)	63 (29-114)	53 (17-103)	102 (33-246)

**Uzbekistan**	125572 (105604-146800)	73682 (62378-85550)	14248 (11909-16623)	8639 (7214-10125)	8 (3-19)	12 (4-28)	484 (223-890)	279 (128-502)	164 (68-379)	300 (102-733)
**Central Europe**	1880121 (1616169-2158014)	969753 (836519-1112751)	165091 (142335-192222)	97398 (83512-111869)	1859 (670-3726)	2145 (826-4985)	8538 (4045-15628)	4315 (2033-7836)	22727 (8320-45317)	37430 (14247-86839)
**Albania**	31099 (26685-35718)	21797 (18618-25213)	2871 (2462-3316)	2185 (1847-2536)	3 (1-4)	5 (4-8)	137 (64-252)	99 (46-180)	37 (19-56)	90 (62-130)
**Bosnia and Herzegovina**	55485 (47495-63930)	33901 (28871-39063)	5143 (4418-5955)	3459 (2934-4012)	11 (6-15)	15 (11-20)	249 (120-463)	152 (72-274)	139 (78-193)	252 (183-330)
**Bulgaria**	123167 (104564-142284)	69492 (59214-80257)	11021 (9412-12890)	6953 (5943-8044)	40 (14-89)	50 (17-123)	587 (273-1095)	328 (154-597)	524 (188-1144)	893 (296-2212)
**Croatia**	71472 (60906-82289)	40327 (34390-46618)	6150 (5311-7200)	3969 (3393-4582)	99 (29-192)	120 (39-293)	306 (145-569)	171 (81-313)	1159 (350-2306)	1989 (654-4774)
**Czechia**	179431 (152937-208225)	106343 (90297-123242)	15348 (13073-18044)	10356 (8900-11981)	218 (81-505)	238 (83-578)	775 (369-1425)	455 (217-833)	2654 (987-6194)	4153 (1418-10069)
**Hungary**	185163 (157353-214886)	94008 (80801-108372)	15928 (13626-18639)	9329 (7990-10740)	436 (156-1108)	575 (191-1446)	833 (390-1527)	411 (196-745)	5475 (2018-13953)	10104 (3431-25053)
**Montenegro**	9290 (7956-10769)	5800 (4942-6716)	850 (726-982)	586 (499-683)	1 (1-2)	2 (1-2)	40 (19-74)	25 (12-45)	15 (10-22)	29 (22-38)

**North Macedonia**	24896 (21306-28668)	17601 (15033-20288)	2440 (2106-2850)	1851 (1573-2145)	3 (1-4)	4 (3-6)	107 (51-199)	77 (36-139)	38 (19-53)	84 (60-115)
**Poland**	667965 (574732-765153)	270095 (232998-311372)	57616 (49818-66889)	27541 (23492-31909)	663 (158-1582)	478 (137-1271)	3084 (1480-5607)	1202 (565-2162)	7731 (1876-18006)	8462 (2432-22398)
**Romania**	293726 (249941-338410)	165734 (142666-190652)	26108 (22293-30323)	16550 (14141-19093)	264 (97-579)	511 (170-1201)	1371 (641-2516)	757 (358-1365)	3401 (1225-7225)	8774 (2896-20351)
**Serbia**	133772 (114418-155746)	86024 (74241-99363)	12448 (10539-14518)	8717 (7481-10139)	61 (24-86)	77 (59-99)	597 (280-1118)	392 (185-718)	829 (320-1174)	1309 (1008-1693)
**Slovakia**	70389 (60018-80398)	38770 (33228-44486)	6343 (5420-7286)	3974 (3368-4627)	44 (12-65)	49 (36-64)	306 (144-558)	164 (78-303)	553 (153-800)	954 (691-1299)
**Slovenia**	34266 (29706-39425)	19862 (17127-22706)	2823 (2438-3283)	1929 (1659-2232)	16 (2-43)	20 (2-51)	145 (69-266)	83 (39-149)	172 (25-487)	337 (22-858)
**Eastern Europe**	4918240 (4253830-5648795)	990416 (856058-1134815)	425386 (368217-488296)	104956 (90137-121039)	5743 (2041-12886)	6588 (2169-16007)	22565 (10844-41580)	4299 (2021-7636)	72861 (26068-163620)	125733 (41024-304182)
**Belarus**	177277 (151927-203920)	58026 (49982-66670)	15793 (13596-18280)	6171 (5294-7185)	94 (24-215)	81 (24-219)	783 (369-1431)	243 (116-439)	1153 (301-2646)	1527 (439-4018)
**Estonia**	30381 (26015-35193)	10130	2425 (2106-2820)	999	4 (1-10)	3 (1-6)	138 (64-257)	44 (20-81)	49 (13-103)	44 (13-101)

		(8679-11646)		(858-1155)						
**Latvia**	45581 (38867-52587)	14437 (12367-16550)	3719 (3198-4328)	1441 (1235-1667)	19 (3-62)	10 (3-41)	214 (100-389)	65 (30-118)	223 (39-723)	177 (45-702)
**Lithuania**	63170 (53607-73327)	20638 (17802-23586)	5134 (4418-5978)	2036 (1751-2341)	42 (13-83)	46 (14-106)	297 (141-551)	94 (43-168)	483 (154-957)	781 (242-1805)
**Republic of Moldova**	57860 (49680-67176)	22647 (19542-26058)	5551 (4740-6444)	2422 (2079-2806)	15 (4-29)	19 (5-52)	269 (129-498)	104 (48-187)	197 (54-408)	346 (99-1068)
**Russian Federation**	3440493 (2979217-3933665)	651677 (562833-744850)	295511 (255007-341855)	69221 (58982-79895)	4154 (1473-9618)	4898 (1650-12002)	15668 (7452-28727)	2801 (1310-4932)	52749 (18444-121093)	94398 (31127-231470)
**Ukraine**	1103479 (943326-1271690)	212862 (183319-244074)	97253 (84139-111491)	22666 (19577-26159)	1414 (476-2969)	1531 (514-3553)	5195 (2508-9526)	949 (444-1705)	18008 (6213-38617)	28460 (9557-65604)
**Australasia**	414624 (358679-471568)	210463 (178554-242228)	34339 (29660-39299)	20186 (17170-23326)	771 (286-1754)	684 (228-1632)	1508 (716-2735)	847 (403-1535)	7407 (2797-17143)	8413 (2818-20040)
**Australia**	349538 (302350-398107)	178490 (151228-206002)	28769 (24743-32980)	17066 (14490-19716)	674 (254-1538)	584 (195-1398)	1265 (605-2279)	716 (341-1284)	6463 (2472-14988)	7216 (2431-17173)
**New Zealand**	65085 (56337-74243)	31973 (27326-37171)	5570 (4814-6409)	3119 (2661-3646)	97 (34-217)	99 (34-240)	243 (116-439)	131 (61-241)	943 (332-2187)	1198 (405-2904)

**High-income Asia Pacific**	4537563 (3913246-5176480)	1498439 (1289946-1720926)	345309 (301449-397954)	144187 (123675-166525)	652 (242-1190)	629 (289-1321)	18273 (8738-33145)	6407 (3000-11633)	5811 (2292-10862)	8328 (3969-17256)
**Brunei Darussalam**	2677 (2308-3068)	1297 (1096-1513)	250 (214-288)	150 (127-175)	1 (1-1)	1 (1-1)	9 (4-16)	5 (2-9)	13 (9-18)	15 (11-19)
**Japan**	3725981 (3211057-4264898)	1139521 (975176-1312567)	275836 (239862-320321)	106316 (91043-123647)	552 (156-1076)	492 (159-1171)	15355 (7346-28034)	5030 (2374-9087)	4756 (1436-9560)	6318 (2066-14960)
**Republic of Korea**	753125 (651569-861288)	329428 (281449-378356)	64375 (55912-74662)	34803 (29584-40423)	81 (47-110)	124 (99-153)	2711 (1259-4964)	1264 (596-2322)	828 (528-1128)	1807 (1442-2228)
**Singapore**	55780 (48381-63199)	28193 (24043-32322)	4847 (4163-5623)	2918 (2472-3386)	19 (4-45)	12 (3-31)	198 (92-366)	108 (50-197)	215 (47-499)	188 (54-501)
**High-income North America**	7912242 (7148780-8722982)	6049198 (5397542-6774730)	633448 (566758-708615)	557919 (495058-624266)	8510 (3082-18643)	7448 (2493-17707)	26548 (12437-47563)	24387 (11325-43983)	101705 (37157-225251)	107491 (35390-257094)
**Canada**	852833 (735709-975892)	645543 (552067-748941)	67925 (58486-78508)	58615 (49868-67725)	745 (266-1631)	684 (223-1671)	2914 (1352-5316)	2593 (1216-4821)	8638 (3195-19274)	9925 (3256-24224)
**Greenland**	730 (634-827)	738 (636-857)	62 (54-72)	77 (65-90)	1 (0-1)	1 (0-1)	3 (1-5)	3 (2-6)	10 (7-17)	11 (8-14)

**United States of America**	7058554 (6384040-7755052)	5402821 (4838541-6025629)	565451 (506864-630808)	499218 (442670-556636)	7765 (2818-17048)	6763 (2270-16107)	23631 (11150-42819)	21791 (10165-39284)	93055 (33953-205625)	97554 (32225-232283)
**Southern Latin America**	867397 (742992-986326)	448680 (383823-519771)	74738 (64665-85702)	45722 (38936-52862)	248 (86-546)	305 (104-732)	3595 (1675-6587)	2041 (953-3656)	2915 (1030-6488)	4621 (1561-11094)
**Argentina**	562224 (479695-645883)	286314 (244613-331945)	48714 (41916-56318)	29418 (24987-34248)	119 (43-268)	138 (47-329)	2384 (1118-4374)	1331 (614-2437)	1392 (508-3110)	2140 (727-5136)
**Chile**	240563 (206237-274127)	131629 (112511-153616)	20798 (17871-23886)	13307 (11386-15431)	124 (42-262)	164 (55-395)	933 (441-1721)	567 (261-1006)	1475 (509-3196)	2437 (820-5962)
**Uruguay**	64566 (55136-73413)	30714 (26244-35559)	5223 (4521-5981)	2996 (2549-3471)	4 (2-10)	3 (1-7)	278 (131-511)	143 (68-259)	48 (18-110)	43 (15-104)
**Western Europe**	11051342 (9538410-12513819)	6286922 (5412205-7202215)	828069 (721431-952886)	565042 (487621-648742)	11219 (3809-24170)	10703 (3647-25385)	44876 (21361-80698)	27108 (12833-48797)	110592 (38388-241341)	140586 (47690-330002)
**Andorra**	1561 (1352-1768)	1112 (949-1278)	117 (102-134)	102 (87-118)	1 (1-2)	1 (1-2)	6 (3-10)	4 (2-8)	14 (7-23)	19 (12-27)
**Austria**	220315 (189210-253306)	127880 (109314-147545)	16428 (14280-19007)	11538 (10016-13304)	343 (122-776)	389 (133-933)	878 (420-1603)	538 (247-993)	3311 (1169-7545)	5176 (1787-12381)
**Belgium**	305058 (262700-348829)	182082 (156301-210007)	22342 (19375-25826)	16237 (14048-18805)	174 (62-403)	155 (52-376)	1223 (572-2225)	779 (367-1443)	1688 (595-3924)	2052 (697-4916)

**Cyprus**	25475 (21932-29348)	16999 (14416-19605)	2074 (1785-2400)	1609 (1365-1856)	11 (4-16)	8 (6-10)	95 (44-177)	72 (33-131)	119 (45-162)	122 (97-151)
**Denmark**	185031 (159587-212466)	124248 (105304-144713)	13654 (11678-15824)	10789 (9165-12667)	195 (73-456)	150 (52-361)	741 (351-1370)	545 (254-979)	2005 (758-4613)	2017 (689-4848)
**Finland**	143321 (123488-164014)	84369 (71516-97893)	10992 (9415-12757)	7712 (6554-9005)	117 (32-238)	90 (29-210)	588 (276-1069)	367 (171-666)	1102 (309-2149)	1163 (384-2729)
**France**	1607187 (1383467-1830059)	905657 (774005-1039488)	119144 (102956-138641)	81373 (69424-94082)	663 (222-1500)	586 (189-1402)	6573 (3128-11974)	3955 (1844-7106)	6084 (2095-14034)	7450 (2370-17812)
**Germany**	2248565 (1947921-2566126)	1383290 (1182226-1594539)	166765 (144449-191741)	122268 (105388-140905)	3845 (1395-8506)	3905 (1342-9076)	9106 (4321-16514)	5977 (2785-10906)	39163 (14033-85473)	52180 (17506-120687)
**Greece**	305742 (262077-350467)	186880 (159857-217799)	22572 (19536-25955)	16038 (13831-18598)	70 (23-146)	48 (16-116)	1257 (594-2330)	837 (395-1538)	707 (231-1465)	605 (202-1419)
**Iceland**	6294 (5449-7134)	4191 (3578-4807)	484 (420-560)	382 (326-442)	3 (1-6)	1 (0-3)	24 (11-44)	17 (8-32)	25 (9-58)	18 (6-44)
**Ireland**	95240 (82350-108233)	63114 (54210-73206)	7405 (6366-8450)	5769 (4956-6725)	125 (46-295)	121 (42-296)	357 (164-640)	260 (121-471)	1311 (468-3203)	1685 (579-4136)
**Israel**	146055 (126547-167078)	91187 (78172-104550)	11661 (10063-13607)	8427 (7232-9723)	170 (63-380)	162 (56-378)	583 (273-1035)	400 (185-736)	1747 (656-3880)	2205 (749-5177)

**Italy**	1908498 (1654816-2160051)	901808 (771754-1033434)	139721 (122190-159810)	81151 (70139-92667)	1755 (603-4052)	1860 (633-4560)	7818 (3711-14312)	3925 (1863-7100)	16649 (5825-39064)	23827 (8068-58508)
**Luxembourg**	13332 (11583-15122)	8335 (7157-9597)	972 (846-1119)	757 (654-877)	6 (2-14)	7 (2-16)	50 (24-91)	34 (16-61)	59 (20-142)	91 (30-224)
**Malta**	12635 (10793-14505)	8040 (6781-9365)	977 (841-1139)	730 (612-850)	13 (4-28)	10 (3-24)	52 (24-96)	36 (16-66)	129 (43-290)	142 (48-337)
**Monaco**	1173 (1010-1340)	748 (638-860)	82 (70-95)	60 (52-70)	0 (0-1)	0 (0-1)	5 (2-9)	3 (2-6)	5 (3-7)	6 (4-7)
**Netherlands**	395583 (342508-449101)	258896 (221178-304667)	30527 (26353-35109)	23562 (20181-27659)	633 (228-1386)	572 (202-1346)	1543 (709-2865)	1090 (509-1965)	6482 (2391-14470)	7893 (2827-18645)
**Norway**	125523 (109015-142320)	70907 (61141-81513)	9443 (8176-10821)	6427 (5545-7426)	69 (24-150)	89 (30-214)	501 (236-913)	300 (142-551)	658 (226-1443)	1174 (394-2852)
**Portugal**	300985 (258940-345812)	166777 (141258-191681)	22880 (19875-26421)	15067 (12942-17344)	408 (138-881)	369 (124-874)	1240 (582-2283)	742 (349-1356)	3995 (1353-8824)	4814 (1636-11325)
**San Marino**	790 (683-897)	501 (426-575)	57 (50-66)	43 (37-50)	1 (0-1)	0 (0-0)	3 (1-6)	2 (1-4)	8 (5-12)	1 (0-1)
**Spain**	1154742 (1001024-1322722)	610463 (523383-702329)	85291 (74154-98529)	55469 (48097-63601)	901 (292-1947)	989 (339-2286)	4699 (2254-8519)	2342 (1068-4259)	8352 (2678-18300)	12656 (4335-29368)
**Sweden**	263015 (226649-300833)	154830 (131373-179021)	19749 (17166-22856)	13615 (11600-15910)	182 (61-383)	114 (37-273)	1078 (512-1927)	794 (369-1446)	1724 (595-3719)	1398 (455-3385)

**Switzerland**	212091 (184412-242086)	130370 (112031-149634)	15464 (13325-17929)	11466 (9871-13257)	153 (56-341)	156 (54-373)	831 (392-1513)	550 (259-998)	1418 (512-3201)	1995 (687-4732)
**United Kingdom**	1363496 (1179031-1547915)	798756 (685984-917219)	108547 (94284-124329)	73958 (63712-84806)	1368 (406-2605)	912 (297-2108)	5586 (2657-10194)	3516 (1682-6428)	13742 (4108-25939)	11775 (3832-27513)
**Andean Latin America**	270740 (232825-311170)	183466 (157315-210099)	27469 (23718-31480)	19451 (16771-22361)	36 (20-48)	34 (27-43)	1279 (597-2323)	906 (425-1639)	457 (248-599)	496 (387-622)
**Bolivia (Plurinational State of)**	42833 (36445-49216)	28801 (24826-33281)	4641 (3951-5394)	3221 (2770-3705)	7 (3-11)	6 (4-9)	213 (101-389)	148 (68-267)	99 (43-160)	95 (62-142)
**Ecuador**	76108 (64810-88072)	50854 (43430-59133)	7748 (6671-8947)	5447 (4650-6255)	9 (5-13)	10 (7-13)	357 (165-642)	249 (116-448)	128 (73-176)	146 (108-192)
**Peru**	151800 (130400-173857)	103811 (89156-118242)	15080 (12997-17287)	10783 (9317-12434)	21 (10-30)	19 (13-26)	709 (334-1320)	509 (236-913)	230 (122-337)	255 (177-355)
**Caribbean**	316144 (272269-362078)	195074 (168049-223713)	30973 (26780-35448)	20509 (17751-23602)	531 (227-1092)	493 (195-1074)	1491 (707-2680)	955 (451-1717)	6826 (2986-13774)	7762 (3105-17203)
**Antigua and Barbuda**	583 (500-667)	364 (311-419)	58 (50-67)	39 (33-45)	1 (0-2)	1 (0-2)	3 (1-5)	2 (1-3)	14 (5-30)	17 (6-41)

**Bahamas**	2218 (1912-2518)	1268 (1089-1458)	223 (193-257)	137 (118-160)	5 (2-11)	4 (1-9)	10 (5-19)	6 (3-11)	72 (26-155)	70 (23-161)
**Barbados**	3085 (2652-3534)	1773 (1506-2049)	297 (255-345)	184 (157-213)	23 (9-49)	11 (4-27)	15 (7-27)	9 (4-16)	308 (116-650)	176 (60-418)
**Belize**	1428 (1234-1639)	1050 (897-1209)	150 (130-173)	115 (99-133)	1 (0-1)	1 (0-2)	7 (3-12)	5 (2-9)	9 (3-20)	12 (4-29)
**Bermuda**	814 (699-936)	439 (376-504)	71 (61-82)	43 (37-50)	3 (1-7)	2 (1-5)	4 (2-7)	2 (1-4)	36 (13-77)	30 (10-68)
**Cuba**	118952 (101462-137650)	73611 (63235-84112)	11469 (9846-13267)	7625 (6578-8794)	295 (103-654)	282 (92-648)	534 (253-979)	344 (165-631)	3785 (1322-8165)	4438 (1420-10301)
**Dominica**	513 (439-590)	336 (289-389)	50 (43-57)	36 (31-42)	1 (1-1)	1 (1-1)	3 (1-5)	2 (1-3)	11 (7-15)	13 (10-17)
**Dominican Republic**	54838 (47185-62743)	35490 (30382-40992)	5450 (4694-6248)	3767 (3248-4387)	14 (5-21)	14 (10-19)	268 (125-487)	179 (84-321)	185 (73-276)	219 (159-297)
**Grenada**	667 (573-769)	406 (344-472)	67 (57-78)	46 (39-53)	2 (1-4)	1 (0-2)	3 (2-6)	2 (1-3)	25 (9-51)	19 (6-46)
**Guyana**	3451 (2953-4011)	2131 (1825-2455)	378 (323-441)	246 (210-287)	2 (1-5)	3 (1-7)	17 (8-30)	10 (5-19)	37 (12-78)	52 (17-131)
**Haiti**	33080 (28102-38310)	21436 (18374-24878)	3850 (3272-4448)	2522 (2167-2926)	30 (15-68)	28 (17-49)	170 (78-300)	116 (55-209)	547 (269-1295)	541 (304-949)
**Jamaica**	19739 (17098-22594)	12520 (10774-14297)	1890 (1643-2168)	1311 (1120-1527)	30 (9-56)	44 (15-106)	97 (46-176)	63 (30-112)	323 (103-656)	696 (236-1704)

**Puerto Rico**	46488 (39624-53721)	25469 (21817-29458)	4053 (3484-4730)	2448 (2102-2852)	90 (33-205)	70 (24-167)	216 (102-391)	123 (58-220)	1018 (371-2299)	967 (328-2308)
**Saint Kitts and Nevis**	359 (306-410)	232 (197-270)	37 (32-43)	26 (22-31)	2 (1-3)	1 (0-2)	2 (1-3)	1 (0-2)	23 (9-51)	15 (5-36)
**Saint Lucia**	1316 (1140-1498)	810 (694-934)	130 (112-150)	87 (75-101)	5 (2-10)	3 (1-7)	6 (3-11)	4 (2-7)	61 (22-132)	49 (17-116)
**Saint Vincent and the Grenadines**	765 (655-874)	567 (483-653)	77 (66-88)	61 (52-71)	1 (0-3)	1 (0-2)	4 (2-7)	3 (1-5)	17 (6-37)	15 (5-35)
**Suriname**	3878 (3319-4438)	2274 (1947-2602)	389 (335-446)	247 (212-285)	0 (0-1)	1 (1-1)	19 (9-35)	11 (5-21)	6 (4-8)	14 (11-18)
**Trinidad and Tobago**	12133 (10421-14044)	7666 (6563-8885)	1177 (999-1379)	808 (691-937)	5 (1-11)	7 (2-17)	60 (28-108)	39 (18-70)	67 (20-132)	117 (36-295)
**United States Virgin Islands**	1127 (958-1297)	624 (528-727)	107 (91-125)	64 (54-75)	4 (3-5)	2 (2-3)	6 (3-10)	3 (1-6)	53 (37-78)	38 (30-48)
**Central Latin America**	1575609 (1365752-1804923)	816727 (704566-937610)	157683 (136897-181031)	87364 (75199-100515)	748 (287-1760)	550 (193-1325)	7285 (3424-13055)	3936 (1854-7076)	9746 (3818-23003)	7992 (2801-19405)
**Colombia**	303654 (262455-346625)	213382 (181533-244999)	29667 (25746-34198)	21954 (18928-25224)	164 (59-403)	143 (47-347)	1396 (651-2503)	1028 (474-1867)	1967 (696-4871)	1949 (650-4725)

**Costa Rica**	31843 (27289-36587)	19075 (16285-22034)	3094 (2663-3586)	1991 (1707-2314)	9 (3-18)	12 (4-28)	141 (67-250)	88 (41-157)	99 (34-211)	159 (53-397)
**El Salvador**	38514 (33090-44191)	20008 (17203-23203)	3789 (3266-4372)	2097 (1790-2438)	5 (2-6)	2 (2-3)	186 (86-336)	102 (48-186)	50 (24-70)	31 (22-41)
**Guatemala**	65409 (56018-75397)	38099 (32492-43836)	6894 (5945-7939)	4194 (3606-4867)	5 (2-10)	4 (1-10)	324 (150-605)	199 (91-357)	66 (25-140)	65 (23-155)
**Honduras**	35333 (30281-40729)	22633 (19301-26075)	3868 (3330-4472)	2543 (2193-2928)	11 (4-16)	9 (6-14)	171 (78-312)	116 (55-209)	150 (57-229)	147 (101-216)
**Mexico**	868024 (751393-994458)	363071 (313283-415962)	86838 (75114-99864)	39234 (34015-45146)	455 (169-1132)	303 (95-758)	3997 (1891-7205)	1741 (814-3113)	6159 (2378-15336)	4512 (1392-11124)
**Nicaragua**	26960 (23286-31014)	15235 (12938-17567)	2856 (2475-3272)	1719 (1472-1988)	3 (2-4)	3 (2-3)	120 (56-224)	70 (32-128)	38 (24-52)	44 (34-56)
**Panama**	24256 (20813-27747)	15916 (13703-18348)	2296 (1975-2665)	1627 (1397-1877)	20 (7-43)	27 (9-68)	112 (52-202)	76 (36-139)	223 (76-497)	370 (122-917)
**Venezuela (Bolivarian Republic of)**	181617 (155848-209178)	109309 (93173-126114)	18381 (15672-21277)	12007 (10176-13919)	77 (28-185)	46 (15-112)	838 (392-1528)	516 (246-932)	993 (354-2399)	715 (239-1718)

**Tropical Latin America**	1432993 (1245305-1644931)	800813 (692091-925839)	143332 (123911-164284)	86552 (74302-99945)	1493 (499-3218)	1301 (423-3109)	6795 (3223-12397)	3848 (1799-6971)	21468 (7069-46589)	22626 (7353-52878)
**Brazil**	1399158 (1216103-1604671)	777642 (671664-898706)	139956 (120983-160423)	84053 (72224-97022)	1482 (488-3213)	1289 (410-3092)	6635 (3146-12122)	3736 (1746-6775)	21320 (6998-46479)	22412 (7134-52651)
**Paraguay**	33835 (29134-38877)	23172 (19826-26885)	3377 (2903-3883)	2500 (2147-2895)	12 (4-17)	12 (9-16)	160 (74-290)	112 (53-205)	148 (55-210)	214 (152-285)
**North Africa and Middle East**	2546991 (2199616-2901552)	1936717 (1676130-2219123)	261640 (227058-299098)	208489 (180329-239432)	553 (412-960)	786 (647-934)	10534 (4934-19393)	8373 (3926-15034)	7828 (5833-12064)	14401 (11799-17298)
**Afghanistan**	67737 (58115-77938)	48822 (42042-56096)	7735 (6622-8835)	5642 (4833-6508)	4 (2-8)	6 (3-9)	304 (141-544)	221 (103-390)	73 (39-140)	110 (59-175)
**Algeria**	197436 (168565-227549)	156867 (133675-181190)	20298 (17387-23463)	16515 (14219-18929)	24 (12-39)	52 (27-86)	817 (386-1485)	701 (337-1267)	351 (182-559)	835 (434-1398)
**Bahrain**	4753 (4059-5462)	6197 (5195-7182)	510 (435-592)	712 (601-836)	1 (1-2)	6 (4-8)	15 (7-28)	19 (9-35)	17 (9-23)	127 (90-174)
**Egypt**	327690 (278473-378555)	306410 (262390-357046)	36207 (30776-42164)	33673 (28866-39317)	28 (11-56)	47 (26-71)	1343 (621-2489)	1409 (662-2514)	485 (193-968)	835 (455-1285)
**Iran (Islamic**	481141 (416188-548528)	307410	48106 (41737-55084)	32149	73 (28-90)	99 (86-111)	1951	1317	1036	1629

**Republic of)**		(267192-349453)		(28026-36800)			(919-3540)	(623-2351)	(388-1280)	(1454-1829)
**Iraq**	149696 (128615-172212)	107866 (91768-124825)	15652 (13483-17999)	11909 (10200-13807)	9 (6-19)	11 (8-14)	613 (287-1128)	452 (210-823)	169 (115-269)	252 (182-331)
**Jordan**	38933 (33346-44990)	33015 (28352-38167)	4125 (3528-4748)	3615 (3106-4197)	2 (1-2)	3 (2-4)	148 (69-270)	129 (59-234)	24 (17-38)	47 (35-64)
**Kuwait**	13010 (11106-14871)	14434 (12440-16434)	1346 (1143-1546)	1503 (1288-1719)	1 (0-2)	1 (0-3)	40 (18-72)	49 (23-88)	10 (5-21)	21 (8-48)
**Lebanon**	43680 (37419-50397)	26975 (23336-31172)	4084 (3512-4725)	2665 (2312-3053)	5 (3-7)	8 (5-11)	179 (84-334)	117 (56-213)	60 (32-88)	115 (71-161)
**Libya**	29738 (25722-33958)	23559 (20307-27079)	3053 (2652-3506)	2506 (2168-2866)	3 (1-4)	4 (2-6)	120 (56-221)	100 (47-178)	35 (19-54)	65 (35-98)
**Morocco**	181411 (155991-208353)	134650 (114910-155045)	19095 (16470-21943)	14676 (12506-16999)	19 (10-29)	33 (20-46)	803 (377-1485)	631 (293-1154)	271 (140-415)	528 (330-722)
**Oman**	8547 (7338-9825)	9424 (7995-10968)	918 (784-1058)	1090 (927-1264)	1 (0-2)	2 (1-4)	30 (14-55)	28 (13-51)	16 (8-29)	50 (24-75)
**Palestine**	13764 (11810-15752)	9586 (8173-11029)	1467 (1269-1691)	1089 (932-1251)	1 (0-1)	1 (1-1)	56 (26-99)	38 (18-69)	11 (7-14)	12 (10-15)

**Qatar**	2784 (2318-3253)	7078 (5917-8348)	322 (270-377)	823 (687-969)	0 (0-0)	1 (1-1)	7 (3-12)	18 (8-32)	7 (2-10)	16 (11-23)
**Saudi Arabia**	70476 (60544-81489)	82902 (70018-95796)	7755 (6579-9000)	9408 (7945-10862)	3 (2-5)	5 (4-6)	216 (99-394)	262 (119-475)	58 (35-83)	94 (70-123)
**Sudan**	82174 (70404-94381)	75004 (64127-85757)	9041 (7752-10387)	8355 (7186-9598)	7 (4-11)	18 (9-28)	360 (167-662)	363 (168-658)	110 (59-165)	297 (143-479)
**Syrian Arab Republic**	67161 (57228-76924)	54610 (46475-63025)	7354 (6310-8458)	6050 (5126-7033)	3 (2-4)	3 (2-5)	266 (121-489)	236 (108-431)	41 (27-59)	59 (42-81)
**Tunisia**	83450 (71660-95975)	58886 (50821-67257)	8180 (7074-9466)	6151 (5299-7097)	10 (5-14)	13 (8-19)	350 (162-639)	257 (121-465)	128 (68-190)	214 (130-322)
**Turkey**	600347 (518274-689285)	391461 (337035-449572)	57022 (49224-65407)	40327 (34547-46470)	355 (230-764)	461 (354-585)	2561 (1207-4693)	1705 (809-3088)	4830 (3269-9282)	8783 (6703-11391)
**United Arab Emirates**	10685 (8961-12504)	27525 (22691-32592)	1277 (1065-1490)	3442 (2833-4124)	1 (0-1)	5 (2-9)	27 (12-50)	60 (27-110)	13 (6-26)	138 (59-277)
**Yemen**	69790 (59705-79786)	52067 (44249-60442)	7825 (6679-9001)	5976 (5109-6899)	5 (2-8)	9 (5-14)	318 (148-570)	252 (119-448)	75 (43-125)	158 (85-259)
**South Asia**	7410946 (6413513-8481254)	4593518 (3959443-5270766)	782190 (673528-896177)	504398 (432483-578542)	1205 (793-1736)	1760 (1262-2567)	35289 (16486-63675)	22724 (10694-40380)	18428 (11967-26820)	31322 (22776-45111)

**Bangladesh**	558882 (477539-642459)	463822 (396638-536034)	59834 (51467-68744)	50116 (42778-57673)	118 (62-184)	219 (107-357)	2629 (1207-4682)	2410 (1135-4313)	1751 (933-2716)	3505 (1705-5743)
**Bhutan**	2507 (2143-2875)	2008 (1728-2297)	260 (222-299)	213 (183-245)	1 (0-1)	1 (1-2)	12 (5-21)	10 (5-18)	10 (5-17)	19 (9-32)
**India**	6115005 (5296125-7010649)	3660716 (3155334-4199165)	641622 (552055-735744)	401717 (346377-461649)	969 (622-1435)	1345 (974-1996)	29212 (13675-52680)	17998 (8390-32018)	14811 (9304-21897)	24140 (17462-35274)
**Nepal**	110599 (94395-128140)	76524 (65267-88857)	12070 (10358-13904)	8476 (7233-9837)	17 (10-28)	31 (17-48)	537 (253-974)	395 (184-708)	267 (153-435)	542 (292-839)
**Pakistan**	623954 (535385-716232)	390449 (332403-451090)	68404 (58987-78213)	43876 (37480-50619)	99 (59-154)	162 (100-248)	2900 (1374-5226)	1911 (884-3422)	1589 (947-2565)	3117 (1882-4819)
**East Asia**	22054532 (19000634-25252302)	7534628 (6442902-8658233)	1966972 (1699324-2261593)	744165 (638978-857112)	949 (649-1405)	1329 (1075-1591)	104388 (47666-188892)	30943 (14332-56358)	13557 (9130-19926)	23180 (18353-28257)
**China**	21315433 (18333833-24451721)	7174204 (6129082-8248961)	1904608 (1643535-2191217)	711273 (609570-819828)	917 (617-1363)	1293 (1040-1551)	100657 (45931-182052)	29403 (13615-53417)	13119 (8622-19352)	22550 (17884-27561)
**Democratic People's Republic of Korea**	321762 (276121-369847)	123126 (105075-142828)	30204 (26080-34581)	13012 (11045-15080)	14 (7-20)	15 (11-20)	1818 (838-3349)	550 (251-976)	215 (114-320)	311 (226-434)

**Taiwan (Province of China)**	417338 (370296-466077)	237298 (203711-271622)	32161 (28394-36190)	19880 (17331-22506)	19 (6-45)	22 (7-52)	1914 (909-3444)	991 (452-1817)	222 (75-539)	320 (110-767)
**Oceania**	51682 (44384-58846)	40501 (34408-46403)	5512 (4736-6298)	4382 (3729-5082)	3 (2-5)	6 (4-9)	257 (119-462)	195 (90-349)	52 (32-85)	136 (96-220)
**American Samoa**	466 (398-533)	315 (269-362)	44 (38-51)	31 (26-36)	0 (0-0)	0 (0-0)	2 (1-4)	1 (1-3)	0 (0-0)	2 (2-3)
**Cook Islands**	233 (200-269)	162 (138-185)	20 (18-23)	15 (13-17)	0 (0-0)	0 (0-0)	1 (1-2)	1 (0-1)	0 (0-1)	1 (1-2)
**Fiji**	7436 (6372-8574)	4865 (4125-5633)	747 (641-865)	510 (436-594)	1 (0-1)	0 (0-0)	38 (18-69)	23 (11-41)	11 (6-16)	8 (6-11)
**Guam**	1629 (1407-1864)	1083 (921-1245)	139 (120-159)	99 (84-114)	0 (0-0)	0 (0-0)	9 (4-15)	5 (2-9)	2 (1-3)	6 (4-7)
**Kiribati**	785 (665-901)	434 (365-504)	84 (72-97)	48 (41-56)	0 (0-0)	0 (0-0)	4 (2-8)	2 (1-4)	1 (1-2)	1 (0-1)
**Marshall Islands**	252 (214-290)	200 (171-232)	28 (24-33)	22 (19-25)	0 (0-0)	0 (0-0)	1 (1-2)	1 (0-2)	0 (0-1)	1 (1-2)
**Micronesia (Federated States of)**	572 (489-662)	379 (321-442)	61 (52-72)	42 (35-49)	0 (0-0)	0 (0-0)	3 (1-5)	2 (1-3)	1 (1-2)	3 (1-4)
**Nauru**	34 (28-39)	21 (18-25)	4 (3-4)	2 (2-3)	0 (0-0)	0 (0-0)	0 (0-0)	0 (0-0)	0 (0-0)	0 (0-0)
**Niue**	22 (19-25)	13 (11-15)	2 (2-2)	1 (1-1)	0 (0-0)	0 (0-0)	0 (0-0)	0 (0-0)	0 (0-0)	0 (0-0)

**Northern Mariana Islands**	398 (338-459)	309 (262-360)	40 (34-47)	31 (26-37)	0 (0-0)	0 (0-0)	2 (1-3)	1 (1-2)	1 (1-2)	2 (1-2)
**Palau**	182 (155-209)	133 (113-153)	18 (16-21)	14 (12-16)	0 (0-0)	0 (0-0)	1 (0-1)	1 (0-1)	0 (0-0)	0 (0-0)
**Papua New Guinea**	31251 (26781-35768)	26239 (22208-30288)	3440 (2915-3951)	2888 (2441-3347)	1 (1-3)	4 (2-7)	153 (70-272)	127 (59-230)	25 (14-50)	86 (52-157)
**Samoa**	1459 (1266-1674)	1020 (876-1171)	141 (122-162)	103 (89-120)	0 (0-0)	0 (0-0)	8 (4-14)	5 (2-9)	2 (1-3)	5 (3-7)
**Solomon Islands**	2232 (1908-2581)	1727 (1469-1984)	252 (215-292)	193 (164-223)	0 (0-0)	0 (0-0)	10 (5-19)	8 (4-14)	3 (2-6)	8 (5-14)
**Tokelau**	12 (10-13)	9 (7-10)	1 (1-1)	1 (1-1)	0 (0-0)	0 (0-0)	0 (0-0)	0 (0-0)	0 (0-0)	0 (0-0)
**Tonga**	818 (708-939)	502 (427-577)	76 (65-87)	50 (43-57)	0 (0-0)	0 (0-0)	5 (2-8)	3 (1-5)	1 (1-1)	3 (2-4)
**Tuvalu**	94 (80-108)	59 (51-68)	9 (8-11)	6 (5-7)	0 (0-0)	0 (0-0)	1 (0-1)	0 (0-1)	0 (0-0)	0 (0-0)
**Vanuatu**	1367 (1167-1568)	1118 (959-1282)	144 (125-166)	118 (101-136)	0 (0-0)	0 (0-0)	8 (4-14)	6 (3-11)	1 (1-2)	5 (3-8)
**Southeast Asia**	5841655 (5044852-6642118)	2964037 (2543109-3382019)	569621 (493710-651067)	306764 (264937-352797)	329 (222-483)	584 (445-741)	29272 (13671-52546)	13616 (6359-24476)	4527 (3086-6620)	12034 (9063-15336)
**Cambodia**	107518 (92175-123154)	52454 (44552-60278)	11045 (9484-12768)	5589 (4788-6442)	5 (3-8)	8 (5-14)	572 (269-1020)	252 (115-442)	82 (53-131)	168 (103-272)

**Indonesia**	2259938 (1942978-2581921)	1064656 (908044-1217201)	226376 (195598-260299)	112924 (97295-130198)	115 (74-182)	248 (144-392)	11238 (5221-20124)	4886 (2253-8741)	1714 (1124-2696)	5249 (2932-8261)
**Lao People's Democratic Republic**	36160 (30990-41405)	23172 (19784-26730)	3725 (3205-4274)	2456 (2100-2848)	2 (1-3)	4 (3-6)	192 (89-346)	117 (54-209)	28 (17-43)	89 (55-131)
**Malaysia**	216635 (186459-248267)	148323 (126355-169761)	20547 (17763-23761)	14401 (12414-16573)	17 (9-23)	18 (13-24)	1029 (481-1881)	678 (314-1240)	247 (132-346)	348 (251-468)
**Maldives**	2197 (1882-2497)	1863 (1601-2142)	214 (184-245)	189 (163-217)	1 (1-1)	0 (0-0)	10 (4-17)	7 (3-13)	12 (8-19)	2 (1-2)
**Mauritius**	18615 (16083-21596)	10423 (8899-11991)	1667 (1432-1944)	1008 (852-1167)	2 (1-5)	3 (1-7)	93 (43-173)	48 (22-86)	26 (9-59)	45 (15-109)
**Myanmar**	486454 (416145-556477)	254040 (217425-291212)	48410 (41871-55645)	26464 (22720-30507)	26 (18-39)	49 (32-69)	2600 (1232-4756)	1265 (581-2270)	370 (258-554)	1017 (653-1429)
**Philippines**	744752 (643507-850738)	358329 (306999-409152)	73398 (63696-83726)	37975 (32666-43816)	36 (25-55)	71 (54-91)	3877 (1803-6937)	1672 (781-2984)	540 (358-806)	1642 (1233-2148)
**Seychelles**	1007 (870-1152)	606 (515-694)	90 (78-103)	60 (51-70)	0 (0-0)	0 (0-0)	5 (2-9)	3 (1-5)	1 (1-2)	4 (2-6)
**Sri Lanka**	239590 (206302-274641)	127829 (109344-147198)	22247 (19125-25798)	12693 (10938-14739)	10 (6-16)	20 (14-29)	1169 (547-2139)	577 (270-1080)	141 (84-217)	413 (272-599)

**Thailand**	832931 (717279-952032)	470477 (404090-538800)	75803 (65267-87665)	45329 (39014-52765)	41 (22-57)	40 (29-53)	4133 (1932-7545)	2188 (1019-4019)	466 (273-645)	566 (401-763)
**Timor-Leste**	6536 (5574-7504)	4485 (3809-5227)	669 (570-775)	460 (392-532)	0 (0-1)	1 (0-1)	37 (17-66)	24 (11-43)	5 (3-8)	14 (7-23)
**Viet Nam**	881668 (756154-1007893)	443496 (379005-510852)	84683 (72824-96845)	46813 (39795-53861)	73 (44-122)	120 (82-162)	4279 (1953-7639)	1880 (869-3457)	888 (552-1461)	2462 (1626-3427)
**Central Sub-Saharan Africa**	318753 (273582-365585)	171113 (146234-196391)	36105 (31050-41606)	20231 (17334-23441)	217 (30-382)	584 (259-970)	1625 (751-2918)	831 (387-1476)	3811 (515-6809)	12803 (5505-21201)
**Angola**	74194 (63334-85494)	40286 (34151-46495)	8276 (7067-9589)	4634 (3962-5366)	35 (5-70)	81 (55-114)	360 (168-674)	194 (88-343)	651 (87-1292)	1786 (1189-2501)
**Central African Republic**	12812 (10900-14783)	6837 (5834-7861)	1523 (1305-1757)	849 (715-980)	6 (1-15)	12 (6-19)	69 (32-123)	34 (16-61)	114 (14-304)	283 (151-471)
**Congo**	15842 (13480-18271)	9794 (8338-11290)	1758 (1510-2024)	1126 (967-1296)	12 (2-21)	32 (20-44)	77 (36-141)	47 (21-83)	217 (28-382)	683 (399-972)
**Democratic Republic of the Congo**	205488 (176175-235485)	108693 (92795-125714)	23496 (20111-27143)	13031 (11152-15158)	153 (21-279)	436 (135-799)	1068 (494-1930)	531 (250-952)	2665 (350-4910)	9575 (2787-17329)
**Equatorial Guinea**	3364 (2887-3847)	1593 (1366-1821)	343 (295-394)	168 (145-194)	3 (0-6)	6 (3-11)	16 (7-30)	8 (4-14)	54 (6-103)	117 (64-211)

**Gabon**	7053 (6046-8034)	3910 (3329-4518)	709 (613-813)	425 (360-495)	7 (1-12)	17 (10-25)	35 (16-63)	19 (9-33)	111 (15-187)	358 (200-528)
**Eastern Sub-Saharan Africa**	812976 (699572-930706)	536038 (460409-617713)	91258 (78943-104520)	61822 (53351-70623)	542 (81-1006)	1062 (724-1431)	4097 (1900-7354)	2708 (1266-4827)	8837 (1282-16831)	21377 (14598-28584)
**Burundi**	19922 (17119-22975)	16212 (13825-18785)	2323 (2007-2669)	1932 (1647-2238)	10 (1-23)	22 (13-34)	102 (47-184)	84 (39-149)	167 (22-400)	470 (270-728)
**Comoros**	2665 (2267-3086)	1648 (1411-1895)	288 (245-333)	183 (159-209)	2 (0-4)	3 (2-4)	14 (7-26)	9 (4-16)	31 (4-59)	55 (35-82)
**Djibouti**	2648 (2260-3043)	2377 (2020-2759)	302 (259-345)	276 (235-320)	2 (0-3)	5 (3-7)	12 (6-22)	11 (5-20)	29 (4-53)	100 (56-160)
**Eritrea**	13156 (11226-15146)	7098 (6029-8260)	1525 (1301-1756)	864 (735-1010)	9 (1-18)	10 (6-16)	66 (31-118)	32 (15-57)	161 (20-338)	238 (151-383)
**Ethiopia**	166503 (142732-191459)	133922 (115754-153506)	18883 (16256-21667)	15176 (13087-17368)	112 (16-203)	292 (155-437)	834 (382-1492)	704 (328-1280)	1795 (264-3296)	5367 (2788-8042)
**Kenya**	121220 (104593-138943)	70154 (60015-80824)	13386 (11575-15333)	8116 (6985-9309)	83 (13-131)	108 (79-147)	596 (275-1068)	334 (155-596)	1314 (195-2060)	2234 (1631-3087)
**Madagascar**	49985 (42621-57048)	35328 (30136-41271)	5834 (5020-6764)	4172 (3528-4881)	38 (5-81)	75 (46-117)	243 (114-428)	174 (81-308)	741 (96-1620)	1704 (1033-2680)

**Malawi**	42230 (36358-48473)	24127 (20584-27916)	4710 (4007-5395)	2843 (2433-3267)	22 (3-41)	38 (23-58)	225 (105-408)	122 (57-221)	335 (49-634)	785 (484-1209)
**Mozambique**	64445 (55076-74703)	36737 (31446-42366)	7253 (6259-8331)	4351 (3697-5040)	39 (5-75)	89 (45-130)	335 (154-597)	186 (87-333)	612 (83-1207)	1927 (943-2839)
**Rwanda**	35115 (29956-40272)	18629 (15918-21479)	3945 (3354-4564)	2172 (1835-2518)	20 (3-44)	26 (14-47)	177 (80-329)	92 (44-165)	338 (45-750)	549 (307-961)
**Somalia**	34634 (29551-40262)	18852 (15883-21741)	4202 (3592-4866)	2384 (2023-2751)	13 (2-36)	17 (9-31)	188 (87-336)	96 (44-175)	257 (33-698)	410 (202-725)
**South Sudan**	17730 (15375-20064)	15132 (12846-17449)	1922 (1665-2209)	1631 (1390-1884)	10 (1-17)	27 (14-43)	87 (41-158)	81 (38-143)	162 (23-296)	534 (268-873)
**Uganda**	73753 (63509-84524)	42111 (36002-48442)	8224 (7058-9484)	4890 (4212-5652)	45 (5-93)	88 (41-137)	372 (176-679)	205 (94-359)	708 (82-1474)	1827 (866-2855)
**United Republic of Tanzania**	134699 (116245-154883)	90407 (77567-104172)	14650 (12672-16785)	10161 (8747-11727)	107 (15-202)	206 (97-339)	678 (315-1209)	466 (217-826)	1674 (231-3191)	4013 (1897-6616)
**Zambia**	33621 (28731-38435)	22877 (19684-26205)	3739 (3202-4260)	2622 (2267-3013)	30 (4-58)	54 (32-87)	165 (77-296)	109 (50-197)	506 (62-944)	1145 (669-1858)
**Southern Sub-**	483373 (419193-550842)	192677	48747 (42032-56102)	21186	371 (223-482)	542 (462-618)	2409	925	6303	12399

**Saharan Africa**		(164888-221087)		(18249-24441)			(1125-4329)	(423-1668)	(3632-8040)	(10362-14399)
**Botswana**	10328 (8868-11876)	5155 (4384-5963)	1077 (922-1248)	577 (489-668)	4 (1-7)	9 (6-13)	46 (22-84)	22 (10-40)	85 (20-146)	242 (158-357)
**Eswatini**	4901 (4163-5704)	2072 (1765-2396)	515 (438-595)	236 (201-275)	2 (0-3)	3 (2-5)	25 (12-46)	10 (5-18)	31 (7-55)	79 (53-123)
**Lesotho**	10306 (8779-11872)	4493 (3822-5161)	1121 (960-1305)	526 (449-609)	4 (1-7)	5 (3-8)	55 (26-102)	23 (10-40)	78 (18-134)	126 (75-199)
**Namibia**	10745 (9271-12331)	5126 (4355-5908)	1084 (938-1255)	555 (479-637)	5 (1-9)	10 (7-15)	53 (25-97)	25 (11-44)	86 (20-153)	235 (155-339)
**South Africa**	394119 (342425-449088)	150454 (128826-172825)	39016 (33660-44886)	16328 (14049-18818)	353 (218-470)	511 (431-587)	1954 (907-3503)	718 (330-1290)	5984 (3557-7813)	11665 (9590-13574)
**Zimbabwe**	52973 (45087-61200)	25376 (21591-29492)	5935 (5082-6911)	2964 (2525-3425)	2 (1-4)	2 (2-3)	275 (129-495)	128 (59-227)	39 (17-61)	52 (39-68)
**Western Sub-Saharan Africa**	867304 (747591-993585)	649449 (558214-744075)	96994 (83795-111290)	73056 (63015-83586)	329 (115-487)	123 (97-154)	4173 (1957-7498)	3311 (1535-5945)	6512 (2258-9729)	2093 (1634-2642)
**Benin**	24764 (21309-28439)	17312 (14894-19968)	2739 (2358-3155)	1975 (1693-2281)	9 (3-14)	3 (2-4)	127 (60-231)	89 (41-161)	166 (56-284)	54 (37-79)
**Burkina Faso**	42861 (36951-49334)	28560 (24448-33092)	4873 (4196-5619)	3293 (2839-3780)	24 (8-45)	6 (4-10)	217 (101-391)	148 (70-266)	501 (162-958)	116 (77-176)

**Cabo Verde**	2601 (2261-2975)	1518 (1306-1740)	251 (218-288)	158 (137-182)	2 (1-3)	0 (0-0)	13 (6-23)	7 (3-13)	32 (15-47)	4 (3-5)
**Cameroon**	54252 (46337-62157)	40931 (34931-47069)	6062 (5216-7022)	4697 (4013-5420)	27 (9-49)	11 (7-17)	265 (125-481)	202 (93-367)	543 (166-1018)	195 (117-318)
**Chad**	21629 (18716-24851)	21081 (18015-24482)	2457 (2119-2820)	2394 (2065-2763)	8 (3-14)	4 (2-6)	112 (52-200)	117 (55-215)	164 (52-295)	78 (43-114)
**Côte d'Ivoire**	47273 (40345-54770)	42178 (35914-49007)	5329 (4557-6155)	4889 (4220-5678)	15 (5-27)	7 (5-10)	231 (109-414)	206 (97-363)	304 (99-574)	133 (88-189)
**Gambia**	5018 (4296-5769)	3760 (3209-4319)	545 (469-621)	424 (365-486)	2 (1-3)	1 (1-1)	26 (12-47)	20 (9-36)	37 (11-65)	14 (9-19)
**Ghana**	83778 (72439-96484)	52522 (44726-60685)	9225 (8032-10649)	5966 (5095-6916)	9 (6-16)	5 (4-7)	407 (190-743)	256 (117-466)	168 (103-328)	95 (68-128)
**Guinea**	23415 (20094-26909)	19419 (16643-22374)	2602 (2238-2983)	2179 (1885-2503)	10 (3-19)	4 (3-6)	126 (60-225)	111 (52-198)	199 (61-382)	68 (45-100)
**Guinea-Bissau**	3452 (2965-3988)	2291 (1954-2637)	398 (343-457)	272 (233-313)	1 (0-2)	0 (0-1)	18 (8-32)	12 (5-21)	27 (9-55)	8 (5-12)
**Liberia**	9535 (8142-10913)	8284 (7119-9493)	1095 (943-1248)	965 (823-1115)	3 (1-6)	1 (1-2)	46 (22-83)	40 (19-72)	62 (21-121)	25 (15-36)
**Mali**	35991 (31097-41047)	31852	4095 (3522-4723)	3602	14 (5-27)	6 (4-9)	184 (85-327)	171 (79-309)	293 (97-545)	96 (62-139)

		(27367-36776)		(3067-4134)						
**Mauritania**	9702 (8365-11143)	8109 (6997-9291)	1061 (913-1235)	874 (755-1010)	5 (1-8)	2 (1-3)	48 (23-87)	43 (20-77)	92 (27-166)	27 (17-43)
**Niger**	33122 (28264-38136)	25666 (21869-29515)	3875 (3335-4465)	2986 (2542-3472)	11 (4-22)	4 (2-6)	167 (79-307)	138 (64-248)	238 (77-489)	75 (37-114)
**Nigeria**	395194 (340034-453253)	291684 (250937-332978)	44070 (38017-50698)	32211 (27925-36881)	160 (52-269)	56 (36-83)	1804 (849-3248)	1467 (685-2627)	3135 (1011-5336)	915 (572-1373)
**Sao Tome and Principe**	522 (448-602)	393 (337-452)	57 (49-66)	44 (38-50)	0 (0-0)	0 (0-0)	2 (1-4)	2 (1-3)	4 (2-8)	2 (1-2)
**Senegal**	37225 (32018-42930)	27932 (23968-32205)	4085 (3495-4685)	3106 (2688-3588)	15 (5-25)	7 (4-9)	192 (89-349)	150 (69-272)	296 (98-520)	109 (72-148)
**Sierra Leone**	17795 (15359-20553)	14543 (12430-16818)	1985 (1722-2282)	1659 (1437-1905)	5 (2-10)	2 (2-3)	92 (42-167)	77 (37-138)	99 (33-192)	39 (26-59)
**Togo**	19162 (16361-22190)	11405 (9682-13140)	2187 (1875-2542)	1360 (1164-1563)	7 (3-13)	2 (1-3)	95 (45-174)	53 (24-96)	152 (52-273)	41 (27-59)

Data in parentheses are 95% uncertainty intervals.
SDI, socio-demographic index; YLDs, years lived with disability; YLLs, years of life lost.
