# Supplementary material for: Burden of Peripheral Artery Disease and Its Attributable Risk Factors in 204 Countries and Territories From 1990 to 2019
Source: Front Cardiovasc Med. 2022 Apr 12;9:868370. doi: 10.3389/fcvm.2022.868370 (PMC9039520; doi:10.3389/fcvm.2022.868370)
Supplement: Supplementary file 3 [file Table_3.pdf]

Table S3. Age-standardized prevalence, incidence, death, YLD, and YLL rates of peripheral artery disease for both sexes in 2019, and their percentage changes from 1990 to 2019, by country and territory

	Prevalence		Incidence		Deaths		YLDs		YLLs	
	Age-standardized rate per 100 000 people	Percentage change in age-standardized rates	Age-standardized rate per 100 000 people	Percentage change in age-standardized rates	Age-standardized rate per 100 000 people	Percentage change in age-standardized rates	Age-standardized rate per 100 000 people	Percentage change in age-standardized rate	Age-standardized rate per 100 000 people	Percentage change in age-standardized rates
**Global**	1401.8 (1228.5-1589.4)	-21.7% (-22.8%--20.5%)	127.1 (111.3-145.4)	-18.9% (-19.8%--18.0%)	1.0 (0.6-1.7)	-2.5% (-21.9%-9.8%)	6.3 (3.0-11.3)	-26.5% (-27.7%--25.2%)	13.3 (7.7-22.6)	-4.2% (-22.3%-8.8%)
**Low SDI**	938.6 (815.0-1074.6)	0.5% (-0.4%-1.5%)	94.1 (81.5-107.4)	-2.9% (-3.8%--1.9%)	0.7 (0.4-1.0)	27.0% (-2.4%-66.4%)	5.3 (2.5-9.5)	-7.3% (-9.5%--4.4%)	11.5 (6.5-15.6)	20.9% (-7.7%-58.0%)
**Low-middle SDI**	1042.2 (905.4-1190.1)	-1.1% (-2.2%--0.1%)	103.2 (89.5-118.2)	-2.1% (-3.0%--1.1%)	0.4 (0.3-0.5)	55.4% (21.0%-82.4%)	5.5 (2.6-9.9)	-12.2% (-14.5%--9.1%)	5.2 (4.0-6.6)	53.5% (19.3%-81.5%)
**Middle SDI**	1264.8 (1098.3-1446.0)	1.1% (0.1%-2.0%)	114.7 (99.6-131.5)	-3.4% (-4.3%--2.4%)	0.4 (0.3-0.5)	29.3% (4.3%-49.8%)	6.3 (2.9-11.3)	-13.9% (-16.9%--10.0%)	4.9 (3.8-6.8)	26.5% (3.1%-46.9%)
**High-middle SDI**	1506.1 (1314.6-1711.8)	-15.4% (-16.4%--14.2%)	133.6 (116.2-153.1)	-15.0% (-16.0%--14.1%)	1.4 (0.7-2.4)	-5.3% (-25.2%-11.4%)	6.7 (3.1-12.0)	-23.9% (-25.4%--22.3%)	19.0 (10.0-34.7)	-8.4% (-30.2%-8.4%)
**High SDI**	1794.0 (1585.1-2006.1)	-34.0% (-35.8%--32.0%)	157.7 (139.5-178.5)	-32.1% (-33.9%--30.2%)	1.5 (0.7-2.8)	7.3% (-17.9%-22.0%)	6.7 (3.1-12.1)	-38.1% (-40.0%--36.1%)	20.6 (10.2-38.4)	7.5% (-16.7%-21.1%)

**Central Asia**	1218.1 (1060.0-1392.9)	0.8% (-1.1%-2.8%)	118.2 (102.5-135.6)	-0.6% (-2.5%-1.4%)	0.2 (0.1-0.3)	71.8% (28.2%-116.7%)	5.8 (2.8-10.5)	-6.9% (-9.9%--3.2%)	3.0 (1.5-5.4)	61.8% (19.3%-94.9%)
**Armenia**	1396.8 (1207.7-1597.0)	-3.3% (-7.3%-1.4%)	134.3 (116.0-154.0)	-4.1% (-8.0%-0.6%)	0.3 (0.1-0.5)	17.2% (-56.0%-119.1%)	6.3 (3.0-11.4)	-13.4% (-20.4%--6.7%)	4.5 (2.1-8.3)	14.1% (-62.6%-105.3%)
**Azerbaijan**	1256.5 (1093.1-1440.1)	-2.6% (-6.5%-1.8%)	121.4 (105.3-139.3)	-3.0% (-7.2%-1.5%)	0.1 (0.1-0.1)	76.2% (13.1%-222.9%)	6.1 (2.9-11.0)	-11.5% (-18.2%--4.3%)	1.2 (0.9-1.5)	65.9% (13.5%-161.0%)
**Georgia**	1368.5 (1182.4-1573.4)	8.2% (3.7%-13.2%)	131.9 (114.0-152.1)	8.6% (4.0%-13.5%)	0.2 (0.1-0.4)	62.7% (8.2%-348.0%)	6.4 (3.0-11.7)	3.8% (-3.8%-12.3%)	2.9 (1.2-6.2)	67.7% (11.6%-423.0%)
**Kazakhstan**	1246.2 (1080.1-1430.8)	-4.4% (-8.5%-0.3%)	118.4 (102.5-136.8)	-5.6% (-9.8%--1.0%)	0.2 (0.1-0.3)	64.7% (19.8%-118.5%)	5.7 (2.7-10.4)	-13.7% (-21.1%--5.1%)	2.7 (1.3-5.0)	58.0% (13.6%-105.0%)
**Kyrgyzstan**	1086.9 (940.4-1250.5)	-1.9% (-6.2%-2.5%)	111.1 (96.1-128.0)	-0.5% (-4.7%-4.0%)	0.5 (0.2-1.1)	55.5% (1.2%-93.2%)	5.3 (2.5-9.7)	-9.5% (-16.1%--2.2%)	8.2 (3.8-16.5)	38.6% (-14.6%-77.3%)
**Mongolia**	1169.7 (1015.1-1339.9)	-1.7% (-6.3%-2.6%)	116.0 (100.6-133.3)	-3.4% (-7.8%-1.1%)	0.1 (0.1-0.2)	33.4% (0.4%-86.1%)	6.0 (2.8-10.9)	-12.4% (-19.8%--4.7%)	1.3 (1.0-1.9)	23.2% (-7.8%-67.4%)
**Tajikistan**	1122.6 (973.1-1291.3)	-11.4% (-16.0%--6.8%)	114.9 (99.3-131.8)	-9.2% (-13.7%--4.1%)	0.1 (0.1-0.1)	42.2% (0.1%-153.0%)	5.8 (2.7-10.6)	-16.3% (-23.3%--9.0%)	1.3 (0.9-1.7)	45.0% (7.3%-121.9%)
**Turkmenistan**	1186.9	-1.5% (-5.6%-3.1%)	114.4 (98.6-131.0)	-3.6% (-7.6%-1.1%)	0.2 (0.1-0.5)	78.8%	5.8 (2.8-10.6)	-9.9%	4.2 (1.8-8.0)	84.9%

	(1027.4-1345.8)					(18.6%-136.2%)		(-16.9%--2.0%)		(22.7%-148.3%)
**Uzbekistan**	1117.1 (961.3-1277.5)	11.8% (6.7%-17.0%)	112.4 (97.2-128.9)	9.6% (4.7%-14.6%)	0.2 (0.1-0.3)	139.5% (41.1%-386.8%)	5.4 (2.5-9.8)	3.5% (-5.8%-12.2%)	2.9 (1.3-5.5)	135.0% (30.8%-326.5%)
**Central Europe**	1313.0 (1140.8-1498.9)	-10.5% (-11.6%--9.5%)	124.1 (107.3-142.0)	-10.7% (-11.7%--9.7%)	1.8 (0.9-3.3)	25.5% (-15.0%-73.7%)	5.6 (2.7-10.2)	-20.5% (-22.1%--18.7%)	27.3 (13.2-51.5)	20.5% (-16.2%-58.1%)
**Albania**	1224.8 (1058.1-1399.1)	-2.9% (-6.7%-1.3%)	117.5 (101.5-134.0)	-4.1% (-8.1%-0.2%)	0.2 (0.1-0.3)	77.6% (20.9%-153.9%)	5.3 (2.5-9.6)	-16.5% (-22.7%--9.8%)	2.9 (2.1-4.0)	79.4% (20.6%-160.2%)
**Bosnia and Herzegovina**	1502.5 (1297.3-1713.8)	10.3% (5.3%-15.6%)	143.8 (124.0-164.6)	5.7% (1.2%-10.4%)	0.4 (0.3-0.6)	80.0% (32.2%-150.7%)	6.6 (3.2-12.2)	-2.8% (-10.4%-4.9%)	6.5 (4.9-8.3)	72.2% (26.4%-141.2%)
**Bulgaria**	1303.4 (1124.4-1492.7)	-4.4% (-8.3%--0.4%)	125.3 (108.0-144.1)	-5.2% (-9.1%--1.2%)	0.6 (0.3-1.2)	18.7% (-13.5%-73.4%)	5.8 (2.7-10.5)	-10.3% (-16.9%--3.8%)	9.6 (4.3-19.3)	15.0% (-17.2%-68.0%)
**Croatia**	1258.3 (1082.5-1439.8)	-18.9% (-22.3%--15.1%)	119.0 (102.8-136.6)	-17.2% (-21.1%--13.3%)	2.3 (1.0-4.3)	8.1% (-23.2%-46.7%)	5.0 (2.4-9.3)	-26.2% (-32.3%--20.0%)	34.3 (14.6-68.7)	5.0% (-28.0%-46.9%)
**Czechia**	1346.7 (1162.5-1542.5)	-7.8% (-11.6%--4.0%)	125.0 (107.8-143.9)	-7.9% (-11.6%--3.9%)	2.0 (1.0-4.1)	8.1% (-31.1%-56.7%)	5.4 (2.6-9.9)	-17.5% (-23.6%--10.9%)	31.5 (14.7-65.0)	7.2% (-34.1%-55.9%)
**Hungary**	1421.9	-11.4%	133.3 (116.1-153.1)	-11.4%	4.9 (1.9-10.4)	5.6%	5.9 (2.8-10.8)	-19.7%	78.5 (32.9-169.5)	4.8%

	(1229.0-1629.5)	(-15.2%--7.4%)		(-15.2%--7.4%)		(-59.8%-45.9%)		(-25.3%--13.3%)		(-60.1%-47.1%)
**Montenegro**	1539.3 (1331.9-1759.3)	-1.9% (-5.9%-2.5%)	145.1 (125.3-166.3)	-1.3% (-5.4%-2.9%)	0.3 (0.2-0.4)	24.4% (-7.6%-71.6%)	6.5 (3.2-12.0)	-8.7% (-14.4%--1.6%)	4.5 (3.6-5.8)	19.3% (-11.6%-60.4%)
**North Macedonia**	1362.2 (1182.2-1557.0)	-2.9% (-6.8%-1.1%)	131.9 (114.2-151.4)	-3.1% (-7.3%-1.3%)	0.3 (0.2-0.4)	4.4% (-24.6%-50.7%)	5.9 (2.8-11.0)	-14.3% (-20.6%--7.6%)	4.2 (3.1-5.4)	2.1% (-26.3%-43.8%)
**Poland**	1324.8 (1151.1-1518.8)	-17.6% (-18.8%--16.4%)	123.9 (107.5-142.0)	-17.6% (-18.8%--16.4%)	1.5 (0.5-3.5)	69.4% (-21.3%-1810.1%)	5.7 (2.7-10.3)	-27.7% (-29.7%--25.2%)	22.3 (8.2-51.2)	53.1% (-24.1%-1677.4%)
**Romania**	1212.8 (1045.8-1384.9)	-6.1% (-10.0%--1.9%)	116.4 (100.8-133.3)	-7.3% (-11.6%--3.1%)	1.9 (0.9-3.8)	23.4% (-12.5%-56.8%)	5.3 (2.5-9.5)	-16.9% (-23.0%--10.5%)	31.9 (13.6-63.4)	25.0% (-11.3%-61.9%)
**Serbia**	1377.6 (1193.5-1578.5)	2.9% (-1.4%-7.3%)	133.2 (116.1-153.2)	2.2% (-2.3%-6.5%)	0.9 (0.6-1.2)	201.2% (65.5%-348.3%)	5.9 (2.8-10.8)	-9.6% (-16.2%--2.6%)	13.5 (9.5-17.7)	171.8% (58.3%-297.2%)
**Slovakia**	1184.6 (1017.6-1348.1)	-17.9% (-21.7%--13.7%)	112.0 (96.5-127.9)	-18.1% (-21.7%--14.3%)	1.0 (0.6-1.4)	292.8% (95.3%-478.3%)	4.9 (2.3-9.0)	-25.8% (-31.4%--19.4%)	16.2 (10.3-21.8)	269.9% (106.3%-434.6%)
**Slovenia**	1228.8 (1071.3-1397.5)	-11.7% (-15.7%--7.9%)	114.5 (99.6-132.3)	-11.2% (-15.0%--7.2%)	0.7 (0.1-1.5)	-36.6% (-97.6%-64.9%)	4.8 (2.4-8.8)	-20.6% (-26.1%--14.8%)	11.2 (1.1-24.5)	-30.9% (-97.8%-68.0%)
**Eastern Europe**	1690.4 (1467.3-1936.0)	-1.9% (-3.3%--0.5%)	153.7 (133.6-175.5)	-1.9% (-3.3%--0.6%)	3.5 (1.7-6.7)	31.6% (-7.4%-63.5%)	7.5 (3.6-13.7)	-10.4% (-12.7%--7.7%)	56.1 (26.2-109.4)	33.1% (-9.7%-67.0%)

**Belarus**	1463.7 (1262.5-1672.9)	-1.5% (-6.2%-3.2%)	138.8 (120.5-159.4)	-3.5% (-8.0%-0.8%)	1.1 (0.4-2.3)	69.9% (13.5%-131.7%)	6.2 (2.9-11.5)	-12.0% (-19.7%--4.0%)	16.4 (6.0-36.9)	69.6% (13.3%-130.0%)
**Estonia**	1489.9 (1288.7-1705.7)	-4.0% (-8.6%-0.7%)	135.9 (117.5-155.8)	-6.1% (-10.3%--1.5%)	0.2 (0.1-0.4)	92.9% (-1.6%-535.3%)	6.2 (3.0-11.4)	-15.4% (-22.4%--7.8%)	3.2 (1.2-6.1)	79.6% (-2.3%-500.3%)
**Latvia**	1455.4 (1251.9-1667.5)	7.6% (2.9%-12.9%)	134.5 (116.2-154.9)	4.7% (-0.1%-9.9%)	0.6 (0.2-2.1)	161.3% (9.8%-2368.9%)	6.3 (2.9-11.3)	-1.8% (-9.6%-6.6%)	9.2 (2.9-30.7)	145.7% (7.0%-2415.9%)
**Lithuania**	1407.2 (1207.7-1621.3)	-6.2% (-10.5%--1.8%)	129.5 (111.5-149.5)	-8.1% (-12.4%--3.8%)	1.3 (0.6-2.4)	66.7% (10.6%-448.5%)	6.1 (2.9-11.3)	-11.9% (-18.4%--4.4%)	20.7 (8.5-39.3)	64.4% (7.2%-468.9%)
**Republic of Moldova**	1389.8 (1203.0-1600.3)	5.9% (1.3%-11.2%)	137.8 (118.5-158.4)	6.0% (1.3%-11.1%)	0.6 (0.2-1.3)	84.3% (-7.6%-1403.3%)	6.3 (3.0-11.7)	-3.1% (-10.7%-5.4%)	9.3 (3.7-24.0)	85.2% (-4.4%-1868.9%)
**Russian Federation**	1725.6 (1496.6-1973.1)	-1.8% (-2.6%--0.9%)	154.9 (134.0-177.4)	-1.9% (-2.8%--1.1%)	3.8 (1.9-7.4)	31.4% (-21.3%-76.1%)	7.6 (3.6-13.9)	-10.9% (-12.6%--9.2%)	61.2 (28.6-122.4)	32.4% (-25.2%-78.0%)
**Ukraine**	1696.3 (1467.0-1944.4)	-3.1% (-8.1%-1.7%)	157.5 (136.9-179.5)	-1.8% (-6.6%-3.0%)	3.8 (1.8-7.0)	27.2% (2.4%-55.5%)	7.7 (3.7-14.1)	-9.4% (-17.4%--0.1%)	59.4 (27.8-113.3)	30.0% (2.2%-65.6%)
**Australasia**	1254.3 (1086.1-1424.4)	-37.0% (-39.3%--34.4%)	113.3 (98.3-130.1)	-35.7% (-37.9%--33.1%)	2.5 (1.2-4.7)	14.0% (-40.3%-50.8%)	4.5 (2.1-8.1)	-41.1% (-44.7%--37.3%)	28.4 (13.6-54.6)	9.2% (-45.7%-43.6%)
**Australia**	1257.8	-36.4%	113.2 (97.9-129.8)	-35.2%	2.5 (1.2-4.8)	13.4%	4.5 (2.1-8.1)	-40.6%	29.1 (13.9-56.1)	8.9%

	(1088.2-1428.7)	(-39.3%--33.5%)		(-37.9%--32.2%)		(-41.7%-50.7%)		(-44.9%--35.9%)		(-46.4%-43.8%)
**New Zealand**	1235.1 (1070.2-1401.5)	-39.7% (-42.3%--37.3%)	114.1 (98.8-130.6)	-37.9% (-40.7%--35.4%)	2.2 (1.0-4.1)	15.9% (-32.7%-50.8%)	4.5 (2.1-8.1)	-43.2% (-47.3%--38.9%)	24.7 (11.9-47.7)	9.2% (-41.2%-41.8%)
**High-income Asia Pacific**	1303.9 (1132.7-1480.4)	-41.6% (-42.3%--40.8%)	115.5 (100.5-131.8)	-39.3% (-40.2%--38.4%)	0.2 (0.1-0.4)	-7.6% (-38.9%-37.3%)	4.8 (2.3-8.8)	-46.5% (-47.6%--45.4%)	2.7 (1.5-4.6)	-14.6% (-41.6%-19.7%)
**Brunei Darussalam**	1490.0 (1291.5-1704.8)	-33.0% (-35.8%--30.2%)	131.2 (114.2-150.4)	-29.7% (-32.6%--26.7%)	1.2 (0.9-1.5)	25.8% (-12.4%-75.8%)	6.2 (2.9-11.5)	-38.3% (-42.8%--33.0%)	14.8 (11.7-18.8)	14.1% (-20.4%-56.3%)
**Japan**	1336.5 (1163.3-1515.6)	-41.4% (-42.1%--40.8%)	118.0 (102.4-135.1)	-39.0% (-39.8%--38.2%)	0.2 (0.1-0.4)	-2.6% (-39.1%-53.0%)	4.9 (2.3-9.0)	-45.9% (-46.9%--44.9%)	2.6 (1.1-4.9)	-6.2% (-40.2%-39.5%)
**Republic of Korea**	1212.8 (1052.7-1381.3)	-37.9% (-40.9%--35.0%)	110.2 (95.2-126.2)	-38.1% (-41.1%--35.1%)	0.3 (0.2-0.3)	-33.8% (-53.1%--13.3%)	4.5 (2.1-8.2)	-48.7% (-52.7%--44.6%)	3.1 (2.5-3.7)	-45.3% (-61.1%--28.2%)
**Singapore**	1090.8 (943.8-1236.6)	-37.5% (-40.0%--34.5%)	98.0 (85.0-112.7)	-37.5% (-39.9%--34.8%)	0.4 (0.1-1.0)	28.9% (-41.5%-410.0%)	4.1 (1.9-7.5)	-44.1% (-48.2%--39.7%)	5.5 (1.9-12.7)	20.6% (-47.8%-352.4%)
**High-income North America**	2214.3 (1986.7-2433.8)	-29.2% (-33.7%--23.7%)	193.5 (173.7-215.6)	-28.8% (-33.1%--23.8%)	2.2 (1.1-4.2)	30.9% (5.6%-50.7%)	7.7 (3.6-13.7)	-30.4% (-35.2%--24.8%)	31.6 (15.3-59.8)	25.3% (2.2%-43.5%)
**Canada**	2175.2	-41.8%	189.9	-39.1%	1.8	12.0%	7.6	-43.7%	25.5	8.6%

	(1881.7-2472.5)	(-44.4%--39.0%)	(165.0-215.8)	(-42.0%--36.1%)	(0.9-3.4)	(-29.1%-41.1%)	(3.5-13.9)	(-47.5%--39.5%)	(12.7-48.3)	(-32.7%-35.5%)
**Greenland**	2252.3 (1960.7-2570.8)	-38.7% (-41.1%--36.4%)	197.8 (172.4-226.9)	-36.8% (-39.3%--34.2%)	2.5 (1.9-3.2)	-21.1% (-39.8%-3.0%)	9.5 (4.5-17.2)	-40.6% (-44.8%--36.5%)	34.8 (26.9-46.0)	-24.0% (-43.3%-0.6%)
**United States of America**	2219.2 (1998.2-2434.4)	-27.7% (-32.7%--21.4%)	193.9 (174.5-215.5)	-27.5% (-32.3%--21.6%)	2.3 (1.1-4.3)	33.2% (6.7%-53.9%)	7.7 (3.6-13.7)	-28.7% (-34.2%--22.1%)	32.3 (15.6-60.9)	27.3% (3.6%-46.3%)
**Southern Latin America**	1568.2 (1344.5-1775.4)	-25.7% (-28.1%--23.0%)	145.0 (125.3-166.7)	-26.1% (-28.5%--23.2%)	0.6 (0.3-1.2)	54.9% (14.2%-89.0%)	6.6 (3.1-11.9)	-33.0% (-36.5%--29.0%)	8.8 (4.2-16.7)	50.4% (10.1%-82.9%)
**Argentina**	1561.9 (1337.0-1775.6)	-25.3% (-28.4%--21.5%)	145.2 (125.0-167.7)	-25.4% (-28.8%--21.4%)	0.5 (0.2-0.9)	28.2% (-4.5%-51.3%)	6.7 (3.1-12.1)	-31.8% (-36.5%--26.1%)	6.4 (3.0-12.3)	25.2% (-8.1%-45.5%)
**Chile**	1545.9 (1328.5-1771.4)	-25.0% (-28.4%--21.2%)	141.8 (122.4-162.7)	-26.2% (-29.5%--22.5%)	1.2 (0.6-2.2)	55.9% (7.0%-109.3%)	6.2 (2.9-11.2)	-34.5% (-39.0%--29.7%)	16.2 (7.7-31.0)	53.1% (5.1%-107.4%)
**Uruguay**	1727.4 (1486.8-1966.7)	-27.5% (-30.7%--24.3%)	157.8 (136.5-179.9)	-27.5% (-30.5%--24.4%)	0.1 (0.1-0.2)	24.1% (-36.0%-50.0%)	7.2 (3.4-13.2)	-33.0% (-37.9%--28.0%)	1.5 (0.8-2.9)	17.8% (-46.7%-44.9%)
**Western Europe**	1902.5 (1659.4-2145.4)	-34.2% (-35.1%--33.3%)	165.2 (143.3-189.1)	-32.5% (-33.6%--31.5%)	1.9 (0.9-3.5)	11.0% (-20.5%-32.6%)	7.3 (3.4-13.2)	-40.4% (-41.8%--39.0%)	23.9 (11.4-45.4)	7.0% (-23.7%-27.6%)
**Andorra**	1874.6 (1618.5-2134.4)	-26.2% (-29.5%--22.9%)	156.8 (135.7-179.1)	-25.4% (-28.7%--22.0%)	1.7 (1.0-2.5)	102.0% (16.0%-216.3%)	7.1 (3.4-12.8)	-33.0% (-37.4%--27.3%)	22.0 (13.2-31.8)	90.8% (10.6%-198.6%)

**Austria**	1941.6 (1682.6-2210.2)	-26.0% (-29.0%--22.5%)	167.7 (145.2-191.8)	-25.9% (-29.3%--22.2%)	3.3 (1.6-6.4)	19.3% (-18.4%-53.6%)	7.4 (3.5-13.5)	-32.8% (-37.4%--27.2%)	42.1 (19.9-81.9)	19.1% (-20.9%-52.9%)
**Belgium**	2138.0 (1851.4-2441.6)	-25.5% (-28.7%--22.3%)	183.0 (159.1-211.1)	-25.0% (-28.3%--21.7%)	1.1 (0.5-2.1)	15.8% (-15.1%-41.1%)	8.2 (3.9-15.0)	-32.9% (-38.0%--27.8%)	14.2 (6.8-28.3)	14.2% (-15.5%-39.6%)
**Cyprus**	2206.0 (1912.5-2509.0)	-22.1% (-25.3%--18.7%)	189.0 (162.8-217.1)	-22.6% (-25.8%--19.1%)	1.3 (0.7-1.6)	32.4% (-13.2%-104.8%)	8.4 (4.0-15.4)	-33.5% (-38.1%--28.6%)	13.8 (8.8-17.1)	21.4% (-17.6%-84.1%)
**Denmark**	2702.0 (2342.0-3069.7)	-35.1% (-37.7%--32.4%)	224.9 (194.6-257.5)	-32.4% (-35.3%--29.2%)	2.6 (1.2-4.8)	9.0% (-33.8%-38.4%)	10.4 (4.9-19.0)	-40.7% (-44.5%--36.4%)	31.7 (15.4-62.4)	1.6% (-40.4%-31.4%)
**Finland**	1870.1 (1620.9-2116.1)	-33.5% (-36.3%--30.1%)	163.0 (141.6-186.0)	-32.1% (-35.1%--28.8%)	1.3 (0.5-2.4)	19.8% (-15.2%-53.1%)	7.1 (3.3-12.8)	-40.3% (-44.7%--35.8%)	16.0 (7.0-28.5)	15.4% (-21.0%-46.4%)
**France**	1840.4 (1590.2-2078.0)	-31.0% (-34.1%--27.8%)	159.6 (137.0-183.6)	-29.9% (-33.3%--26.6%)	0.7 (0.3-1.3)	-1.3% (-30.4%-41.4%)	7.1 (3.3-12.7)	-38.3% (-42.8%--33.8%)	8.3 (3.7-16.3)	-3.2% (-32.1%-44.4%)
**Germany**	1902.4 (1653.3-2157.6)	-35.7% (-38.3%--32.8%)	165.2 (143.4-190.5)	-34.2% (-36.9%--31.2%)	3.3 (1.6-6.2)	18.9% (-18.9%-139.2%)	7.3 (3.4-13.2)	-42.3% (-46.4%--37.9%)	42.0 (20.1-78.4)	13.6% (-23.7%-132.3%)
**Greece**	2074.4 (1794.7-2361.8)	-28.7% (-31.5%--25.6%)	180.4 (155.0-207.4)	-27.5% (-30.8%--24.5%)	0.4 (0.2-0.7)	5.8% (-19.5%-27.8%)	8.1 (3.8-14.7)	-33.3% (-38.3%--28.2%)	4.5 (2.1-8.3)	5.2% (-19.9%-28.3%)
**Iceland**	1891.2	-33.4%	162.8 (141.3-187.1)	-32.1%	0.6 (0.3-1.1)	4.3%	7.1 (3.3-12.9)	-38.4%	6.8 (3.2-12.9)	-3.9%

	(1643.7-2141.3)	(-36.2%--30.5%)		(-35.3%--28.8%)		(-28.4%-28.6%)		(-42.8%--33.4%)		(-33.8%-21.1%)
**Ireland**	2118.0 (1829.2-2398.1)	-34.1% (-37.0%--31.4%)	179.4 (155.4-205.3)	-34.0% (-36.9%--31.0%)	3.1 (1.5-6.1)	3.7% (-50.4%-38.1%)	8.0 (3.7-14.4)	-41.9% (-46.0%--37.7%)	38.4 (18.4-78.5)	0.3% (-55.9%-35.4%)
**Israel**	2050.4 (1789.6-2324.6)	-26.1% (-29.2%--23.2%)	177.2 (154.2-203.7)	-26.0% (-29.1%--23.2%)	2.5 (1.3-4.6)	18.5% (-12.6%-67.8%)	8.1 (3.8-14.8)	-34.2% (-38.5%--29.6%)	31.9 (15.4-59.0)	16.3% (-15.8%-61.0%)
**Italy**	1956.9 (1706.0-2207.2)	-37.2% (-38.0%--36.2%)	169.5 (146.8-192.4)	-34.0% (-34.9%--32.9%)	1.9 (0.9-3.7)	-7.3% (-44.6%-19.4%)	7.4 (3.5-13.6)	-42.8% (-44.2%--41.4%)	23.8 (11.3-47.4)	-11.4% (-51.7%-14.1%)
**Luxembourg**	2152.6 (1868.6-2437.8)	-27.7% (-30.8%--24.7%)	179.9 (156.3-206.9)	-27.7% (-30.8%--24.7%)	1.1 (0.5-2.2)	10.4% (-31.4%-45.1%)	8.1 (3.8-14.6)	-36.1% (-40.5%--30.9%)	13.8 (6.1-28.6)	5.9% (-36.0%-38.1%)
**Malta**	2245.0 (1945.1-2546.4)	-27.0% (-30.1%--23.4%)	191.7 (165.9-219.0)	-27.1% (-30.4%--23.6%)	2.1 (1.0-4.1)	13.2% (-21.8%-40.4%)	8.7 (4.1-16.0)	-33.9% (-38.4%--28.6%)	27.2 (12.8-53.3)	8.4% (-25.2%-37.0%)
**Monaco**	2009.9 (1745.1-2272.0)	-30.2% (-33.0%--27.1%)	163.2 (140.9-186.6)	-29.1% (-32.2%--25.9%)	0.7 (0.6-1.0)	14.3% (-13.5%-53.0%)	7.7 (3.6-14.1)	-35.0% (-39.6%--30.3%)	9.4 (7.1-11.9)	9.1% (-18.9%-49.6%)
**Netherlands**	1927.8 (1669.9-2191.0)	-32.4% (-37.0%--27.6%)	165.8 (144.2-191.3)	-31.5% (-36.1%--26.2%)	3.1 (1.5-5.7)	9.8% (-11.1%-26.8%)	7.2 (3.4-13.3)	-38.1% (-43.7%--32.2%)	38.9 (18.8-73.7)	6.3% (-13.6%-22.0%)
**Norway**	2048.2 (1786.7-2324.4)	-30.6% (-31.4%--29.7%)	174.0 (151.2-198.8)	-29.3% (-30.3%--28.3%)	1.4 (0.6-2.6)	3.8% (-47.0%-33.3%)	7.8 (3.7-14.1)	-36.9% (-38.2%--35.7%)	17.2 (8.0-33.5)	0.5% (-51.9%-28.9%)

**Portugal**	1944.2 (1688.6-2206.4)	-27.1% (-30.1%--23.0%)	170.1 (146.8-194.9)	-26.7% (-29.9%--22.4%)	2.5 (1.2-4.8)	6.7% (-28.1%-42.7%)	7.6 (3.5-13.8)	-36.8% (-41.6%--31.8%)	31.6 (14.9-62.0)	5.1% (-30.4%-39.8%)
**San Marino**	2005.8 (1740.4-2282.7)	-29.9% (-32.9%--26.9%)	169.1 (145.7-194.1)	-28.5% (-31.6%--25.0%)	1.0 (0.6-1.5)	25.9% (-20.6%-98.9%)	7.6 (3.6-13.8)	-35.8% (-40.6%--30.8%)	10.7 (6.5-16.6)	24.0% (-23.6%-101.9%)
**Spain**	1829.2 (1586.2-2077.3)	-30.9% (-33.8%--28.1%)	158.4 (137.5-182.2)	-30.2% (-33.2%--27.1%)	1.5 (0.7-2.8)	6.6% (-19.2%-34.8%)	6.8 (3.2-12.2)	-38.5% (-43.5%--34.0%)	18.7 (8.6-37.0)	2.7% (-20.5%-30.1%)
**Sweden**	2004.0 (1743.7-2272.2)	-30.0% (-32.5%--27.5%)	172.9 (149.6-198.7)	-28.5% (-31.0%--25.9%)	1.1 (0.5-2.0)	2.8% (-47.3%-41.0%)	8.1 (3.7-14.8)	-30.8% (-35.5%--26.1%)	12.5 (6.0-23.6)	0.9% (-49.7%-34.7%)
**Switzerland**	1966.0 (1707.6-2240.3)	-28.8% (-31.7%--25.9%)	166.5 (143.8-192.1)	-27.7% (-31.1%--24.8%)	1.4 (0.7-2.6)	2.4% (-37.2%-32.3%)	7.4 (3.5-13.5)	-34.1% (-39.1%--29.0%)	17.0 (8.1-32.7)	-1.9% (-40.4%-25.7%)
**United Kingdom**	1725.7 (1504.5-1956.4)	-40.4% (-41.0%--39.9%)	153.9 (133.5-175.6)	-38.2% (-38.9%--37.4%)	1.5 (0.6-2.6)	41.9% (-12.4%-510.2%)	6.7 (3.2-12.2)	-45.3% (-46.1%--44.5%)	18.0 (7.7-32.2)	32.5% (-18.7%-423.8%)
**Andean Latin America**	828.7 (715.4-951.2)	-3.8% (-6.9%--0.8%)	84.5 (73.1-96.9)	-4.7% (-7.7%--2.0%)	0.1 (0.1-0.2)	19.6% (-8.6%-58.1%)	4.1 (1.9-7.4)	-18.0% (-22.3%--13.3%)	1.8 (1.3-2.2)	18.8% (-8.9%-54.6%)
**Bolivia (Plurinational State of)**	861.1 (741.8-981.8)	-8.9% (-12.9%--4.7%)	90.1 (77.9-102.9)	-9.1% (-13.2%--4.5%)	0.2 (0.1-0.3)	56.9% (9.4%-122.2%)	4.6 (2.1-8.2)	-21.2% (-27.7%--14.4%)	2.6 (1.7-3.6)	46.4% (2.8%-104.9%)
**Ecuador**	866.8	-5.4%	87.9	-5.9%	0.2	100.4%	4.3	-19.2%	2.0	83.0%

	(745.5-999.2)	(-9.2%--1.1%)	(75.8-100.9)	(-9.7%--2.0%)	(0.1-0.2)	(48.2%-171.4%)	(2.0-7.6)	(-25.2%--12.4%)	(1.5-2.6)	(34.1%-148.8%)
**Peru**	802.7 (690.0-923.4)	-1.6% (-6.1%-3.2%)	81.3 (69.9-93.6)	-2.9% (-7.5%-1.4%)	0.1 (0.1-0.2)	-3.9% (-33.5%-44.0%)	3.9 (1.8-7.2)	-16.4% (-22.5%--9.2%)	1.5 (1.0-2.1)	-5.0% (-35.4%-42.4%)
**Caribbean**	985.7 (848.4-1129.5)	-5.9% (-7.8%--3.8%)	99.3 (85.8-114.2)	-6.3% (-8.0%--4.1%)	1.9 (1.0-3.4)	20.8% (-17.0%-48.0%)	4.7 (2.3-8.5)	-13.5% (-16.9%--10.3%)	28.1 (14.9-49.7)	19.1% (-17.7%-47.1%)
**Antigua and Barbuda**	969.3 (836.0-1107.9)	-5.7% (-9.6%--1.1%)	95.4 (82.1-109.8)	-6.1% (-10.5%--1.7%)	2.3 (1.1-4.4)	46.1% (5.9%-95.7%)	4.6 (2.2-8.5)	-13.2% (-20.0%--5.8%)	33.2 (15.7-62.5)	37.3% (0.1%-86.6%)
**Bahamas**	946.5 (814.2-1081.9)	-5.4% (-9.4%--0.8%)	93.2 (80.4-107.5)	-4.1% (-8.4%-0.3%)	2.8 (1.3-5.3)	21.5% (-11.7%-53.1%)	4.7 (2.2-8.5)	-10.8% (-17.9%--3.1%)	39.8 (19.1-75.1)	18.3% (-14.7%-51.5%)
**Barbados**	977.9 (844.6-1118.7)	-4.6% (-8.8%-0.2%)	97.3 (84.4-112.0)	-4.1% (-8.4%-1.0%)	7.1 (3.4-13.2)	23.2% (-11.8%-53.2%)	4.6 (2.2-8.3)	-12.2% (-18.5%--4.3%)	97.4 (46.6-176.1)	22.0% (-13.1%-55.8%)
**Belize**	962.2 (831.5-1103.3)	-2.9% (-6.7%-1.9%)	98.8 (85.3-113.9)	-3.8% (-7.8%-0.9%)	0.6 (0.3-1.1)	50.9% (-5.5%-106.5%)	4.9 (2.3-8.8)	-9.4% (-16.8%--1.6%)	8.5 (4.1-16.8)	49.4% (-4.9%-108.3%)
**Bermuda**	942.0 (813.5-1075.2)	-5.3% (-9.2%--1.1%)	88.5 (76.2-102.0)	-6.0% (-9.8%--1.9%)	3.7 (1.8-6.7)	0.1% (-34.6%-42.8%)	4.1 (1.9-7.5)	-16.9% (-22.8%--10.3%)	47.7 (23.4-89.5)	-4.8% (-40.8%-41.0%)
**Cuba**	991.0 (846.0-1139.0)	-10.6% (-14.2%--6.4%)	100.6 (86.1-115.8)	-9.9% (-13.6%--5.4%)	2.8 (1.3-5.1)	29.8% (-17.4%-78.7%)	4.5 (2.1-8.1)	-18.3% (-24.9%--11.5%)	41.8 (19.0-78.7)	27.6% (-18.6%-76.7%)

**Dominica**	932.6 (799.9-1067.6)	-5.7% (-9.7%--1.5%)	94.9 (81.7-110.0)	-6.1% (-10.0%--2.1%)	1.8 (1.4-2.3)	37.7% (5.0%-80.2%)	4.8 (2.3-8.5)	-8.7% (-15.9%--1.7%)	26.0 (20.4-32.8)	36.2% (3.6%-79.2%)
**Dominican Republic**	1008.3 (867.8-1156.7)	-2.1% (-6.6%-2.9%)	100.5 (87.0-116.3)	-4.7% (-8.8%--0.3%)	0.3 (0.2-0.5)	70.0% (26.3%-128.8%)	5.2 (2.5-9.3)	-12.6% (-19.9%--5.3%)	4.7 (3.3-6.3)	70.8% (24.0%-129.7%)
**Grenada**	1007.9 (866.7-1152.7)	-0.9% (-5.4%-4.0%)	100.9 (86.6-115.7)	-3.7% (-8.5%-0.6%)	3.0 (1.4-5.6)	53.1% (-6.8%-109.4%)	5.0 (2.4-9.1)	-8.9% (-15.6%--1.4%)	43.4 (20.3-80.1)	47.6% (-8.3%-94.2%)
**Guyana**	974.2 (843.1-1127.0)	-2.4% (-7.1%-2.6%)	101.8 (88.5-117.5)	-3.9% (-8.3%-0.8%)	1.1 (0.5-2.1)	50.9% (-3.5%-148.6%)	5.2 (2.4-9.2)	-8.9% (-16.3%--1.3%)	16.1 (7.4-31.7)	47.7% (-6.7%-145.3%)
**Haiti**	878.8 (754.9-1013.7)	-6.4% (-10.3%--2.1%)	95.4 (81.7-109.8)	-5.3% (-9.2%--0.9%)	1.2 (0.8-2.0)	0.8% (-32.0%-48.9%)	5.2 (2.4-9.2)	-13.8% (-20.6%--7.2%)	18.5 (11.8-29.8)	2.6% (-30.7%-46.5%)
**Jamaica**	1068.1 (921.2-1217.9)	7.5% (2.7%-12.4%)	108.0 (92.7-124.7)	6.4% (1.8%-11.1%)	2.2 (0.9-4.2)	40.5% (-19.8%-106.4%)	5.3 (2.5-9.6)	1.7% (-5.9%-10.5%)	32.9 (14.2-66.9)	45.0% (-19.9%-116.1%)
**Puerto Rico**	971.9 (836.1-1114.1)	-2.3% (-6.8%-1.7%)	93.1 (80.5-107.8)	-3.7% (-7.8%-0.6%)	1.8 (0.8-3.6)	23.8% (-21.1%-69.6%)	4.2 (2.0-7.6)	-12.1% (-18.9%--5.1%)	25.5 (11.6-51.1)	24.2% (-19.8%-72.6%)
**Saint Kitts and Nevis**	975.8 (839.8-1113.5)	-3.4% (-7.4%-1.0%)	96.9 (83.3-111.8)	-5.6% (-9.7%--0.8%)	5.1 (2.4-9.9)	30.0% (-24.1%-70.5%)	4.6 (2.1-8.1)	-11.8% (-18.8%--3.9%)	70.1 (32.9-135.0)	25.3% (-27.3%-71.3%)

**Saint Lucia**	1002.3 (864.3-1146.2)	-6.7% (-10.3%--2.6%)	101.0 (86.7-116.9)	-7.1% (-10.7%--3.0%)	3.9 (1.9-7.4)	20.4% (-27.7%-50.4%)	4.9 (2.3-8.7)	-15.1% (-21.4%--8.0%)	53.0 (25.5-101.5)	18.7% (-27.5%-52.3%)
**Saint Vincent and the Grenadines**	1005.1 (867.2-1147.8)	-1.4% (-5.7%-3.1%)	101.9 (87.8-116.4)	-2.7% (-7.2%-1.6%)	1.8 (0.8-3.3)	37.4% (-10.8%-98.4%)	5.1 (2.4-9.2)	-5.7% (-12.4%-1.7%)	25.0 (11.8-47.2)	37.4% (-12.0%-97.3%)
**Suriname**	1061.2 (910.0-1212.0)	2.7% (-1.6%-7.8%)	106.7 (92.0-122.4)	1.4% (-2.9%-6.8%)	0.2 (0.2-0.3)	27.7% (-3.0%-64.9%)	5.5 (2.6-9.9)	-4.7% (-11.9%-3.4%)	3.5 (2.8-4.4)	31.6% (-0.2%-73.0%)
**Trinidad and Tobago**	1083.1 (932.6-1243.3)	-7.2% (-11.4%--3.3%)	105.9 (91.5-123.0)	-9.0% (-13.1%--4.4%)	0.7 (0.3-1.3)	12.1% (-35.1%-72.0%)	5.4 (2.6-9.7)	-14.3% (-22.0%--6.3%)	10.2 (4.3-20.4)	15.5% (-33.0%-74.0%)
**United States Virgin Islands**	946.0 (818.5-1078.9)	-0.6% (-5.4%-4.0%)	90.6 (78.3-103.9)	-3.5% (-8.1%-0.7%)	3.6 (2.8-5.0)	49.1% (12.3%-94.7%)	4.5 (2.1-8.2)	-8.0% (-15.3%--0.4%)	51.5 (40.9-68.9)	44.6% (9.9%-89.0%)
**Central Latin America**	1038.9 (897.5-1190.4)	-15.4% (-16.6%--14.2%)	104.5 (90.2-120.4)	-14.6% (-15.9%--13.4%)	0.6 (0.3-1.2)	6.5% (-36.6%-46.1%)	5.0 (2.4-9.1)	-24.4% (-26.6%--21.4%)	7.9 (3.8-15.8)	5.5% (-37.9%-45.9%)
**Colombia**	971.5 (838.5-1113.2)	-18.1% (-21.9%--14.8%)	98.0 (84.2-113.1)	-18.3% (-22.1%--14.7%)	0.5 (0.2-1.1)	11.4% (-40.9%-68.3%)	4.6 (2.1-8.1)	-28.4% (-33.9%--22.3%)	7.2 (3.2-15.2)	7.4% (-44.0%-69.5%)
**Costa Rica**	999.9	-12.7%	99.3 (85.3-114.1)	-13.6%	0.4 (0.2-0.7)	33.3%	4.6 (2.1-8.1)	-19.0%	5.1 (2.3-10.1)	31.7%

	(854.8-1146.7)	(-16.9%--8.7%)		(-17.5%--9.5%)		(-12.5%-79.3%)		(-25.6%--12.1%)		(-14.4%-79.4%)
**El Salvador**	971.5 (835.5-1119.2)	-2.9% (-7.5%-2.2%)	99.9 (85.8-115.1)	-4.3% (-8.9%-1.2%)	0.1 (0.1-0.1)	38.9% (1.2%-87.3%)	4.7 (2.2-8.5)	-16.3% (-23.4%--8.4%)	1.3 (0.8-1.7)	26.4% (-8.4%-70.0%)
**Guatemala**	978.0 (842.8-1122.8)	0.3% (-4.2%-4.7%)	102.0 (87.6-117.8)	-1.0% (-5.2%-3.8%)	0.1 (0.0-0.2)	24.4% (-25.7%-97.3%)	5.1 (2.4-9.4)	-12.1% (-18.9%--4.7%)	1.3 (0.6-2.6)	20.7% (-26.5%-87.8%)
**Honduras**	1027.6 (886.6-1182.1)	-6.7% (-11.0%--1.6%)	107.8 (93.2-124.3)	-6.8% (-11.2%--1.3%)	0.5 (0.3-0.7)	24.3% (-13.1%-87.3%)	5.4 (2.5-9.6)	-16.0% (-22.9%--8.6%)	6.0 (3.6-8.4)	22.3% (-13.8%-80.3%)
**Mexico**	1087.4 (940.4-1246.9)	-15.2% (-16.3%--13.9%)	108.3 (93.5-124.6)	-14.4% (-15.6%--13.3%)	0.7 (0.3-1.5)	1.6% (-42.3%-46.3%)	5.3 (2.5-9.5)	-23.3% (-25.7%--19.9%)	9.8 (4.5-20.2)	2.1% (-41.6%-47.2%)
**Nicaragua**	1029.2 (886.2-1177.4)	-8.6% (-12.5%--4.3%)	105.6 (91.1-121.4)	-8.0% (-12.2%--3.7%)	0.2 (0.1-0.2)	100.9% (56.7%-156.1%)	5.0 (2.3-9.1)	-20.2% (-26.4%--13.2%)	2.3 (1.8-2.8)	77.2% (38.6%-126.0%)
**Panama**	968.6 (831.8-1110.1)	-8.1% (-12.0%--4.4%)	95.0 (81.7-110.1)	-10.3% (-14.2%--6.7%)	1.1 (0.5-2.1)	29.5% (-31.5%-85.6%)	4.6 (2.2-8.3)	-16.8% (-23.0%--10.4%)	14.1 (6.3-28.6)	29.7% (-33.6%-87.8%)
**Venezuela (Bolivarian Republic of)**	1031.6 (883.1-1184.9)	-20.8% (-24.2%--16.5%)	104.5 (89.8-120.7)	-18.8% (-22.7%--14.0%)	0.5 (0.2-0.9)	14.6% (-22.9%-54.7%)	5.0 (2.4-8.9)	-29.1% (-35.1%--22.5%)	6.2 (2.9-12.5)	13.5% (-25.0%-57.8%)

**Tropical Latin America**	941.6 (817.7-1072.8)	-20.7% (-21.9%--19.6%)	95.0 (82.1-108.8)	-19.8% (-21.0%--18.6%)	1.2 (0.6-2.3)	15.5% (-16.7%-52.1%)	4.6 (2.2-8.4)	-28.5% (-31.0%--25.3%)	18.7 (8.6-35.3)	11.2% (-18.9%-46.7%)
**Brazil**	938.7 (815.1-1068.9)	-21.1% (-22.2%--19.9%)	94.7 (81.9-108.4)	-20.1% (-21.3%--18.9%)	1.3 (0.6-2.3)	13.9% (-18.4%-50.1%)	4.6 (2.2-8.3)	-28.9% (-31.3%--25.6%)	19.0 (8.7-35.9)	10.2% (-20.3%-45.1%)
**Paraguay**	1068.4 (919.2-1232.4)	-5.3% (-9.5%--0.8%)	107.5 (92.8-124.3)	-6.0% (-10.3%--1.3%)	0.5 (0.3-0.6)	160.9% (76.4%-263.9%)	5.3 (2.5-9.6)	-14.6% (-20.9%--7.3%)	6.8 (4.8-9.1)	170.5% (85.2%-277.5%)
**North Africa and Middle East**	1115.6 (966.1-1276.4)	-1.3% (-2.6%-0.1%)	109.7 (95.0-125.8)	-1.6% (-2.9%--0.3%)	0.4 (0.3-0.6)	28.7% (-2.3%-66.2%)	5.2 (2.5-9.4)	-15.3% (-17.7%--11.7%)	6.0 (4.9-7.8)	18.2% (-12.7%-51.6%)
**Afghanistan**	1057.5 (911.8-1214.2)	10.3% (5.4%-15.4%)	111.6 (96.9-127.0)	10.4% (5.4%-15.5%)	0.1 (0.1-0.2)	66.2% (15.0%-141.1%)	6.0 (2.8-10.7)	1.9% (-5.9%-10.3%)	1.9 (1.2-2.9)	64.2% (9.4%-139.9%)
**Algeria**	1117.6 (957.5-1279.1)	-1.3% (-5.4%-3.2%)	109.9 (94.9-125.8)	-1.3% (-5.4%-2.8%)	0.3 (0.2-0.5)	57.8% (11.7%-130.2%)	5.2 (2.4-9.4)	-13.4% (-20.5%--5.8%)	4.4 (2.7-6.7)	55.8% (9.1%-126.7%)
**Bahrain**	1185.5 (1030.1-1351.3)	-2.6% (-7.0%-1.5%)	110.1 (95.0-126.8)	-2.3% (-6.5%-2.1%)	1.5 (1.1-1.9)	26.2% (-12.2%-76.4%)	5.2 (2.4-9.4)	-17.0% (-23.5%--10.2%)	21.0 (15.8-27.0)	12.2% (-24.0%-58.8%)
**Egypt**	1098.2 (943.3-1270.7)	7.6% (3.1%-12.8%)	111.2 (95.7-128.8)	6.2% (1.6%-11.2%)	0.2 (0.1-0.3)	101.6% (43.3%-186.0%)	5.4 (2.5-9.6)	-5.9% (-13.0%-1.8%)	2.7 (1.8-3.9)	104.7% (43.1%-188.5%)

**Iran (Islamic Republic of)**	1139.5 (989.9-1301.2)	5.8% (4.6%-7.1%)	110.7 (95.9-127.0)	4.9% (3.7%-6.2%)	0.3 (0.2-0.3)	153.3% (45.0%-280.4%)	5.1 (2.5-9.3)	-6.9% (-9.5%--3.2%)	4.1 (3.0-4.7)	162.8% (58.5%-280.5%)
**Iraq**	1233.9 (1061.9-1416.8)	-11.1% (-15.1%--7.5%)	121.3 (105.1-139.7)	-9.6% (-13.7%--5.7%)	0.1 (0.1-0.2)	20.3% (-17.4%-86.9%)	5.8 (2.7-10.6)	-20.7% (-26.7%--14.0%)	1.9 (1.5-2.9)	12.9% (-25.9%-77.0%)
**Jordan**	1191.4 (1029.6-1367.4)	-2.0% (-5.7%-2.3%)	117.1 (100.9-134.6)	-1.9% (-6.0%-2.9%)	0.1 (0.1-0.1)	20.5% (-13.3%-60.1%)	5.4 (2.5-9.8)	-15.2% (-21.1%--7.4%)	1.4 (1.1-1.8)	15.4% (-15.7%-52.6%)
**Kuwait**	1119.9 (964.7-1278.0)	2.1% (-1.8%-6.0%)	102.6 (88.5-118.1)	2.7% (-1.5%-7.2%)	0.1 (0.1-0.2)	54.7% (-4.5%-127.3%)	4.8 (2.2-8.6)	-8.5% (-15.2%--1.9%)	1.5 (0.7-3.1)	48.4% (-9.8%-112.7%)
**Lebanon**	1364.4 (1173.5-1570.3)	3.8% (-0.5%-8.1%)	130.3 (112.6-151.0)	2.9% (-1.9%-7.3%)	0.3 (0.2-0.3)	75.7% (7.1%-175.7%)	5.7 (2.7-10.5)	-14.5% (-20.8%--7.6%)	3.4 (2.3-4.5)	74.3% (7.0%-172.8%)
**Libya**	1113.0 (963.7-1277.6)	3.1% (-1.1%-7.5%)	109.6 (94.9-125.6)	6.6% (2.1%-11.0%)	0.2 (0.1-0.2)	98.4% (25.4%-209.8%)	5.2 (2.4-9.5)	-6.8% (-13.8%-0.2%)	2.3 (1.4-3.2)	102.1% (27.3%-218.7%)
**Morocco**	1082.0 (936.2-1239.1)	-0.6% (-4.5%-3.5%)	108.9 (94.1-125.2)	-1.6% (-5.7%-2.8%)	0.2 (0.2-0.3)	133.6% (61.2%-223.8%)	5.4 (2.5-9.8)	-13.0% (-19.1%--5.0%)	3.1 (2.1-4.2)	124.6% (56.6%-207.7%)
**Oman**	1171.0 (1007.0-1335.1)	8.9% (4.5%-13.7%)	110.5 (94.9-127.3)	8.3% (4.1%-13.5%)	0.4 (0.2-0.6)	126.9% (54.9%-253.7%)	5.1 (2.4-9.3)	-7.0% (-13.4%--0.1%)	5.8 (3.2-8.4)	110.1% (40.7%-229.7%)

**Palestine**	1082.9 (937.9-1244.8)	0.8% (-3.7%-5.3%)	109.4 (94.9-124.7)	-0.5% (-4.9%-4.0%)	0.1 (0.1-0.1)	35.3% (-6.4%-119.3%)	5.1 (2.4-8.9)	-8.2% (-14.9%--0.8%)	1.3 (1.0-1.6)	29.8% (-9.2%-100.9%)
**Qatar**	1074.1 (930.3-1221.9)	-15.1% (-19.0%--11.4%)	97.6 (84.6-112.5)	-12.2% (-16.1%--8.3%)	1.3 (0.8-1.8)	136.1% (53.8%-311.2%)	4.6 (2.2-8.3)	-29.3% (-34.9%--23.5%)	13.1 (8.3-18.3)	86.2% (20.3%-236.5%)
**Saudi Arabia**	978.5 (844.5-1111.6)	1.2% (-3.5%-5.2%)	93.1 (80.4-107.5)	1.6% (-3.5%-5.7%)	0.1 (0.1-0.1)	43.0% (-8.8%-164.2%)	4.3 (2.0-7.8)	-14.7% (-21.4%--7.7%)	1.3 (1.0-1.6)	32.7% (-13.5%-139.2%)
**Sudan**	918.3 (793.6-1047.5)	6.7% (2.0%-11.4%)	95.8 (82.5-109.3)	5.7% (0.7%-10.1%)	0.2 (0.1-0.3)	103.7% (36.7%-199.1%)	4.8 (2.2-8.6)	-5.2% (-12.9%-3.0%)	2.6 (1.6-3.9)	98.7% (35.0%-192.0%)
**Syrian Arab Republic**	1044.8 (899.5-1195.2)	-5.4% (-9.2%--1.2%)	107.3 (92.7-123.3)	-4.4% (-8.0%-0.0%)	0.1 (0.1-0.1)	22.3% (-14.2%-77.4%)	4.8 (2.3-8.6)	-18.3% (-24.0%--11.4%)	1.1 (0.8-1.4)	17.9% (-16.8%-69.5%)
**Tunisia**	1164.6 (1007.2-1327.4)	3.1% (-1.8%-8.3%)	113.2 (98.6-129.8)	1.3% (-3.5%-6.0%)	0.2 (0.2-0.3)	90.8% (30.8%-169.1%)	5.2 (2.5-9.6)	-10.8% (-18.0%--3.1%)	3.0 (2.0-4.1)	90.5% (29.9%-172.2%)
**Turkey**	1149.4 (994.1-1313.7)	-14.2% (-17.9%--10.3%)	110.0 (95.5-126.1)	-14.6% (-18.3%--10.5%)	1.0 (0.7-1.6)	-0.7% (-31.7%-39.1%)	5.1 (2.4-9.4)	-30.0% (-35.5%--23.6%)	15.9 (12.1-23.1)	-9.3% (-38.6%-27.2%)
**United Arab Emirates**	1062.5 (915.3-1220.1)	-3.1% (-7.5%-1.0%)	99.5 (86.0-115.2)	1.4% (-3.2%-6.2%)	0.3 (0.2-0.7)	63.3% (-5.2%-185.3%)	5.1 (2.4-9.3)	-12.6% (-19.1%--5.9%)	5.2 (2.4-9.9)	65.1% (-6.2%-195.9%)

**Yemen**	1023.0 (885.9-1164.3)	2.3% (-2.1%-7.2%)	107.2 (92.6-123.4)	3.7% (-0.4%-8.9%)	0.2 (0.1-0.2)	83.3% (17.3%-180.2%)	5.4 (2.5-9.7)	-6.8% (-13.7%-0.6%)	2.2 (1.4-3.4)	76.1% (13.8%-166.5%)
**South Asia**	917.3 (797.7-1047.2)	-0.1% (-1.1%-0.8%)	93.1 (80.5-106.5)	-0.6% (-1.6%-0.3%)	0.3 (0.2-0.4)	71.1% (11.6%-114.9%)	4.8 (2.3-8.4)	-10.8% (-13.2%--7.4%)	4.2 (3.1-5.3)	67.9% (8.9%-113.3%)
**Bangladesh**	819.9 (708.8-939.1)	-3.0% (-7.1%-1.2%)	85.1 (73.5-97.5)	-2.7% (-6.5%-1.5%)	0.3 (0.2-0.5)	126.0% (29.7%-248.9%)	4.2 (2.0-7.5)	-16.8% (-23.5%--8.9%)	4.6 (2.4-6.8)	113.2% (21.6%-233.4%)
**Bhutan**	853.5 (736.3-971.8)	-3.9% (-8.1%-0.5%)	85.8 (74.1-98.4)	-4.0% (-8.3%-0.6%)	0.4 (0.3-0.6)	152.1% (38.9%-286.1%)	4.3 (2.0-7.7)	-19.6% (-26.1%--11.8%)	5.9 (3.5-9.0)	138.1% (30.6%-262.9%)
**India**	918.9 (797.5-1050.7)	0.9% (-0.1%-1.9%)	92.9 (80.3-106.4)	-0.3% (-1.3%-0.7%)	0.3 (0.2-0.4)	70.3% (5.7%-118.0%)	4.8 (2.3-8.4)	-9.2% (-11.8%--5.7%)	4.0 (3.0-5.1)	66.1% (3.3%-117.0%)
**Nepal**	899.0 (777.8-1033.4)	-3.2% (-7.1%-1.0%)	93.5 (80.6-107.4)	-2.3% (-6.4%-2.3%)	0.3 (0.2-0.4)	145.7% (51.7%-262.7%)	4.8 (2.3-8.4)	-15.4% (-22.2%--7.2%)	4.3 (2.7-6.0)	128.2% (37.1%-234.2%)
**Pakistan**	1032.9 (893.3-1180.7)	1.6% (-1.2%-4.6%)	104.6 (90.5-119.6)	2.3% (-0.5%-5.2%)	0.4 (0.3-0.5)	71.4% (19.3%-130.0%)	5.7 (2.7-10.2)	-5.8% (-10.5%--0.7%)	5.4 (3.6-7.7)	69.5% (16.0%-132.0%)
**East Asia**	1426.7 (1238.8-1627.0)	6.8% (5.5%-8.1%)	125.6 (109.3-143.5)	3.2% (2.0%-4.4%)	0.1 (0.1-0.2)	21.1% (-13.7%-51.8%)	6.9 (3.2-12.5)	-13.4% (-17.8%--8.4%)	1.9 (1.6-2.4)	15.3% (-18.4%-46.5%)
**China**	1423.8	7.0% (5.7%-8.3%)	125.4 (109.1-143.4)	3.3% (2.0%-4.6%)	0.1 (0.1-0.2)	21.2%	6.9 (3.2-12.5)	-13.6%	2.0 (1.6-2.4)	15.4%

	(1234.8-1625.3)					(-14.3%-52.3%)		(-18.1%--8.4%)		(-19.3%-46.8%)
**Democratic People's Republic of Korea**	1403.8 (1210.9-1614.9)	3.1% (-1.8%-7.9%)	132.6 (115.0-151.1)	5.4% (0.9%-10.1%)	0.1 (0.1-0.1)	6.9% (-22.1%-50.2%)	7.8 (3.6-14.3)	0.9% (-7.4%-9.8%)	1.7 (1.3-2.2)	8.5% (-21.7%-57.1%)
**Taiwan (Province of China)**	1635.9 (1445.7-1810.8)	2.4% (-4.4%-11.6%)	132.2 (117.2-147.1)	-1.2% (-7.5%-6.8%)	0.1 (0.0-0.2)	26.0% (-18.3%-84.1%)	7.2 (3.3-12.9)	-12.2% (-20.9%--2.1%)	1.3 (0.6-2.8)	19.2% (-22.8%-78.3%)
**Oceania**	1450.5 (1258.7-1653.5)	9.3% (6.6%-12.9%)	139.2 (120.9-159.1)	8.9% (6.0%-12.1%)	0.2 (0.2-0.3)	47.8% (10.3%-92.8%)	8.5 (4.0-15.5)	4.7% (-0.4%-10.4%)	3.1 (2.4-4.6)	49.0% (13.7%-95.2%)
**American Samoa**	1695.9 (1466.5-1935.0)	10.4% (5.8%-15.0%)	152.9 (132.1-176.0)	13.3% (8.8%-17.7%)	0.3 (0.3-0.4)	61.7% (18.0%-116.1%)	9.1 (4.2-16.1)	4.4% (-3.0%-12.6%)	5.6 (4.5-7.0)	58.0% (15.9%-113.0%)
**Cook Islands**	1590.7 (1374.8-1815.4)	13.4% (8.3%-18.2%)	140.6 (121.4-161.4)	11.4% (6.7%-16.3%)	0.4 (0.3-0.5)	57.7% (2.9%-128.7%)	7.7 (3.6-14.0)	4.8% (-2.4%-12.8%)	6.8 (4.8-9.0)	52.7% (-1.3%-129.4%)
**Fiji**	1774.9 (1535.1-2020.3)	10.5% (6.2%-14.8%)	164.0 (142.5-187.4)	9.1% (5.1%-13.5%)	0.2 (0.2-0.3)	66.4% (-0.6%-155.3%)	10.0 (4.7-17.8)	5.7% (-1.8%-13.5%)	3.3 (2.2-4.3)	55.5% (-5.0%-134.0%)
**Guam**	1437.8	16.8%	124.1 (107.4-142.6)	16.9%	0.2 (0.2-0.3)	16.6%	7.5 (3.5-13.6)	13.9%	3.9 (3.0-4.9)	47.8%

	(1239.0-1638.4)	(12.2%-22.1%)		(12.7%-21.9%)		(-22.7%-62.8%)		(5.4%-22.9%)		(-4.6%-109.2%)
**Kiribati**	2009.4 (1734.3-2306.4)	28.6% (23.1%-34.7%)	192.4 (166.6-220.9)	27.5% (22.4%-33.7%)	0.2 (0.1-0.5)	15.9% (-15.2%-60.8%)	12.5 (5.9-23.0)	23.4% (13.2%-34.7%)	3.3 (2.3-6.6)	8.4% (-23.2%-50.5%)
**Marshall Islands**	1467.4 (1269.2-1670.8)	9.1% (4.6%-13.7%)	143.2 (124.4-164.2)	9.8% (5.1%-14.9%)	0.3 (0.2-0.5)	48.8% (10.3%-109.5%)	8.5 (4.1-15.2)	2.6% (-5.1%-11.4%)	5.6 (3.9-7.9)	44.0% (3.2%-107.6%)
**Micronesia (Federated States of)**	1498.4 (1294.4-1722.9)	10.3% (5.2%-15.2%)	145.5 (125.7-166.8)	10.2% (5.6%-14.7%)	0.4 (0.3-0.5)	70.1% (17.7%-142.8%)	8.6 (4.0-15.5)	0.9% (-6.8%-9.5%)	6.2 (4.3-9.0)	67.4% (7.9%-146.1%)
**Nauru**	1569.7 (1350.0-1788.5)	12.1% (7.3%-17.3%)	148.6 (127.9-169.9)	16.8% (12.1%-22.0%)	0.4 (0.3-0.5)	17.5% (-13.3%-54.7%)	8.5 (3.9-15.0)	5.4% (-2.8%-14.3%)	6.2 (4.4-8.7)	18.3% (-14.5%-59.0%)
**Niue**	1589.9 (1376.4-1814.8)	11.3% (6.3%-15.9%)	146.2 (127.2-167.7)	10.9% (6.4%-16.0%)	0.4 (0.3-0.5)	52.3% (10.3%-111.3%)	8.0 (3.8-14.4)	1.3% (-6.4%-9.7%)	6.1 (4.6-8.0)	54.8% (9.9%-118.7%)
**Northern Mariana Islands**	1410.8 (1225.4-1602.0)	9.8% (5.6%-14.5%)	126.6 (109.1-145.0)	14.0% (9.9%-18.9%)	0.6 (0.4-0.8)	65.5% (13.6%-138.5%)	7.0 (3.2-12.6)	3.4% (-4.2%-12.3%)	7.7 (5.4-9.6)	60.8% (11.2%-128.2%)
**Palau**	1567.0 (1353.4-1782.9)	11.9% (7.5%-17.1%)	143.9 (125.1-164.7)	11.9% (7.2%-17.2%)	0.1 (0.1-0.1)	19.7% (-21.2%-81.8%)	7.9 (3.7-14.2)	4.8% (-3.0%-13.8%)	1.4 (1.0-2.0)	16.8% (-29.7%-94.3%)

**Papua New Guinea**	1356.0 (1173.6-1550.6)	9.2% (4.8%-14.4%)	132.7 (115.3-152.4)	8.8% (4.4%-13.7%)	0.2 (0.1-0.3)	51.0% (7.1%-115.8%)	8.2 (3.9-14.9)	4.8% (-2.9%-13.2%)	2.7 (1.8-4.7)	55.4% (11.1%-125.2%)
**Samoa**	1783.9 (1542.9-2044.5)	13.7% (8.8%-18.3%)	166.8 (144.1-192.4)	12.9% (8.0%-18.1%)	0.3 (0.2-0.4)	28.1% (-5.1%-79.3%)	9.8 (4.5-17.4)	7.0% (-0.7%-15.2%)	4.8 (3.5-6.5)	26.9% (-10.1%-81.9%)
**Solomon Islands**	1462.7 (1269.7-1676.4)	12.9% (8.3%-17.9%)	145.2 (126.0-165.7)	12.7% (8.1%-17.4%)	0.2 (0.2-0.3)	48.4% (8.2%-102.4%)	8.5 (4.0-15.1)	6.1% (-1.7%-14.6%)	3.7 (2.5-5.7)	44.3% (2.9%-103.6%)
**Tokelau**	1554.6 (1339.8-1788.2)	10.9% (6.2%-15.6%)	146.4 (126.5-167.7)	8.3% (4.0%-12.7%)	0.3 (0.2-0.4)	66.6% (17.0%-136.2%)	8.3 (3.8-14.7)	-0.3% (-7.9%-8.4%)	4.5 (3.1-6.0)	67.1% (17.7%-140.5%)
**Tonga**	1695.2 (1460.3-1944.2)	10.9% (5.9%-15.2%)	158.7 (136.3-181.9)	10.1% (5.5%-14.4%)	0.3 (0.2-0.4)	51.2% (12.3%-108.1%)	9.4 (4.4-17.0)	5.8% (-1.5%-13.9%)	4.5 (3.2-6.1)	57.8% (13.7%-123.4%)
**Tuvalu**	1563.8 (1352.9-1791.4)	10.3% (5.1%-15.2%)	151.3 (130.0-172.9)	9.5% (4.3%-13.9%)	0.3 (0.2-0.4)	51.2% (3.2%-119.9%)	8.8 (4.1-15.7)	0.8% (-7.1%-9.3%)	4.4 (3.2-6.4)	46.6% (-2.3%-113.8%)
**Vanuatu**	1547.3 (1337.0-1764.1)	13.3% (8.7%-18.7%)	151.1 (131.2-173.5)	13.2% (7.6%-18.6%)	0.2 (0.2-0.4)	87.9% (22.2%-182.9%)	9.3 (4.5-16.5)	9.4% (1.2%-17.6%)	3.8 (2.6-5.9)	91.9% (21.4%-202.3%)
**Southeast Asia**	1511.7 (1314.0-1725.4)	4.2% (2.8%-5.7%)	140.6 (122.2-161.0)	2.1% (0.8%-3.3%)	0.2 (0.2-0.2)	52.9% (23.5%-80.5%)	8.1 (3.8-14.7)	-6.4% (-9.4%--2.6%)	3.0 (2.3-3.7)	52.7% (22.4%-82.0%)
**Cambodia**	1432.9	-2.6% (-7.4%-1.7%)	139.3 (121.0-160.0)	-3.7% (-8.2%-0.7%)	0.2 (0.1-0.2)	72.7%	8.1 (3.8-14.4)	-14.3%	2.4 (1.6-3.5)	64.6%

	(1240.1-1630.6)					(13.1%-147.6%)		(-21.6%--5.5%)		(7.1%-143.7%)
**Indonesia**	1640.1 (1423.8-1871.0)	12.2% (10.2%-14.2%)	151.3 (131.9-172.7)	9.2% (7.3%-11.1%)	0.3 (0.2-0.4)	90.2% (47.4%-140.5%)	9.2 (4.3-16.8)	4.1% (0.0%-9.6%)	3.8 (2.5-5.5)	81.2% (36.9%-131.3%)
**Lao People's Democratic Republic**	1464.4 (1268.9-1669.9)	-3.0% (-7.2%-1.5%)	139.9 (121.1-160.9)	-4.3% (-8.4%-0.2%)	0.2 (0.1-0.3)	63.5% (15.7%-134.3%)	8.7 (4.1-15.8)	-13.3% (-20.6%--3.6%)	3.1 (2.1-4.1)	59.7% (8.2%-143.6%)
**Malaysia**	1428.6 (1239.9-1623.5)	-2.5% (-6.4%-2.0%)	128.5 (112.0-147.5)	-3.8% (-7.5%-1.0%)	0.2 (0.1-0.2)	80.1% (30.9%-144.8%)	7.2 (3.4-12.9)	-14.0% (-20.4%--6.9%)	2.6 (1.9-3.3)	69.9% (22.2%-132.2%)
**Maldives**	1410.2 (1213.8-1607.2)	2.9% (-2.0%-7.4%)	129.2 (111.6-148.2)	0.0% (-4.7%-4.2%)	0.4 (0.3-0.7)	76.2% (-22.8%-178.5%)	6.9 (3.2-12.4)	-14.9% (-21.8%--6.0%)	5.4 (3.9-8.7)	47.2% (-37.2%-145.1%)
**Mauritius**	1679.0 (1448.0-1928.6)	7.6% (3.2%-12.0%)	149.3 (129.6-170.9)	5.4% (1.2%-9.4%)	0.3 (0.1-0.6)	66.2% (5.0%-152.2%)	8.4 (4.0-15.4)	-0.5% (-8.5%-7.4%)	4.3 (2.0-8.9)	61.0% (0.7%-144.0%)
**Myanmar**	1678.4 (1454.3-1914.2)	-6.6% (-11.3%--2.0%)	158.5 (138.2-180.1)	-8.6% (-13.4%--4.2%)	0.2 (0.2-0.3)	47.6% (4.6%-105.4%)	9.6 (4.6-17.5)	-16.7% (-24.5%--7.9%)	3.3 (2.5-4.5)	38.1% (-6.7%-99.3%)
**Philippines**	1513.0 (1314.5-1724.1)	8.3% (7.4%-9.1%)	140.9 (122.9-161.4)	6.7% (5.9%-7.5%)	0.2 (0.1-0.2)	46.4% (18.4%-75.4%)	8.6 (4.0-15.6)	4.2% (2.1%-6.6%)	3.0 (2.4-3.7)	63.6% (25.5%-104.4%)

**Seychelles**	1515.7 (1312.9-1735.4)	5.6% (1.2%-10.4%)	133.6 (116.2-153.1)	4.6% (0.3%-8.7%)	0.3 (0.2-0.5)	31.0% (-4.1%-79.2%)	7.9 (3.7-14.1)	-4.8% (-12.3%-3.2%)	5.2 (3.1-7.4)	28.2% (-4.9%-68.8%)
**Sri Lanka**	1464.3 (1267.8-1671.0)	8.9% (4.7%-14.6%)	133.8 (117.1-153.5)	5.5% (1.4%-10.5%)	0.1 (0.1-0.2)	55.7% (-9.5%-140.2%)	7.2 (3.3-13.0)	-5.0% (-11.8%-3.3%)	2.3 (1.5-3.1)	51.1% (-19.7%-144.1%)
**Thailand**	1269.0 (1094.5-1449.2)	-9.6% (-13.5%--5.4%)	116.2 (100.1-134.4)	-10.1% (-14.2%--5.7%)	0.1 (0.1-0.1)	11.9% (-22.5%-55.9%)	6.3 (3.0-11.4)	-20.8% (-27.6%--13.6%)	1.0 (0.8-1.4)	7.8% (-24.4%-52.9%)
**Timor-Leste**	1425.5 (1231.5-1632.6)	4.7% (-0.1%-9.9%)	136.7 (118.5-156.2)	3.8% (-1.0%-9.1%)	0.2 (0.1-0.3)	95.7% (34.0%-201.8%)	8.2 (3.8-14.7)	-6.3% (-14.1%-2.4%)	2.7 (1.6-4.0)	93.7% (28.5%-198.4%)
**Viet Nam**	1487.3 (1284.3-1694.0)	11.2% (6.5%-16.9%)	139.5 (120.8-159.6)	7.6% (2.9%-13.0%)	0.3 (0.2-0.3)	55.3% (4.3%-119.9%)	7.7 (3.5-13.6)	-4.4% (-11.5%-4.4%)	3.9 (2.8-5.1)	51.1% (-2.6%-124.6%)
**Central Sub-Saharan Africa**	1073.5 (924.1-1224.6)	-0.6% (-3.9%-2.8%)	111.4 (96.6-127.6)	0.1% (-3.1%-3.5%)	2.2 (1.1-3.5)	-6.9% (-33.8%-57.9%)	6.3 (3.0-11.4)	-6.0% (-11.3%--0.3%)	36.8 (18.9-58.6)	-6.6% (-34.8%-57.1%)
**Angola**	1209.7 (1044.2-1381.0)	6.4% (1.4%-11.3%)	119.7 (103.4-137.7)	4.7% (-0.3%-9.1%)	1.6 (0.8-2.2)	52.5% (4.6%-124.9%)	7.0 (3.3-12.6)	-4.1% (-11.7%-3.8%)	26.6 (14.6-36.6)	44.0% (-2.6%-120.2%)
**Central African Republic**	1116.2 (963.6-1283.6)	1.3% (-3.1%-6.8%)	118.3 (102.7-135.3)	3.0% (-1.5%-8.0%)	1.3 (0.6-2.0)	3.6% (-24.3%-43.1%)	7.1 (3.4-12.8)	1.5% (-7.3%-9.9%)	22.2 (11.8-34.6)	1.6% (-27.6%-42.5%)

**Congo**	1132.7 (973.4-1301.4)	-5.7% (-9.8%--1.4%)	113.8 (98.4-131.2)	-4.1% (-8.1%-0.2%)	2.6 (1.5-3.6)	27.6% (-9.7%-86.9%)	6.5 (3.0-11.7)	-12.5% (-19.7%--5.2%)	41.5 (25.5-58.2)	21.3% (-17.6%-79.9%)
**Democratic Republic of the Congo**	1021.9 (880.5-1163.8)	-2.7% (-7.1%-2.1%)	108.2 (93.5-124.4)	-1.5% (-5.8%-3.3%)	2.3 (1.0-4.0)	-18.0% (-43.9%-47.9%)	6.0 (2.9-10.7)	-6.3% (-13.4%-1.4%)	39.8 (17.8-69.1)	-15.1% (-43.3%-50.0%)
**Equatorial Guinea**	1186.9 (1025.7-1355.1)	6.0% (0.1%-11.9%)	109.9 (94.9-126.2)	-5.1% (-10.1%-0.1%)	2.9 (1.2-4.7)	142.6% (39.0%-364.1%)	6.4 (3.0-11.3)	-11.2% (-19.0%--2.6%)	43.6 (18.7-73.8)	110.3% (16.2%-302.1%)
**Gabon**	1175.2 (1014.8-1343.9)	0.9% (-3.3%-5.8%)	110.8 (95.5-127.8)	2.7% (-1.4%-7.7%)	3.1 (1.7-4.3)	53.1% (7.4%-136.6%)	6.4 (3.0-11.6)	-7.2% (-14.0%-1.0%)	50.8 (29.9-72.5)	45.9% (-2.2%-131.6%)
**Eastern Sub-Saharan Africa**	939.1 (813.8-1076.6)	2.4% (1.2%-3.8%)	97.8 (84.4-111.9)	2.0% (0.8%-3.4%)	1.4 (0.7-1.9)	40.7% (8.9%-77.3%)	5.4 (2.5-9.6)	-4.7% (-7.3%--1.5%)	22.2 (12.0-29.5)	35.9% (2.1%-73.0%)
**Burundi**	918.5 (800.2-1050.9)	-2.5% (-7.5%-2.4%)	98.3 (84.9-112.4)	-1.5% (-6.4%-3.3%)	1.1 (0.5-1.6)	-2.5% (-35.8%-44.8%)	5.5 (2.5-9.7)	-8.3% (-16.1%--0.2%)	17.1 (8.6-25.6)	-6.4% (-40.9%-40.4%)
**Comoros**	945.2 (815.7-1088.5)	0.1% (-4.3%-4.5%)	98.8 (85.1-114.1)	1.5% (-2.8%-5.9%)	1.3 (0.6-1.9)	18.2% (-20.1%-74.0%)	5.4 (2.6-9.6)	-8.5% (-15.2%--0.8%)	19.7 (9.2-29.9)	13.3% (-27.7%-76.9%)
**Djibouti**	980.0	2.4% (-1.8%-6.8%)	99.4 (86.0-113.6)	3.6% (-0.7%-7.9%)	1.7 (0.7-2.6)	40.4%	5.5 (2.6-10.0)	-5.8%	27.1 (12.7-41.1)	35.5%

	(846.8-1120.9)					(-2.3%-105.6%)		(-13.0%-2.5%)		(-8.7%-102.5%)
**Eritrea**	910.5 (787.3-1040.7)	-0.5% (-5.2%-4.2%)	95.8 (82.6-109.2)	-1.0% (-5.5%-3.6%)	1.2 (0.5-1.9)	56.2% (-0.4%-158.5%)	5.4 (2.5-9.8)	-9.8% (-17.5%--0.8%)	18.8 (8.4-29.0)	42.3% (-7.3%-126.6%)
**Ethiopia**	807.2 (699.6-926.8)	-1.3% (-3.4%-0.9%)	85.5 (73.6-98.0)	-1.3% (-3.4%-0.8%)	1.3 (0.7-1.9)	49.4% (-6.2%-125.7%)	4.6 (2.2-8.3)	-10.9% (-15.2%--5.9%)	20.4 (11.2-29.3)	35.8% (-19.3%-111.3%)
**Kenya**	995.1 (860.7-1140.3)	1.8% (0.9%-2.6%)	101.2 (87.7-115.7)	1.0% (0.1%-1.9%)	1.3 (0.6-1.8)	59.9% (23.2%-103.9%)	5.6 (2.6-10.1)	-2.2% (-4.0%-0.3%)	20.0 (10.4-27.1)	57.1% (22.4%-103.6%)
**Madagascar**	915.9 (792.6-1053.2)	2.4% (-1.8%-6.8%)	96.6 (83.4-110.9)	3.2% (-1.1%-7.6%)	1.6 (0.7-2.4)	23.0% (-16.3%-74.4%)	5.4 (2.6-9.5)	-2.4% (-9.5%-5.3%)	26.5 (13.1-39.3)	23.7% (-18.7%-82.5%)
**Malawi**	1030.7 (891.5-1183.5)	-2.9% (-7.0%-1.3%)	108.3 (93.5-123.5)	-3.0% (-7.3%-0.9%)	1.2 (0.5-1.7)	34.8% (-3.4%-89.8%)	6.0 (2.8-10.7)	-8.1% (-15.8%--1.0%)	18.1 (9.0-26.2)	37.1% (-3.6%-95.3%)
**Mozambique**	1058.9 (916.1-1215.9)	6.5% (1.8%-11.5%)	110.7 (95.7-126.2)	4.1% (0.1%-8.6%)	1.7 (0.8-2.4)	69.8% (22.0%-151.7%)	6.2 (2.9-11.1)	-2.0% (-9.0%-7.0%)	27.3 (14.1-39.0)	74.3% (24.0%-162.2%)
**Rwanda**	1017.1 (875.3-1171.1)	0.6% (-3.6%-5.4%)	105.7 (90.9-121.6)	-0.3% (-4.4%-4.9%)	1.2 (0.5-1.9)	15.0% (-28.2%-70.3%)	5.7 (2.7-10.5)	-10.9% (-18.3%--2.6%)	17.8 (7.5-28.3)	6.4% (-35.7%-59.8%)
**Somalia**	974.1 (839.7-1114.9)	5.4% (0.7%-10.8%)	106.1 (91.8-121.3)	5.9% (1.6%-11.1%)	0.8 (0.3-1.4)	-2.2% (-34.8%-40.4%)	6.1 (2.8-10.8)	2.6% (-5.7%-11.2%)	12.5 (4.9-22.2)	-5.9% (-37.8%-35.5%)

**South Sudan**	963.5 (835.2-1097.5)	8.3% (3.7%-12.8%)	94.5 (81.9-108.7)	10.1% (5.4%-14.8%)	1.4 (0.6-2.1)	11.1% (-23.1%-60.4%)	5.8 (2.7-10.3)	5.2% (-2.5%-13.8%)	21.9 (10.0-33.9)	4.3% (-30.7%-58.4%)
**Uganda**	933.8 (804.9-1072.6)	3.8% (-0.7%-8.4%)	97.5 (83.7-111.9)	2.2% (-2.6%-7.2%)	1.3 (0.5-2.0)	44.7% (2.4%-116.7%)	5.3 (2.5-9.5)	-3.0% (-10.5%-5.9%)	21.2 (8.0-32.5)	40.9% (-0.7%-114.6%)
**United Republic of Tanzania**	1015.9 (874.6-1162.2)	9.6% (5.0%-14.5%)	104.1 (90.8-119.6)	7.8% (3.2%-12.6%)	1.7 (0.6-2.7)	39.0% (-9.0%-101.0%)	5.8 (2.7-10.2)	3.6% (-4.5%-12.9%)	26.9 (10.3-42.3)	35.9% (-13.7%-104.5%)
**Zambia**	974.6 (833.0-1115.7)	1.0% (-3.4%-5.6%)	99.8 (85.7-114.3)	0.2% (-4.1%-4.5%)	1.9 (1.1-2.9)	85.1% (22.2%-181.1%)	5.5 (2.6-9.9)	-8.8% (-16.4%-0.0%)	30.0 (18.9-46.6)	90.0% (23.8%-186.4%)
**Southern Sub-Saharan Africa**	1307.5 (1135.5-1490.7)	-13.5% (-14.9%--12.0%)	126.2 (109.5-144.8)	-11.4% (-12.9%--9.8%)	2.0 (1.6-2.2)	49.8% (27.5%-70.6%)	7.0 (3.3-12.5)	-18.4% (-20.8%--15.5%)	34.8 (27.5-39.7)	44.3% (21.2%-67.0%)
**Botswana**	1302.6 (1127.5-1502.0)	1.2% (-3.6%-6.3%)	123.5 (106.5-142.5)	-1.5% (-5.6%-3.2%)	1.3 (0.8-1.8)	53.9% (4.6%-124.3%)	6.6 (3.1-11.7)	-9.5% (-17.0%--1.5%)	24.7 (15.7-35.5)	46.9% (-3.4%-124.9%)
**Eswatini**	1381.0 (1183.2-1590.2)	3.0% (-1.7%-7.9%)	133.6 (115.6-154.3)	2.4% (-2.0%-7.4%)	1.1 (0.7-1.6)	62.7% (11.3%-134.7%)	7.7 (3.6-13.7)	-3.2% (-10.6%-4.9%)	20.2 (13.5-29.4)	54.9% (3.2%-128.1%)
**Lesotho**	1326.7	9.3%	134.2 (116.0-153.6)	7.8%	0.9 (0.6-1.3)	91.4%	7.6 (3.5-13.9)	5.5%	17.3 (11.2-24.4)	78.3%

	(1145.7-1519.8)	(3.9%-14.8%)		(2.9%-12.6%)		(33.8%-178.5%)		(-2.8%-14.5%)		(20.0%-169.5%)
**Namibia**	1225.3 (1061.5-1400.9)	-5.2% (-9.7%--0.5%)	118.9 (103.0-137.2)	-5.3% (-9.5%--0.5%)	1.3 (0.8-1.8)	43.8% (1.6%-103.5%)	6.5 (3.0-11.6)	-16.2% (-22.9%--8.8%)	23.8 (15.4-33.7)	32.6% (-12.3%-94.8%)
**South Africa**	1311.6 (1139.3-1497.0)	-18.0% (-19.7%--16.3%)	125.4 (108.8-143.8)	-16.0% (-17.9%--14.3%)	2.3 (1.8-2.6)	45.2% (22.7%-66.0%)	6.9 (3.3-12.5)	-23.0% (-25.7%--19.9%)	40.9 (32.5-46.7)	38.6% (15.5%-61.1%)
**Zimbabwe**	1279.2 (1108.2-1477.1)	7.9% (2.2%-13.1%)	131.7 (114.5-152.4)	10.6% (5.2%-15.7%)	0.1 (0.1-0.1)	14.9% (-13.5%-54.1%)	7.3 (3.5-13.0)	9.5% (1.1%-17.9%)	1.6 (1.0-2.1)	13.2% (-15.0%-51.9%)
**Western Sub-Saharan Africa**	902.7 (783.4-1036.4)	4.3% (3.5%-5.1%)	93.5 (81.0-107.2)	4.3% (3.5%-5.0%)	0.3 (0.2-0.4)	38.3% (-8.9%-94.0%)	5.1 (2.4-9.2)	-2.5% (-4.6%-0.0%)	5.4 (2.9-7.3)	33.3% (-13.6%-89.7%)
**Benin**	963.4 (839.0-1108.9)	2.4% (-2.4%-7.3%)	100.4 (86.8-116.0)	2.1% (-2.4%-7.2%)	0.3 (0.2-0.5)	51.0% (1.4%-125.0%)	5.6 (2.6-10.0)	-3.0% (-10.6%-4.9%)	5.4 (2.8-8.0)	36.8% (-12.3%-112.4%)
**Burkina Faso**	878.8 (762.0-1011.5)	3.2% (-1.1%-8.0%)	93.1 (80.4-106.1)	2.4% (-1.6%-7.0%)	0.4 (0.2-0.7)	105.6% (29.9%-209.5%)	5.1 (2.4-9.1)	-2.2% (-9.3%-6.2%)	7.7 (3.6-13.0)	97.6% (24.1%-215.0%)
**Cabo Verde**	1003.1 (864.9-1152.1)	4.3% (0.0%-9.0%)	98.3 (84.4-113.3)	1.4% (-3.1%-6.5%)	0.6 (0.3-0.8)	96.1% (32.1%-226.8%)	5.0 (2.3-9.2)	-7.8% (-15.4%-0.2%)	8.8 (4.7-12.3)	77.8% (13.2%-205.3%)
**Cameroon**	908.4	5.8%	94.3 (81.9-108.5)	6.8%	0.5 (0.3-0.7)	67.6%	5.1 (2.4-9.1)	-1.6% (-9.9%-7.0%)	7.5 (4.0-11.9)	55.2%

	(784.1-1043.2)	(0.9%-10.6%)		(2.4%-11.7%)		(10.6%-150.8%)				(-5.6%-154.9%)
**Chad**	853.8 (740.3-978.1)	3.4% (-0.8%-7.7%)	90.1 (77.9-103.0)	2.7% (-1.8%-7.4%)	0.3 (0.2-0.5)	36.9% (-5.3%-98.9%)	5.2 (2.4-9.1)	0.3% (-7.3%-7.6%)	5.1 (2.5-8.0)	29.4% (-15.0%-95.0%)
**Côte d'Ivoire**	972.3 (836.3-1116.3)	2.6% (-1.5%-7.4%)	100.1 (86.9-114.7)	4.0% (-0.2%-8.8%)	0.3 (0.2-0.5)	32.7% (-10.7%-102.1%)	5.6 (2.7-9.9)	-0.7% (-7.8%-8.0%)	5.2 (3.0-8.1)	26.1% (-18.3%-106.7%)
**Gambia**	1002.5 (867.2-1152.0)	1.7% (-2.8%-6.7%)	104.7 (91.0-119.9)	3.1% (-1.5%-8.0%)	0.4 (0.2-0.6)	73.7% (11.8%-164.3%)	5.7 (2.7-10.3)	-3.6% (-10.8%-4.0%)	6.2 (3.1-9.5)	66.8% (2.5%-168.2%)
**Ghana**	950.8 (822.6-1098.0)	4.0% (-0.6%-8.6%)	97.6 (84.9-112.3)	2.4% (-1.9%-6.6%)	0.1 (0.1-0.2)	4.1% (-30.0%-56.7%)	5.2 (2.4-9.3)	-3.9% (-11.8%-4.8%)	1.9 (1.4-3.0)	-11.6% (-46.8%-49.1%)
**Guinea**	849.8 (734.7-973.8)	3.9% (-0.6%-8.5%)	90.2 (77.8-103.0)	4.2% (-0.3%-8.7%)	0.3 (0.2-0.5)	42.0% (-4.9%-121.4%)	5.1 (2.4-9.0)	-1.2% (-8.4%-6.5%)	5.4 (2.6-9.1)	33.4% (-15.1%-126.7%)
**Guinea-Bissau**	931.4 (804.6-1067.6)	1.1% (-3.3%-6.2%)	98.8 (85.5-112.7)	1.6% (-3.0%-5.7%)	0.4 (0.2-0.6)	52.1% (-8.7%-141.1%)	5.6 (2.7-10.0)	-4.6% (-11.9%-3.3%)	5.9 (3.1-9.8)	42.1% (-16.5%-137.5%)
**Liberia**	972.9 (839.0-1115.6)	1.7% (-2.8%-6.6%)	103.6 (89.4-119.5)	3.3% (-0.8%-8.0%)	0.3 (0.2-0.5)	36.6% (-6.1%-102.1%)	5.5 (2.6-9.9)	-6.2% (-13.2%-2.2%)	5.2 (2.7-8.6)	27.8% (-16.3%-98.8%)
**Mali**	879.2 (762.3-1003.9)	4.6% (-0.1%-9.4%)	93.1 (80.3-106.9)	4.2% (-0.2%-8.6%)	0.3 (0.2-0.5)	50.1% (2.0%-124.1%)	5.1 (2.4-9.2)	-2.4% (-9.4%-5.4%)	5.2 (2.7-8.6)	41.9% (-11.3%-133.0%)

**Mauritania**	920.1 (796.3-1051.7)	3.3% (-0.7%-7.5%)	95.0 (82.3-109.2)	3.7% (-0.3%-8.0%)	0.4 (0.2-0.6)	25.4% (-18.8%-91.8%)	5.1 (2.4-9.1)	-6.8% (-13.9%-1.1%)	6.3 (2.8-10.2)	14.6% (-30.9%-85.1%)
**Niger**	861.0 (746.2-974.6)	4.6% (0.3%-9.1%)	92.3 (79.4-105.4)	5.8% (1.3%-10.0%)	0.3 (0.1-0.5)	32.6% (-12.5%-97.1%)	5.1 (2.4-9.2)	-2.7% (-10.1%-5.0%)	4.6 (2.2-8.3)	27.1% (-19.9%-104.8%)
**Nigeria**	882.1 (764.1-1009.6)	5.0% (4.0%-6.0%)	90.3 (78.1-103.7)	4.7% (3.7%-5.7%)	0.4 (0.2-0.5)	32.9% (-27.9%-117.3%)	4.9 (2.3-8.8)	-2.4% (-4.5%--0.1%)	5.6 (2.8-8.3)	30.8% (-32.1%-120.0%)
**Sao Tome and Principe**	960.1 (828.5-1104.4)	3.2% (-0.9%-7.7%)	98.5 (84.9-113.4)	3.4% (-0.9%-8.0%)	0.4 (0.2-0.6)	84.8% (25.6%-174.9%)	5.2 (2.4-9.1)	-6.8% (-14.1%-0.6%)	7.0 (3.7-10.4)	77.6% (17.3%-180.1%)
**Senegal**	950.3 (822.0-1089.4)	-0.9% (-4.9%-3.6%)	98.7 (85.1-112.8)	-0.2% (-4.2%-4.2%)	0.4 (0.2-0.5)	41.6% (-2.9%-109.1%)	5.4 (2.5-9.8)	-6.9% (-13.8%-0.5%)	6.1 (3.0-9.3)	35.0% (-11.7%-115.2%)
**Sierra Leone**	999.0 (866.8-1153.8)	-4.1% (-8.3%-0.4%)	105.3 (91.5-120.8)	-2.8% (-6.9%-1.6%)	0.3 (0.1-0.4)	39.3% (-9.8%-105.0%)	5.8 (2.7-10.5)	-9.9% (-16.5%--3.0%)	4.5 (2.3-7.5)	34.6% (-15.0%-115.4%)
**Togo**	958.8 (829.1-1103.5)	3.0% (-1.7%-7.6%)	100.9 (87.3-116.7)	3.8% (-0.6%-8.2%)	0.4 (0.2-0.6)	45.4% (-4.0%-113.9%)	5.4 (2.6-9.8)	-2.0% (-9.7%-6.1%)	6.5 (3.1-10.4)	37.6% (-13.3%-117.8%)

Data in parentheses are 95% uncertainty intervals.
SDI, socio-demographic index; YLDs, years lived with disability; YLLs, years of life lost.
