# Supplementary material for: Burden of Peripheral Artery Disease and Its Attributable Risk Factors in 204 Countries and Territories From 1990 to 2019
Source: Front Cardiovasc Med. 2022 Apr 12;9:868370. doi: 10.3389/fcvm.2022.868370 (PMC9039520; doi:10.3389/fcvm.2022.868370)
Supplement: Supplementary file 4 [file Table_4.pdf]

Table S4. Percentage contribution of major risk factors to peripheral artery disease age-standardized deaths by SDI quintile and GBD region, 1990-2019

	1990 PAF		2019 PAF		Percentage change in PAF, 1990-2019	
	**Females**	**Males**	**Females**	**Males**	**Females**	**Males**
**Diet high in sodium**						
**Global**	2.0% (0.4%-6.1%)	3.4% (0.8%-8.4%)	1.9% (0.3%-6.1%)	3.3% (0.7%-8.5%)	-4.3% (-26.7%-5.2%)	-1.7% (-23.6%-7.6%)
**High SDI**	1.6% (0.3%-5.5%)	2.5% (0.4%-7.2%)	1.6% (0.3%-5.5%)	2.7% (0.4%-7.8%)	-3.9% (-23.6%-10.2%)	10.5% (-15.6%-33.8%)
**High-middle SDI**	2.3% (0.5%-6.5%)	4.4% (1.3%-9.7%)	2.1% (0.4%-6.4%)	4.0% (1.0%-9.2%)	-7.5% (-31.2%-7.7%)	-8.2% (-29.5%-2.3%)
**Middle SDI**	3.0% (0.5%-8.0%)	4.1% (1.0%-9.6%)	2.5% (0.4%-7.4%)	4.0% (1.0%-9.4%)	-16.5% (-45.6%--4.3%)	-2.3% (-20.6%-15.2%)
**Low-middle SDI**	2.8% (0.4%-7.9%)	3.3% (0.6%-8.6%)	2.5% (0.3%-7.5%)	3.2% (0.5%-8.4%)	-11.3% (-38.2%--1.5%)	-4.1% (-26.5%-8.4%)
**Low SDI**	3.5% (0.3%-10.0%)	3.7% (0.5%-10.2%)	3.0% (0.3%-9.2%)	3.3% (0.3%-9.6%)	-13.1% (-43.2%--0.9%)	-11.9% (-53.5%-8.1%)
**Central Asia**	3.9% (0.6%-9.8%)	5.9% (1.5%-12.3%)	2.6% (0.3%-7.9%)	4.1% (0.6%-10.0%)	-33.3% (-71.7%--16.8%)	-30.9% (-66.3%--15.7%)
**Central Europe**	7.8% (2.5%-15.0%)	12.6% (6.1%-20.2%)	5.6% (1.1%-12.4%)	10.4% (4.2%-17.8%)	-27.8% (-65.2%--12.3%)	-17.6% (-40.0%--7.0%)
**Eastern Europe**	1.6% (0.2%-5.6%)	3.2% (0.4%-8.8%)	1.6% (0.2%-5.6%)	3.4% (0.4%-8.9%)	-1.6% (-26.0%-27.5%)	5.6% (-29.5%-64.2%)
**Australasia**	0.8% (0.2%-3.7%)	1.3% (0.2%-4.9%)	0.8% (0.2%-3.4%)	1.2% (0.2%-4.6%)	-1.6% (-34.6%-44.9%)	-4.7% (-32.0%-28.9%)
**High-income Asia Pacific**	5.4% (1.1%-12.0%)	7.6% (2.3%-14.5%)	3.6% (0.5%-9.4%)	5.1% (1.0%-11.6%)	-32.8% (-63.6%--17.6%)	-32.5% (-63.3%--17.1%)
**High-income North America**	1.6% (0.2%-5.5%)	1.8% (0.2%-6.2%)	1.6% (0.2%-5.7%)	2.5% (0.3%-7.4%)	-1.2% (-22.3%-28.2%)	42.8% (9.9%-141.0%)

**Southern Latin America**	2.6% (0.3%-7.8%)	3.4% (0.3%-9.3%)	2.5% (0.2%-7.6%)	3.6% (0.3%-9.5%)	-5.9% (-45.9%-14.6%)	4.5% (-36.1%-48.2%)
**Western Europe**	1.4% (0.2%-5.2%)	2.4% (0.3%-7.2%)	1.3% (0.2%-4.7%)	2.3% (0.3%-7.2%)	-8.9% (-23.6%-5.0%)	-2.5% (-20.7%-21.6%)
**Andean Latin America**	2.4% (0.2%-7.2%)	3.4% (0.3%-9.1%)	2.3% (0.2%-7.3%)	3.3% (0.3%-8.9%)	-3.8% (-35.4%-28.4%)	-1.8% (-32.1%-51.1%)
**Caribbean**	1.8% (0.2%-6.2%)	2.6% (0.2%-7.8%)	1.5% (0.2%-5.7%)	2.4% (0.2%-7.6%)	-13.9% (-41.6%-3.8%)	-6.2% (-37.0%-20.3%)
**Central Latin America**	2.1% (0.2%-7.0%)	3.9% (0.6%-9.6%)	2.2% (0.2%-6.9%)	4.4% (0.9%-10.4%)	2.5% (-17.2%-31.9%)	14.0% (-6.1%-70.6%)
**Tropical Latin America**	2.7% (0.2%-7.9%)	3.9% (0.3%-9.9%)	2.4% (0.2%-7.4%)	3.7% (0.4%-9.6%)	-9.9% (-42.2%-25.2%)	-5.0% (-40.1%-63.5%)
**North Africa and Middle East**	0.5% (0.3%-1.5%)	0.9% (0.2%-3.4%)	0.5% (0.3%-1.6%)	0.9% (0.3%-3.4%)	-0.3% (-28.2%-39.0%)	4.0% (-23.3%-46.4%)
**South Asia**	2.0% (0.2%-6.6%)	2.3% (0.3%-7.3%)	1.8% (0.2%-6.2%)	2.6% (0.3%-7.7%)	-6.0% (-17.6%-13.0%)	12.0% (1.7%-49.8%)
**East Asia**	5.9% (1.7%-12.3%)	8.2% (3.2%-15.3%)	5.1% (1.2%-11.2%)	7.5% (2.8%-14.2%)	-13.9% (-43.9%-5.9%)	-8.7% (-33.1%-15.2%)
**Oceania**	3.5% (0.4%-9.0%)	4.7% (0.8%-10.8%)	3.3% (0.3%-8.8%)	4.5% (0.6%-10.5%)	-5.6% (-49.7%-13.2%)	-5.4% (-44.0%-21.0%)
**Southeast Asia**	4.7% (0.7%-11.1%)	7.9% (2.5%-14.9%)	3.8% (0.4%-9.7%)	6.2% (1.3%-12.7%)	-20.3% (-57.5%--8.4%)	-22.4% (-54.9%--9.1%)
**Central Sub-Saharan Africa**	1.3% (0.2%-5.4%)	1.4% (0.2%-5.6%)	1.3% (0.2%-5.9%)	1.5% (0.2%-6.3%)	3.7% (-43.6%-42.2%)	10.5% (-41.7%-68.8%)
**Eastern Sub-Saharan Africa**	5.3% (0.5%-13.7%)	5.6% (0.7%-13.9%)	4.7% (0.3%-12.7%)	4.2% (0.3%-11.7%)	-11.0% (-45.4%--0.8%)	-25.4% (-65.5%--8.7%)
**Southern Sub-Saharan Africa**	1.7% (0.2%-6.9%)	1.8% (0.2%-6.9%)	1.5% (0.2%-6.2%)	1.4% (0.2%-5.9%)	-12.9% (-41.7%-26.5%)	-24.0% (-53.2%-41.4%)

**Western Sub-Saharan Africa**	2.0% (0.2%-7.7%)	2.1% (0.2%-8.2%)	2.2% (0.2%-8.3%)	2.3% (0.2%-8.3%)	8.0% (-16.8%-41.4%)	6.4% (-16.4%-42.2%)
**High fasting plasma glucose**						
**Global**	19.4% (16.1%-22.9%)	21.1% (18.0%-24.4%)	25.2% (21.2%-29.3%)	28.4% (24.6%-32.3%)	30.0% (25.3%-36.9%)	34.5% (30.6%-40.0%)
**High SDI**	21.4% (17.9%-25.0%)	23.3% (19.8%-26.7%)	28.4% (24.2%-32.7%)	32.7% (28.7%-36.8%)	32.8% (26.7%-40.0%)	40.6% (35.9%-46.8%)
**High-middle SDI**	16.8% (13.9%-20.0%)	18.7% (16.0%-21.5%)	21.7% (18.1%-25.5%)	24.4% (21.1%-28.0%)	29.0% (22.7%-35.6%)	30.6% (25.8%-37.1%)
**Middle SDI**	23.8% (19.7%-27.6%)	24.7% (20.6%-29.0%)	27.1% (23.0%-31.5%)	28.8% (24.8%-33.0%)	13.9% (8.0%-21.2%)	16.8% (10.6%-24.1%)
**Low-middle SDI**	19.2% (16.0%-22.7%)	21.1% (17.9%-24.5%)	24.8% (20.9%-28.6%)	27.4% (23.9%-31.4%)	28.9% (23.3%-35.4%)	30.3% (24.9%-36.5%)
**Low SDI**	16.6% (13.8%-19.5%)	20.7% (17.5%-23.8%)	21.2% (17.7%-25.4%)	24.8% (21.6%-28.4%)	27.9% (22.2%-36.0%)	19.6% (12.6%-26.6%)
**Central Asia**	14.7% (12.1%-17.3%)	14.1% (12.2%-16.3%)	23.7% (19.8%-27.5%)	23.8% (20.8%-27.0%)	61.2% (51.7%-71.9%)	68.8% (60.0%-78.8%)
**Central Europe**	21.1% (17.7%-24.6%)	23.0% (20.0%-26.2%)	26.5% (22.2%-30.8%)	31.4% (27.6%-35.1%)	25.5% (17.1%-32.8%)	36.8% (30.3%-43.5%)
**Eastern Europe**	12.8% (10.6%-15.1%)	13.7% (11.8%-15.9%)	16.3% (13.5%-19.2%)	18.0% (15.6%-20.6%)	27.7% (23.3%-32.3%)	30.9% (25.6%-37.5%)
**Australasia**	15.9% (12.6%-19.3%)	19.3% (15.8%-23.0%)	21.3% (17.3%-25.6%)	25.2% (21.1%-29.6%)	34.0% (20.6%-49.7%)	30.9% (19.9%-45.2%)
**High-income Asia Pacific**	15.1% (12.4%-18.1%)	21.5% (18.6%-24.7%)	17.3% (14.0%-20.9%)	23.9% (20.3%-27.7%)	14.6% (8.7%-21.6%)	11.0% (6.2%-16.9%)
**High-income North America**	22.1% (18.8%-25.4%)	25.4% (22.0%-29.0%)	29.5% (25.4%-33.4%)	36.0% (31.7%-40.1%)	33.7% (29.4%-38.3%)	41.3% (35.5%-47.2%)
**Southern Latin America**	19.3% (15.9%-22.7%)	19.2% (16.4%-22.0%)	27.3% (22.8%-31.6%)	29.8% (25.9%-33.9%)	41.5% (32.2%-52.8%)	55.4% (45.6%-68.6%)

**Western Europe**	21.9% (18.0%-25.9%)	22.6% (19.1%-26.1%)	28.1% (23.4%-32.8%)	30.9% (26.8%-35.1%)	28.6% (20.4%-38.6%)	36.7% (31.4%-44.0%)
**Andean Latin America**	16.6% (13.4%-20.0%)	17.8% (15.0%-20.9%)	23.9% (19.9%-28.0%)	25.4% (21.4%-29.4%)	43.8% (33.4%-58.1%)	42.1% (33.6%-51.4%)
**Caribbean**	26.9% (22.9%-30.9%)	29.9% (26.0%-34.0%)	31.7% (27.2%-36.1%)	36.8% (32.4%-41.2%)	17.6% (11.4%-23.7%)	22.8% (17.1%-29.6%)
**Central Latin America**	34.5% (29.7%-39.2%)	33.7% (29.1%-38.2%)	33.5% (28.8%-38.5%)	35.6% (31.2%-40.1%)	-2.9% (-6.8%-0.9%)	5.7% (2.0%-9.5%)
**Tropical Latin America**	23.5% (20.1%-27.1%)	25.9% (22.6%-29.6%)	25.9% (22.1%-29.8%)	28.4% (24.6%-32.4%)	10.1% (6.1%-13.5%)	9.5% (5.9%-14.0%)
**North Africa and Middle East**	20.4% (16.9%-23.9%)	20.5% (17.7%-23.6%)	28.4% (23.6%-32.7%)	30.0% (26.3%-34.0%)	39.2% (29.2%-49.8%)	46.2% (36.1%-57.0%)
**South Asia**	19.1% (15.7%-22.5%)	20.8% (17.8%-24.2%)	26.4% (22.3%-30.8%)	28.3% (24.7%-32.3%)	38.3% (32.5%-45.1%)	35.9% (30.8%-41.8%)
**East Asia**	18.8% (15.8%-22.0%)	18.7% (15.9%-21.8%)	19.2% (16.3%-22.6%)	21.3% (18.3%-24.9%)	2.1% (-3.7%-7.6%)	13.9% (7.9%-19.7%)
**Oceania**	22.5% (18.4%-26.9%)	27.8% (23.9%-31.8%)	33.4% (28.4%-38.3%)	34.4% (30.4%-38.7%)	48.5% (37.9%-61.4%)	23.7% (13.6%-35.6%)
**Southeast Asia**	18.1% (14.9%-21.5%)	19.7% (16.8%-22.8%)	25.9% (21.8%-30.1%)	27.4% (23.9%-31.1%)	43.1% (35.6%-52.5%)	39.3% (33.4%-46.0%)
**Central Sub-Saharan Africa**	17.9% (14.9%-21.0%)	24.5% (21.0%-28.0%)	22.0% (18.6%-25.6%)	30.2% (26.5%-34.1%)	23.2% (16.4%-31.3%)	23.3% (16.2%-31.9%)
**Eastern Sub-Saharan Africa**	14.4% (11.8%-17.1%)	17.7% (15.0%-20.5%)	16.3% (13.4%-19.2%)	21.4% (18.4%-24.7%)	13.2% (9.1%-17.7%)	20.8% (15.5%-26.5%)
**Southern Sub-Saharan Africa**	23.6% (20.0%-27.1%)	21.7% (19.0%-24.7%)	29.5% (25.6%-33.5%)	29.4% (26.0%-32.8%)	24.9% (19.8%-31.3%)	35.1% (29.3%-42.2%)
**Western Sub-Saharan Africa**	15.8% (13.5%-18.3%)	17.5% (14.8%-20.5%)	21.1% (18.1%-24.1%)	22.6% (19.0%-26.4%)	33.3% (27.1%-39.6%)	28.6% (23.4%-34.0%)
**High systolic blood pressure**						

**Global**	29.0% (20.5%-39.4%)	28.3% (20.8%-36.8%)	25.3% (17.8%-35.2%)	25.0% (18.1%-33.0%)	-12.8% (-18.4%--7.5%)	-11.8% (-16.3%--7.2%)
**High SDI**	29.8% (21.2%-40.5%)	29.2% (21.5%-38.2%)	24.7% (17.1%-34.2%)	23.7% (17.0%-31.5%)	-17.4% (-24.8%--9.5%)	-19.0% (-25.1%--13.4%)
**High-middle SDI**	29.7% (21.5%-40.2%)	29.9% (22.7%-37.7%)	26.3% (18.8%-35.8%)	27.1% (20.2%-34.9%)	-11.3% (-17.2%--5.9%)	-9.2% (-13.4%--5.4%)
**Middle SDI**	23.8% (16.8%-32.4%)	23.4% (17.1%-30.8%)	25.4% (17.9%-34.1%)	25.4% (19.0%-33.1%)	6.9% (-2.3%-16.9%)	8.5% (2.2%-15.5%)
**Low-middle SDI**	24.8% (17.6%-33.4%)	22.1% (16.2%-28.9%)	26.2% (19.0%-35.1%)	24.4% (17.9%-31.9%)	5.8% (-0.5%-12.6%)	10.0% (4.6%-15.3%)
**Low SDI**	24.2% (18.2%-32.0%)	23.0% (17.2%-29.5%)	25.2% (18.5%-33.2%)	25.9% (19.6%-32.8%)	4.1% (-5.1%-13.6%)	12.7% (0.3%-25.6%)
**Central Asia**	27.0% (19.6%-36.4%)	27.2% (21.0%-34.2%)	27.7% (19.6%-37.9%)	27.0% (20.6%-34.3%)	2.3% (-9.4%-15.7%)	-1.0% (-10.1%-8.6%)
**Central Europe**	32.7% (23.7%-42.7%)	34.3% (26.8%-42.4%)	26.0% (18.4%-35.2%)	30.1% (23.1%-37.8%)	-20.6% (-29.7%--11.5%)	-12.2% (-19.7%--6.1%)
**Eastern Europe**	29.9% (22.0%-39.8%)	29.1% (22.4%-36.3%)	28.9% (21.0%-38.6%)	29.7% (22.9%-37.1%)	-3.2% (-10.7%-5.0%)	2.1% (-3.4%-8.3%)
**Australasia**	28.5% (19.2%-39.7%)	26.9% (19.4%-36.1%)	22.8% (14.8%-32.7%)	22.4% (15.1%-30.9%)	-20.2% (-36.1%--1.0%)	-16.9% (-28.2%--4.9%)
**High-income Asia Pacific**	28.9% (20.3%-39.7%)	30.2% (22.3%-38.8%)	21.3% (13.7%-30.7%)	24.1% (17.3%-32.1%)	-26.5% (-37.5%--15.7%)	-20.2% (-28.9%--12.3%)
**High-income North America**	29.2% (21.5%-38.7%)	28.0% (20.9%-36.3%)	23.0% (15.8%-31.7%)	20.4% (14.3%-27.8%)	-21.1% (-32.9%--8.9%)	-26.9% (-36.3%--18.0%)
**Southern Latin America**	22.6% (15.1%-31.7%)	22.2% (15.9%-29.3%)	27.1% (18.2%-37.7%)	28.2% (20.6%-37.1%)	20.1% (-2.8%-49.5%)	27.2% (9.4%-49.4%)
**Western Europe**	30.7% (21.3%-42.0%)	31.0% (22.8%-40.4%)	25.8% (17.9%-36.5%)	25.9% (18.9%-34.6%)	-15.7% (-23.7%--7.4%)	-16.4% (-22.4%--10.5%)

**Andean Latin America**	13.2% (7.8%-20.3%)	14.1% (9.3%-19.7%)	22.2% (14.6%-31.6%)	20.0% (13.9%-27.1%)	68.6% (26.8%-137.6%)	41.8% (17.8%-76.2%)
**Caribbean**	22.8% (16.0%-31.2%)	22.3% (16.2%-29.3%)	22.3% (15.6%-30.3%)	23.5% (17.1%-31.0%)	-2.1% (-18.4%-16.6%)	5.6% (-7.7%-19.9%)
**Central Latin America**	21.6% (14.6%-29.9%)	22.1% (15.5%-30.0%)	24.4% (17.1%-33.5%)	24.4% (17.6%-32.5%)	13.3% (-5.9%-37.3%)	10.0% (-3.2%-25.1%)
**Tropical Latin America**	25.3% (18.4%-33.6%)	25.5% (19.5%-32.2%)	25.8% (18.8%-34.3%)	26.7% (20.3%-34.1%)	1.9% (-6.7%-11.6%)	4.5% (-1.7%-11.3%)
**North Africa and Middle East**	26.8% (18.8%-36.2%)	24.2% (17.9%-31.3%)	25.7% (17.8%-35.0%)	24.3% (18.2%-31.4%)	-4.3% (-17.0%-11.1%)	0.6% (-10.6%-13.7%)
**South Asia**	25.4% (18.0%-34.4%)	21.2% (15.5%-27.9%)	25.2% (17.8%-34.1%)	23.6% (17.2%-30.9%)	-0.6% (-6.9%-6.0%)	11.0% (4.5%-17.7%)
**East Asia**	21.6% (14.3%-30.7%)	19.6% (13.2%-27.1%)	24.7% (16.2%-35.0%)	24.1% (16.9%-33.0%)	14.6% (-15.5%-53.0%)	23.4% (-0.9%-54.3%)
**Oceania**	17.2% (11.6%-24.6%)	15.8% (11.4%-21.0%)	20.0% (13.6%-28.7%)	17.6% (12.1%-23.8%)	16.5% (-6.2%-43.0%)	11.9% (-6.4%-34.1%)
**Southeast Asia**	28.7% (20.6%-38.6%)	27.7% (21.2%-34.9%)	29.2% (20.6%-39.1%)	29.3% (22.4%-36.6%)	1.7% (-7.6%-10.5%)	5.9% (-1.0%-12.6%)
**Central Sub-Saharan Africa**	29.9% (21.2%-39.9%)	27.6% (20.4%-35.4%)	28.0% (20.3%-36.4%)	27.0% (20.4%-34.7%)	-6.1% (-20.7%-12.2%)	-2.0% (-16.3%-14.9%)
**Eastern Sub-Saharan Africa**	22.0% (15.8%-29.6%)	19.5% (14.2%-25.3%)	25.0% (17.8%-33.4%)	26.1% (19.4%-33.1%)	13.9% (1.8%-26.6%)	33.7% (22.3%-48.3%)
**Southern Sub-Saharan Africa**	30.4% (21.9%-40.5%)	30.6% (23.6%-38.3%)	31.4% (23.0%-40.5%)	30.5% (23.7%-38.0%)	3.2% (-14.5%-24.6%)	-0.2% (-12.4%-13.5%)
**Western Sub-Saharan Africa**	26.0% (19.5%-33.1%)	22.9% (16.8%-29.6%)	31.7% (24.5%-39.8%)	28.8% (21.2%-37.5%)	21.8% (6.8%-39.5%)	25.9% (14.1%-40.1%)
**Kidney dysfunction**						
**Global**	10.4% (5.4%-15.1%)	11.1% (7.3%-14.7%)	11.7% (6.1%-16.8%)	12.6% (8.2%-16.7%)	12.6% (5.8%-20.1%)	13.7% (6.0%-19.0%)

**High SDI**	11.9% (6.3%-17.1%)	11.9% (7.5%-16.1%)	13.4% (7.4%-18.8%)	12.9% (8.0%-17.4%)	11.8% (4.8%-21.9%)	8.3% (1.7%-14.4%)
**High-middle SDI**	8.9% (5.0%-12.8%)	11.0% (7.9%-14.0%)	10.3% (5.6%-14.8%)	13.0% (9.0%-16.7%)	15.9% (4.9%-24.6%)	17.7% (7.9%-25.9%)
**Middle SDI**	10.7% (6.9%-14.4%)	11.0% (7.7%-14.2%)	13.2% (8.1%-17.8%)	14.1% (10.0%-18.0%)	23.0% (10.4%-36.0%)	28.3% (18.9%-38.4%)
**Low-middle SDI**	9.0% (5.9%-12.4%)	9.8% (7.1%-12.7%)	10.9% (6.7%-14.8%)	11.9% (8.5%-15.2%)	20.5% (7.4%-32.7%)	20.6% (9.8%-29.3%)
**Low SDI**	7.7% (5.1%-10.4%)	8.4% (6.1%-10.9%)	9.1% (6.1%-12.4%)	10.0% (7.3%-12.7%)	18.2% (3.4%-31.1%)	18.3% (4.9%-32.6%)
**Central Asia**	7.8% (4.9%-11.0%)	11.2% (8.5%-14.0%)	9.1% (5.3%-12.7%)	13.7% (10.6%-16.7%)	16.5% (-3.2%-36.3%)	22.2% (6.8%-38.5%)
**Central Europe**	8.1% (5.0%-11.2%)	10.8% (8.2%-13.4%)	10.8% (6.2%-15.0%)	13.8% (10.3%-17.3%)	32.7% (13.5%-48.1%)	27.4% (16.1%-37.9%)
**Eastern Europe**	8.5% (5.0%-12.0%)	11.3% (8.3%-14.3%)	9.5% (5.6%-13.3%)	14.5% (11.1%-17.7%)	12.2% (3.7%-21.0%)	27.9% (15.9%-40.3%)
**Australasia**	11.3% (4.9%-17.2%)	10.7% (5.6%-15.3%)	11.9% (4.4%-18.6%)	11.6% (5.4%-16.9%)	5.0% (-11.3%-14.6%)	8.8% (-6.6%-18.5%)
**High-income Asia Pacific**	10.8% (4.5%-16.4%)	13.7% (8.9%-18.0%)	11.1% (4.3%-17.3%)	14.0% (8.2%-19.1%)	3.1% (-12.8%-16.3%)	2.1% (-12.6%-11.1%)
**High-income North America**	14.5% (9.0%-19.6%)	14.4% (9.8%-18.8%)	15.4% (9.6%-20.8%)	14.5% (9.4%-19.2%)	5.8% (0.1%-10.8%)	1.0% (-8.1%-6.7%)
**Southern Latin America**	10.2% (5.5%-14.6%)	10.9% (7.3%-14.3%)	12.9% (6.6%-18.6%)	13.6% (8.6%-18.4%)	26.3% (10.3%-39.3%)	24.1% (8.3%-35.6%)
**Western Europe**	10.2% (4.5%-15.3%)	10.2% (6.1%-14.0%)	10.8% (4.4%-16.5%)	10.7% (6.0%-15.0%)	6.5% (-5.0%-12.8%)	5.3% (-4.5%-12.0%)
**Andean Latin America**	7.9% (4.2%-11.2%)	8.8% (6.1%-11.7%)	13.7% (7.9%-19.1%)	12.8% (8.4%-17.1%)	73.6% (41.9%-123.6%)	46.0% (13.1%-79.7%)

**Caribbean**	10.1% (6.8%-13.3%)	11.1% (8.1%-14.1%)	13.7% (9.2%-18.1%)	15.2% (11.3%-18.9%)	36.2% (22.9%-50.2%)	36.9% (26.1%-49.7%)
**Central Latin America**	14.7% (9.3%-19.8%)	13.3% (9.2%-17.1%)	18.1% (11.4%-24.0%)	17.8% (12.5%-22.8%)	23.5% (12.1%-36.5%)	33.7% (22.4%-45.6%)
**Tropical Latin America**	11.2% (7.6%-14.6%)	12.8% (9.7%-15.9%)	13.3% (8.9%-17.3%)	14.4% (10.6%-18.1%)	19.5% (9.7%-31.2%)	12.5% (2.2%-23.9%)
**North Africa and Middle East**	12.2% (7.0%-17.0%)	13.4% (9.6%-17.3%)	16.9% (8.2%-24.1%)	19.9% (13.8%-25.3%)	39.1% (4.4%-65.1%)	48.6% (23.0%-72.4%)
**South Asia**	8.5% (4.9%-12.3%)	10.7% (7.6%-14.0%)	9.9% (5.6%-14.0%)	11.5% (7.9%-15.0%)	15.8% (1.0%-32.2%)	7.7% (-4.5%-19.2%)
**East Asia**	11.0% (7.4%-14.5%)	9.6% (6.3%-12.8%)	11.0% (6.7%-14.9%)	10.2% (6.7%-13.6%)	0.4% (-16.1%-13.3%)	7.3% (-8.9%-20.2%)
**Oceania**	8.9% (5.2%-13.0%)	13.2% (9.5%-17.1%)	11.2% (6.5%-15.4%)	15.0% (10.4%-19.6%)	24.9% (3.6%-56.4%)	13.7% (-7.5%-35.7%)
**Southeast Asia**	9.4% (5.7%-12.9%)	12.2% (9.3%-15.2%)	12.2% (7.0%-16.8%)	15.2% (11.7%-18.7%)	29.8% (10.1%-49.5%)	24.2% (13.1%-36.6%)
**Central Sub-Saharan Africa**	6.7% (4.1%-9.3%)	7.5% (5.2%-10.0%)	8.2% (5.3%-11.3%)	8.9% (6.4%-11.6%)	22.3% (-2.9%-54.4%)	19.3% (-1.8%-47.9%)
**Eastern Sub-Saharan Africa**	6.7% (4.3%-9.4%)	8.2% (6.0%-10.6%)	8.0% (5.2%-10.8%)	9.3% (6.9%-12.0%)	19.2% (3.5%-35.2%)	14.5% (3.5%-26.4%)
**Southern Sub-Saharan Africa**	11.0% (7.7%-14.2%)	15.9% (12.6%-19.7%)	13.5% (9.6%-17.4%)	18.4% (14.6%-22.9%)	23.3% (7.8%-40.1%)	15.9% (3.8%-26.6%)
**Western Sub-Saharan Africa**	9.5% (7.0%-12.1%)	6.9% (4.8%-8.9%)	11.1% (7.9%-14.1%)	8.4% (5.6%-11.1%)	16.4% (5.6%-25.6%)	22.4% (7.1%-35.1%)
**Lead exposure**						
**Global**	0.7% (0.2%-1.5%)	1.3% (0.6%-2.2%)	0.8% (0.3%-1.6%)	1.4% (0.6%-2.4%)	12.4% (2.6%-43.1%)	8.7% (-2.2%-25.9%)
**High SDI**	0.7% (0.2%-1.5%)	1.1% (0.4%-2.0%)	0.6% (0.1%-1.3%)	0.9% (0.3%-1.8%)	-20.2% (-56.6%--8.8%)	-17.8% (-38.3%--9.5%)

**High-middle SDI**	0.5% (0.1%-1.2%)	1.0% (0.3%-1.8%)	0.6% (0.2%-1.3%)	1.0% (0.4%-1.9%)	16.7% (3.3%-45.9%)	8.4% (-2.0%-34.1%)
**Middle SDI**	1.4% (0.6%-2.3%)	2.2% (1.2%-3.2%)	1.6% (0.8%-2.6%)	2.4% (1.4%-3.7%)	14.7% (5.2%-32.7%)	12.2% (5.2%-22.1%)
**Low-middle SDI**	2.0% (1.1%-3.2%)	2.9% (1.8%-4.2%)	2.5% (1.4%-3.8%)	3.4% (2.2%-4.9%)	22.4% (9.6%-40.2%)	18.3% (7.3%-30.8%)
**Low SDI**	1.7% (0.9%-2.8%)	2.7% (1.6%-4.0%)	2.2% (1.2%-3.5%)	3.1% (2.0%-4.5%)	25.0% (12.2%-45.7%)	14.1% (2.0%-30.7%)
**Central Asia**	0.7% (0.1%-1.6%)	1.4% (0.6%-2.4%)	0.9% (0.2%-1.7%)	1.4% (0.6%-2.4%)	16.0% (2.3%-96.1%)	3.7% (-7.0%-22.3%)
**Central Europe**	0.5% (0.0%-1.2%)	1.1% (0.3%-1.9%)	0.6% (0.0%-1.3%)	1.2% (0.4%-2.1%)	16.2% (4.0%-275.4%)	8.6% (-1.2%-40.9%)
**Eastern Europe**	0.2% (0.0%-0.7%)	0.4% (0.0%-1.2%)	0.2% (0.0%-0.8%)	0.5% (0.0%-1.2%)	10.1% (-5.3%-495653.1%)	12.1% (0.5%-1046.7%)
**Australasia**	1.2% (0.5%-2.1%)	1.6% (0.8%-2.6%)	1.1% (0.4%-2.1%)	1.5% (0.7%-2.6%)	-4.8% (-16.9%-5.3%)	-2.2% (-11.7%-7.5%)
**High-income Asia Pacific**	0.5% (0.0%-1.3%)	0.9% (0.2%-1.8%)	0.4% (0.0%-1.1%)	0.7% (0.1%-1.5%)	-15.7% (-33.1%-9.2%)	-25.0% (-50.9%--12.1%)
**High-income North America**	0.9% (0.3%-1.8%)	1.5% (0.7%-2.5%)	0.6% (0.1%-1.4%)	1.1% (0.4%-2.0%)	-31.4% (-69.9%--17.4%)	-27.3% (-48.9%--16.0%)
**Southern Latin America**	0.3% (0.0%-1.0%)	0.6% (0.0%-1.3%)	0.3% (0.0%-1.0%)	0.6% (0.1%-1.3%)	6.0% (-5.6%-995.5%)	6.4% (-9.4%-758.8%)
**Western Europe**	0.6% (0.1%-1.4%)	1.1% (0.4%-1.9%)	0.6% (0.1%-1.4%)	1.0% (0.3%-1.9%)	-3.6% (-26.8%-13.7%)	-7.3% (-21.9%-1.1%)
**Andean Latin America**	1.1% (0.4%-2.0%)	1.6% (0.8%-2.6%)	1.3% (0.5%-2.2%)	1.8% (0.9%-2.9%)	16.1% (5.2%-40.7%)	11.6% (-0.5%-28.4%)
**Caribbean**	1.3% (0.6%-2.3%)	2.4% (1.4%-3.6%)	1.4% (0.7%-2.4%)	2.5% (1.5%-3.7%)	7.8% (-1.9%-26.7%)	3.0% (-6.1%-12.5%)
**Central Latin America**	1.8% (0.9%-2.9%)	2.8% (1.7%-4.2%)	1.8% (1.0%-3.0%)	2.9% (1.7%-4.2%)	4.9% (-0.5%-11.7%)	1.1% (-4.8%-7.0%)
**Tropical Latin America**	1.2% (0.4%-2.1%)	1.8% (1.0%-2.8%)	1.2% (0.4%-2.1%)	1.7% (0.8%-2.7%)	-0.4% (-7.0%-10.0%)	-9.2% (-18.9%--2.4%)

**North Africa and Middle East**	1.3% (0.5%-2.2%)	1.7% (0.9%-2.7%)	1.4% (0.6%-2.5%)	2.0% (1.1%-3.1%)	11.5% (-8.2%-42.4%)	19.1% (4.5%-44.6%)
**South Asia**	2.8% (1.7%-4.1%)	3.5% (2.3%-4.9%)	3.4% (2.1%-5.0%)	4.1% (2.7%-5.8%)	21.6% (11.1%-34.2%)	16.5% (7.0%-27.7%)
**East Asia**	1.8% (1.0%-2.8%)	2.6% (1.6%-3.8%)	1.9% (1.0%-3.1%)	2.8% (1.7%-4.2%)	8.4% (-2.6%-22.5%)	8.3% (-1.8%-16.7%)
**Oceania**	0.4% (0.0%-1.1%)	0.7% (0.2%-1.5%)	0.4% (0.0%-1.0%)	0.7% (0.2%-1.4%)	-5.3% (-28.1%-93.3%)	-4.6% (-17.6%-22.6%)
**Southeast Asia**	0.9% (0.3%-1.7%)	1.6% (0.8%-2.5%)	0.9% (0.3%-1.8%)	1.7% (0.8%-2.6%)	5.0% (-3.5%-26.1%)	3.6% (-3.9%-13.0%)
**Central Sub-Saharan Africa**	1.1% (0.4%-2.0%)	1.8% (0.9%-2.7%)	1.4% (0.7%-2.4%)	2.1% (1.2%-3.2%)	28.9% (12.8%-76.8%)	21.4% (8.3%-45.3%)
**Eastern Sub-Saharan Africa**	1.4% (0.6%-2.3%)	3.2% (2.1%-4.5%)	1.5% (0.7%-2.5%)	2.9% (1.8%-4.3%)	4.9% (-1.9%-15.4%)	-7.0% (-15.0%-0.6%)
**Southern Sub-Saharan Africa**	0.6% (0.0%-1.4%)	1.5% (0.7%-2.4%)	0.8% (0.2%-1.6%)	1.5% (0.7%-2.5%)	33.8% (13.1%-296.9%)	-0.7% (-11.9%-12.2%)
**Western Sub-Saharan Africa**	1.2% (0.5%-2.1%)	1.6% (0.8%-2.5%)	1.4% (0.6%-2.3%)	1.9% (1.1%-3.0%)	14.8% (1.4%-49.5%)	21.6% (8.0%-50.0%)
**Tobacco**						
**Global**	17.6% (14.9%-20.3%)	43.6% (38.0%-48.1%)	13.3% (10.8%-15.7%)	33.6% (28.3%-37.8%)	-24.7% (-32.8%--16.8%)	-23.0% (-27.5%--18.5%)
**High SDI**	27.8% (23.6%-32.1%)	44.4% (38.6%-49.2%)	21.0% (17.3%-24.6%)	31.5% (26.1%-36.2%)	-24.6% (-31.4%--18.3%)	-29.1% (-34.7%--22.9%)
**High-middle SDI**	10.1% (8.0%-12.2%)	49.1% (42.7%-53.8%)	10.6% (8.3%-12.8%)	42.4% (36.3%-47.2%)	4.7% (-10.1%-18.4%)	-13.7% (-17.4%--10.2%)
**Middle SDI**	13.9% (10.6%-17.2%)	42.4% (36.4%-47.3%)	9.1% (6.9%-11.3%)	33.8% (27.3%-39.3%)	-34.9% (-44.6%--25.2%)	-20.2% (-27.5%--14.1%)
**Low-middle SDI**	11.1% (8.1%-14.7%)	39.3% (33.3%-44.3%)	8.0% (6.1%-10.0%)	32.7% (26.5%-37.8%)	-28.3% (-40.4%--14.6%)	-16.8% (-23.8%--11.5%)
**Low SDI**	5.5% (3.9%-7.4%)	25.7% (19.5%-31.3%)	5.3% (3.6%-7.9%)	20.1% (15.0%-25.0%)	-4.2% (-20.5%-20.1%)	-21.8% (-28.4%--14.2%)

**Central Asia**	4.6% (3.0%-6.5%)	51.8% (44.2%-57.9%)	3.7% (2.6%-4.9%)	45.7% (38.8%-50.6%)	-19.5% (-37.4%-2.8%)	-11.8% (-17.9%--5.7%)
**Central Europe**	22.2% (17.8%-26.7%)	51.2% (44.0%-56.2%)	18.9% (15.1%-22.4%)	42.2% (35.1%-47.6%)	-15.1% (-28.2%--2.3%)	-17.6% (-22.8%--12.8%)
**Eastern Europe**	5.7% (3.8%-7.7%)	52.6% (44.9%-58.3%)	8.3% (5.8%-10.5%)	54.4% (46.7%-59.7%)	46.1% (14.2%-85.7%)	3.4% (-0.7%-8.3%)
**Australasia**	23.6% (19.7%-27.7%)	34.6% (29.4%-39.4%)	13.4% (10.7%-16.3%)	18.0% (14.4%-21.7%)	-43.0% (-51.8%--32.6%)	-48.0% (-54.4%--40.7%)
**High-income Asia Pacific**	14.8% (11.7%-18.3%)	56.8% (51.2%-61.7%)	9.3% (6.6%-12.1%)	40.5% (34.8%-45.7%)	-37.2% (-50.4%--23.9%)	-28.6% (-33.9%--23.0%)
**High-income North America**	34.5% (28.6%-39.6%)	45.0% (38.2%-50.7%)	23.9% (19.2%-28.2%)	30.4% (24.4%-35.6%)	-30.8% (-37.5%--24.1%)	-32.5% (-39.3%--25.4%)
**Southern Latin America**	20.4% (16.0%-24.6%)	31.4% (25.6%-36.5%)	16.9% (13.4%-20.5%)	23.1% (18.0%-28.1%)	-17.1% (-29.9%--1.4%)	-26.4% (-34.2%--16.8%)
**Western Europe**	22.0% (18.4%-25.3%)	45.8% (40.1%-50.5%)	16.8% (13.8%-20.0%)	32.7% (27.7%-37.2%)	-23.6% (-33.3%--13.9%)	-28.7% (-33.6%--23.3%)
**Andean Latin America**	3.0% (1.9%-4.6%)	20.9% (16.3%-25.8%)	2.3% (1.5%-3.2%)	13.0% (9.7%-16.4%)	-25.2% (-46.4%-3.5%)	-37.9% (-48.7%--25.0%)
**Caribbean**	15.7% (11.9%-19.7%)	39.1% (33.1%-44.4%)	14.5% (10.8%-18.5%)	33.9% (27.6%-39.3%)	-8.0% (-26.9%-14.4%)	-13.3% (-20.6%--4.6%)
**Central Latin America**	15.2% (11.6%-19.1%)	36.5% (31.2%-41.4%)	7.7% (5.7%-9.9%)	21.2% (17.0%-25.2%)	-49.6% (-60.0%--38.5%)	-41.9% (-48.5%--35.3%)
**Tropical Latin America**	29.4% (23.4%-35.4%)	49.9% (42.8%-55.3%)	17.1% (13.1%-20.9%)	30.5% (24.3%-35.6%)	-41.9% (-51.2%--31.5%)	-38.8% (-45.6%--31.8%)
**North Africa and Middle East**	11.6% (7.4%-16.5%)	51.9% (44.7%-58.8%)	7.6% (5.5%-9.9%)	40.8% (34.2%-46.0%)	-34.8% (-51.8%--10.6%)	-21.4% (-29.2%--13.1%)
**South Asia**	7.1% (5.2%-9.2%)	40.4% (33.8%-45.9%)	6.8% (4.9%-8.7%)	32.1% (26.2%-37.0%)	-4.5% (-24.8%-18.8%)	-20.6% (-26.3%--14.8%)

**East Asia**	8.7% (6.0%-11.8%)	49.3% (43.3%-55.6%)	9.8% (7.6%-11.9%)	49.6% (44.4%-53.7%)	12.9% (-13.4%-49.0%)	0.5% (-8.6%-8.8%)
**Oceania**	13.4% (9.4%-18.0%)	33.6% (26.1%-41.4%)	11.4% (8.1%-15.0%)	26.4% (19.4%-33.5%)	-15.5% (-36.0%-15.8%)	-21.6% (-34.4%--7.1%)
**Southeast Asia**	7.9% (5.9%-10.2%)	50.8% (42.9%-56.4%)	6.5% (4.8%-8.3%)	46.1% (38.7%-51.9%)	-17.7% (-31.2%--1.3%)	-9.1% (-14.0%--4.3%)
**Central Sub-Saharan Africa**	2.3% (1.4%-3.4%)	22.4% (16.1%-28.5%)	1.8% (1.1%-2.6%)	18.0% (12.8%-23.1%)	-22.3% (-45.0%-7.8%)	-19.8% (-32.1%--5.0%)
**Eastern Sub-Saharan Africa**	5.3% (3.7%-7.0%)	25.8% (20.2%-30.8%)	4.7% (3.2%-6.2%)	19.6% (14.6%-24.5%)	-11.2% (-25.1%-4.1%)	-24.0% (-31.1%--16.0%)
**Southern Sub-Saharan Africa**	22.3% (16.7%-28.0%)	52.4% (44.3%-58.3%)	11.2% (7.9%-14.7%)	32.3% (24.5%-38.9%)	-49.6% (-60.4%--35.7%)	-38.4% (-46.9%--31.0%)
**Western Sub-Saharan Africa**	4.9% (2.8%-7.3%)	16.8% (11.7%-21.1%)	3.6% (2.1%-5.5% (100000	14.1% (9.9%-17.7%)	-26.7% (-48.6%-2.0%)	-16.0% (-24.8%--5.5%)

Data in parentheses are 95% uncertainty intervals.
SDI, socio-demographic index; PAF, population attributable fraction.
