# Supplementary material for: Burden of Peripheral Artery Disease and Its Attributable Risk Factors in 204 Countries and Territories From 1990 to 2019
Source: Front Cardiovasc Med. 2022 Apr 12;9:868370. doi: 10.3389/fcvm.2022.868370 (PMC9039520; doi:10.3389/fcvm.2022.868370)
Supplement: Supplementary file 5 [file Data_Sheet_1.pdf]

Text S1. Supplemental Methods

1.Estimation of deaths

Input data

Vital registration data were used to model peripheral artery disease (PAD). All datapoints with less than 1 death in Egypt were also marked as outliers in the present analysis.

Modelling strategy

Deaths due to PAD were estimated using a standard Cause of Death Ensemble Model (CODEm) approach, a highly systematized tool that runs many different models by location level predictive covariates and selects an ensemble of models that performs best. Predictive covariates incorporated in the ensemble models were summary exposure variable, systolic blood pressure, cholesterol, smoking prevalence, mean body mass index, Healthcare Access and Quality Index, diabetes fasting plasma glucose, lag distributed income per capita, Socio-demographic Index (SDI), omega-3, fruits, vegetables, nuts and seeds, pulses/legumes, polyunsaturated, fatty acids and alcohol. These covariates were selected based on a possible causality with PAD-specific mortality. All models for PAD-specific mortality were evaluated using out-of-sample predictive validity tests and combined into an ensemble of models that best reflects all the available input data.

2. Non-fatal Estimation

Input data

Survey data and claims data (inpatient visits and outpatient visits) were used in the estimation of non-fatal outcomes of PAD. We included claims data in the United States, but did not include any non-literature-based data types from any locations. We also did not use inpatient hospital data, because PAD is expected to be rare inpatient but common in outpatient as it is a condition usually managed on an outpatient basis, except for specific surgical interventions. This discrepancy leads to implausible correction factors on the basis of inpatient/outpatient information from claims data (~150X), hence adjusted data cannot be used. Including uncorrected data may lead to incorrect estimates as hospitalization and procedure rates are likely to vary between geographies based on access to and patterns of care.

Disability weights

PAD was divided into two severity levels: symptomatic and asymptomatic peripheral vascular disease. Each severity level was assigned a disability weight (DW), which reflects the severity of the disease on a scale from 0 (full health) to 1 (death).

Table. Severity levels of peripheral artery disease, and their lay descriptions and disability weight (DW)

Severity level	Lay description	DW (95% CI)
Asymptomatic	No symptoms	No DW assigned

Symptomatic	Has cramping pains in the legs after walking a medium<br>distance. The pain goes away after a short rest.	0.014 (0.007–0.025)

Modelling strategy

A Bayesian meta-regression tool, named DisMod-MR 2.1, was used to estimate the incidence and prevalence of PAD for each location, year, age and sex. We included the log-transformed, age-standardized summary exposure value scalar for PAD and log-transformed lag-distributed income as fixed-effect, country-level covariates. We included a value prior of 0 for incidence from ages 0 to 30. We also included a value prior of 0 for remission for all ages. Additionally, we included a value prior of 0 for excess mortality inbetween ages 0 and 30 as well as a value prior between 0 and 0.05 for excess mortality in between ages 30 and 100.

DisMod MR was also used to model the proportion of PAD with intermittent claudication. A value prior of 0 was set for proportion for ages 0 to 40. We included the Health Access and Quality Index score as a country-level covariate for excess mortality. The prevalence model was multiplied by the proportion model at the draw level to generate the prevalence of symptomatic and asymptomatic PAD.

Risk factors

Relative risk and exposure estimate for risk factors were extracted from all available data sources, including randomized controlled trials, cohort studies, household surveys, census data, and other data sources. A comparative risk assessment framework was used to estimate the proportions of PAD related deaths attributable to potentially modifiable risk factors. The counterfactual scenario of theoretical minimum was used to model the population attributable fraction, which represented the proportional reduction in age-standardized deaths that would occur if the exposure to a risk factor was reduced to the theoretical minimum exposure level. The theoretical minimum risk exposure level was defined as the lowest level of risk exposure below which its causation with a disease outcome is not accepted on the basis of available evidence.

Table. Definition and theoretical minimum risk exposure level for each risk factor of peripheral artery disease

**Risk factors**	**Definition**	**Theoretical minimum risk exposure level**
Diet high in sodium	24-h urinary sodium measured in g per day	24-h urinary sodium 1–5 g per day
High fasting plasma glucose	Serum fasting plasma glucose measured in mmol/L	4.8–5.4 mmol/L
High systolic blood pressure	Systolic blood pressure, measured in mmHg	110–115 mmHg

Kidney dysfunction	The kidney dysfunction risk factor exposure is divided into four categories of renal function defined by urinary albumin to creatinine ratio (ACR) and estimated glomerular filtration rate (eGFR):<br>• Albuminuria with preserved eGFR (ACR >30 mg/g & eGFR >=60 ml/min/1.73m2); this corresponds to stages 1 and 2 chronic kidney disease (CKD) in the Kidney Disease Improving Global Outcomes classification<br>• CKD stage 3 (eGFR of 30-59 ml/min/1.73m2);<br>• CKD stage 4 (eGFR of 15-29 ml/min/1.73m2);<br>• CKD stage 5 (eGFR <15ml/min/1.73m2, not (yet) on renal replacement therapy).	ACR 30 mg/g or less and eGFR greater than 60ml/min/1.73m2
Lead exposure	Blood lead levels in μg/dL of blood, bone lead levels in μg/g of bone	2 μg/dL, corresponding to lead levels in pre-industrial humans as natural sources of lead prevent the feasibility of zero exposure
Tobacco		
Smoking	Prevalence of current use of any smoked tobacco product and prevalence of former use of any smoked tobacco product; among current smokers, cigarette equivalents smoked per smoker per day and cumulative pack-years of exposure; among former smokers, number of years since quitting	All individuals are lifelong non smokers
Second-hand smoke	Average daily exposure to air particulate matter from second hand smoke with an aerodynamic diameter smaller than 2.5 μg, measured in μg/m³, among non-smokers	No second-hand smoke exposure

References

1. Global burden of 369 diseases and injuries in 204 countries and territories, 1990-2019: a systematic analysis for the Global Burden of Disease Study 2019. Lancet (London, England) 2020, 396(10258):1204-1222.
2. Global burden of 87 risk factors in 204 countries and territories, 1990-2019: a systematic analysis for the Global Burden of Disease Study 2019. Lancet (London, England) 2020, 396(10258):1223-1249.
3. Global age-sex-specific fertility, mortality, healthy life expectancy (HALE), and

population estimates in 204 countries and territories, 1950-2019: a comprehensive demographic analysis for the Global Burden of Disease Study 2019. Lancet (London, England) 2020, 396(10258):1160-1203.
