# Supplementary material for: Burden of Peripheral Artery Disease and Its Attributable Risk Factors in 204 Countries and Territories From 1990 to 2019
Source: Front Cardiovasc Med. 2022 Apr 12;9:868370. doi: 10.3389/fcvm.2022.868370 (PMC9039520; doi:10.3389/fcvm.2022.868370)
Supplement: Supplementary file 7 [file Image_2.PDF]

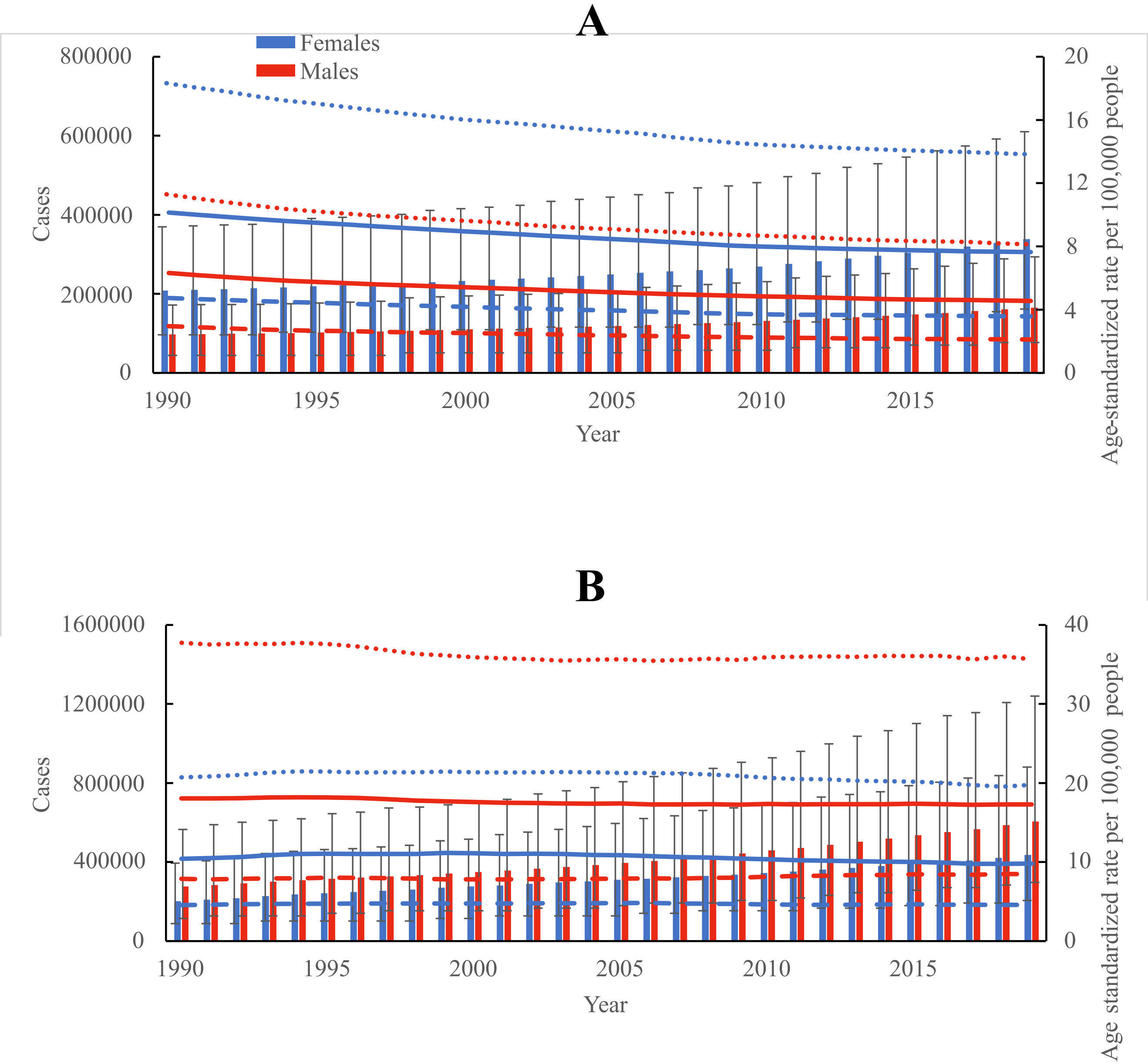

Figure S2. Trends in numbers and age-standardized rates of YLDs (A) and YLLs (B) of peripheral artery disease at the global level, 1990-2019. Dotted and dashed lines indicate 95% upper and lower uncertainty intervals, respectively.
